# Ashwin and FAM98 paralogs define nuclear and cytoplasmic RNA ligase complexes for tRNA biogenesis

**DOI:** 10.1038/s41467-026-77451-x

**Published:** 2026-09-17

**Authors:** Moritz Kleinwächter, Marius Moser, Nathan Raynaud, Moritz M. Pfleiderer, Martin Jinek, Javier Martinez

**Affiliations:** 1 Max Perutz Labs, Medical University of Vienna, Vienna BioCenter (VBC), Vienna, Austria; 2 Vienna BioCenter PhD Program, a Doctoral School of the University of Vienna and the Medical University of Vienna, Vienna, Austria; 3 UFR Sciences du Vivant, Université Paris Cité, Paris, France; 4 Department of Biochemistry, University of Zurich, Zurich, Switzerland

**Keywords:** RNA, Protein transport, Molecular biology

## Abstract

The tRNA ligase complex (tRNA-LC) seals tRNA exon halves in the nucleus during pre-tRNA splicing and *XBP1*-mRNA exons in the cytoplasm as part of the unfolded protein response (UPR). This dual function requires the tRNA-LC to be either nuclear or cytoplasmic. Here, we reveal that Ashwin (ASW), the vertebrate-specific subunit of the tRNA-LC, serves as its nuclear import factor. ASW contains a dual nuclear localisation signal (NLS) which, upon disruption, leads to the retention of the tRNA-LC in the cytoplasm, impairing pre-tRNA splicing with the consequent accumulation of 5’ tRNA fragments. We also show that the tRNA-LC exists in three forms, depending on which FAM98 paralog is bound, either FAM98A, FAM98B or FAM98C. ASW interacts exclusively with the FAM98B-containing complex, ensuring its nuclear localization for tRNA biogenesis. Attaching an NLS to RTCB, the catalytic and indispensable tRNA-LC subunit, rescues pre-tRNA splicing in cells depleted of ASW. We hypothesize that vertebrates evolved ASW to localize a sub-population of tRNA-LC to the nucleus, while using FAM98 paralogs to retain a fraction of RTCB in the cytoplasm to splice *XBP1*-mRNA during UPR.

## Abstract (web summary)

The tRNA ligase complex has both nuclear and cytoplasmic functions. Here, the authors reveal the existence of differentially localized tRNA ligase complex populations in human cells and identify the subunit ASW as the vertebrate-specific nuclear import factor of the complex.

## Introduction

Eukaryotic cells feature highly specialized compartments where specific functions are efficiently performed by macromolecular machineries mainly composed of proteins and RNA molecules. Since most proteins are synthesized in the cytoplasm, mechanisms relying on the recognition of specific amino acid sequences have evolved to ensure their correct, final destination. A dual localization has been observed for many proteins and protein complexes; however, a mechanistic understanding of how this is achieved is lacking for many of them. One such complex is the tRNA ligase complex (tRNA-LC), required in the cytoplasm to splice the *XBP1* mRNA during the Unfolded Protein Response (UPR), and in the nucleus to join tRNA exon halves during the splicing of precursor transfer RNAs (pre-tRNAs).

tRNAs are essential adaptor molecules for the translation of messenger RNAs (mRNAs) into proteins. Like most RNAs, tRNAs are synthesized as precursors (pre-tRNAs) that must undergo extensive processing to become functional. One such processing event is splicing (reviewed in refs. ^1–3^), a two-step reaction where a single intron is excised by the tRNA splicing endonuclease (TSEN) complex^4–6^ and the resulting tRNA exon halves are joined by an RNA ligase activity, generating a mature tRNA^7,8^. While only a subset of eukaryotic pre-tRNA genes contain introns^9,10^, splicing of those is essential for their function.

In mammals, the ligation of the tRNA exon halves is catalysed by the tRNA-LC, composed of the catalytic subunit RTCB, the DEAD-box helicase DDX1, CGI-99 (RTRAF/hCLE), FAM98B, and Ashwin (ASW/C2orf49)^11,12^ (reviewed in ref. ^13^). Two additional proteins are essential to sustain tRNA-LC activity: Archease assists the catalytic activity of RTCB by transferring GMP to an essential histidine residue^14,15^, and PYROXD1 protects RTCB against oxidative inhibition^16^. Components of the tRNA-LC, in particular RTCB and Archease, are also required for the splicing of the *XBP1* mRNA during UPR, a cellular stress response triggered by the accumulation of misfolded proteins in the endoplasmic reticulum (ER)^17–19^. During UPR, the *XBP1* mRNA is incompletely spliced in the nucleus into *XBP1u* mRNA (u for unspliced) and reaches the cytoplasm with a remaining 26-nt intron that is removed by the ER transmembrane endonuclease IRE1^20,21^ (reviewed in ref. ^22,23^). The tRNA-LC joins the resulting mRNA exons to generate *XBP1s* mRNA (s for spliced) which encodes a transcription factor that activates UPR genes.

Functionally, the human tRNA-LC can be split into a tetrameric core composed of RTCB, DDX1, FAM98B, and CGI-99, and a fifth subunit of hitherto unknown function, ASW, shown to assemble into the tRNA-LC by forming a subcomplex with FAM98B and CGI-99^24^. ASW is not required for the activity of the tRNA-LC in vitro^11^, but it is important for embryonic development in *X. laevis*, especially in neuronal tissues, by an unknown mechanism^25^. FAM98B is another subunit whose physiological function remains unclear. Recently, FAM98B was reported to be recruited to toxic poly-glycine aggregates by its glycine-rich C-terminal sequence, leading to depletion of the tRNA-LC and disruption of tRNA processing with implications for neurodegenerative pathologies^26^. FAM98B has two paralogs in human cells: FAM98A and FAM98C. Various functions in cancer^27–32^, viral infections^33^, RNA transport^34^, and cell migration^35^ have been reported for the three FAM98 paralogs, but whether FAM98A and FAM98C play any role within the tRNA-LC remains unknown.

In this report, we uncover the mechanism by which the tRNA-LC achieves both nuclear and cytoplasmic functions. We identify ASW as a vertebrate-specific subunit that confers nuclear localization to the tRNA-LC to execute pre-tRNA splicing. The nuclear localization relies on two nuclear localization signals (NLSs) that we dissect at the single amino acid level. We further show that FAM98A and FAM98C are present in tRNA-LC subpopulations; however, ASW specifically binds to FAM98B and therefore only FAM98B-containing tRNA-LC molecules reach the nucleus. Taken together, we have revealed the molecular mechanisms evolved to confer the tRNA-LC with a dual subcellular localization, allowing both nuclear and cytoplasmic functions in non-canonical RNA splicing.

## Results

### Paralogs of FAM98B define subpopulations of the human tRNA ligase complex

**Fig. 1:**
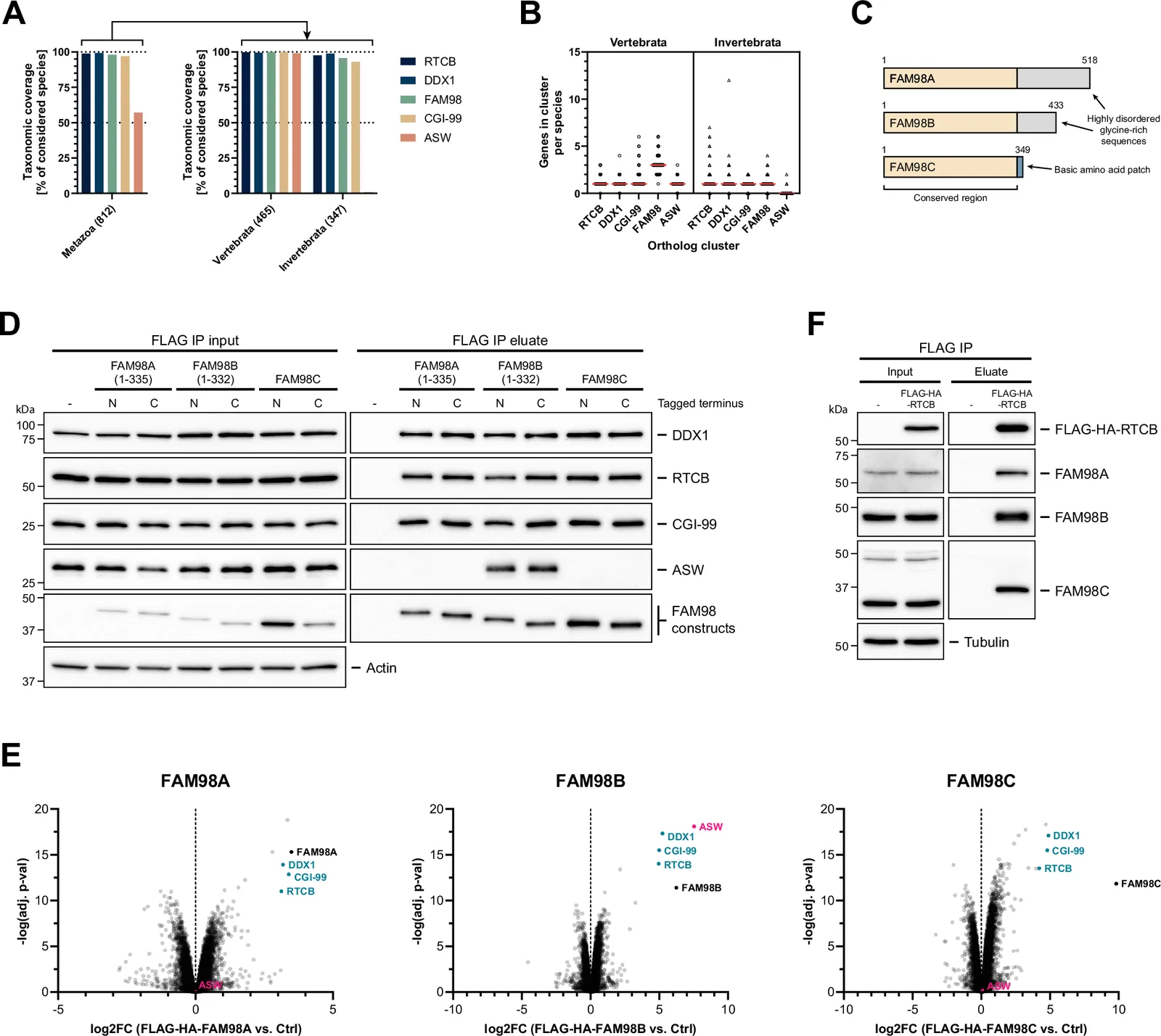
FAM98 paralogs evolved together with ASW in vertebrates to form different populations of tRNA ligase complexes. **A** Relative number of species having at least one ortholog of RTCB, DDX1, FAM98, CGI-99, or ASW. 812 metazoans were investigated, of which 465 were vertebrates, and 347 were invertebrates. Orthologous clusters as well as assigned species were retrieved from OrthoDB v11^36^. **B** Genes per species that are assigned to the orthologous clusters shown in (**A**). Red lines indicate the median. **C** Illustration of the three human FAM98 paralogs, highlighting regions that are conserved or different among them. **D** HEK FITR cell lines stably carrying doxycycline-inducible expression constructs for either N- or C-terminally FLAG-HA-tagged FAM98A(1-335), FAM98B(1-332), or FAM98C were treated with doxycycline for 24 h. As control, HEK FITR cells without any integrated construct (–) were used. The expressed constructs were isolated using an anti-FLAG IP. Input and eluate samples of the IP were analyzed by WB for DDX1, RTCB, CGI-99, ASW, FLAG, and ACTA1 (evaluation of equal loading). Equal amounts of total protein (input) or volume (eluate) were loaded per lane. **E** Anti-FLAG IP as in (**D**), but with HEK FITR cells expressing N-terminally FLAG-HA-tagged, full-length FAM98A, FAM98B, or FAM98C. After binding and washing, the beads were subjected to tandem mass spectrometry to identify and quantify bound proteins. The displayed protein enrichments are the results from three independent experiments. Statistical significance was calculated by performing two-sided t-tests and the resulting *p* values were adjusted with the Benjamini-Hochberg procedure. **F** Anti-FLAG IP as in (**D**), but with HEK FITR cells expressing FLAG-HA-RTCB. Input and eluate samples were analyzed by WB for FLAG, FAM98A, FAM98B, FAM98C, and Tubulin (evaluation of equal loading). Equal amounts of total protein (input) or volume (eluate) were loaded per lane. Source data are provided as a Source Data file.

In the past, we successfully used co-evolutionary analyses to identify two essential co-factors of RTCB: Archease^14^ and PYROXD1^16^. With renewed interest, we investigated the phylogenetic distribution of tRNA-LC subunits, analysing the occurrence of RTCB, DDX1, CGI-99, FAM98B and ASW in 812 metazoans from an existing orthologous cluster dataset^36^. While RTCB, DDX1, CGI-99, and FAM98B – core subunits of the tRNA-LC – occur widely throughout metazoans, ASW appears restricted to vertebrates (Fig. 1A). We also noticed that, of all considered species, the average copy number of genes in the FAM98 cluster is higher than for any other subunit. This increase is specific to vertebrates, with a median of three FAM98 genes per species, while invertebrates have a median of one (Fig. 1B). Our analysis indicates that, concurrently with the emergence of ASW, the FAM98 gene evolved into three paralogs of different length, FAM98A, FAM98B and FAM98C (Fig. 1C). FAM98C is the shortest paralog, and only FAM98A and FAM98B carry highly disordered glycine-rich sequences at the C-termini.

To test if all paralogs can assemble into the tRNA-LC, we expressed and immunoprecipitated (IP) FLAG-tagged versions of FAM98A, FAM98B, and FAM98C from HEK Flp-In T-REx (FITR) cells and performed western blots (WBs) against the remaining subunits of the human tRNA-LC (Fig. 1D and Supplementary Fig. 1A). Since FAM98A and FAM98B contain highly repetitive and disordered sequences at the C-termini, cloning and expression of these proved very difficult^26^. We thus decided to first generate and express truncated versions of FAM98A and FAM98B (Fig. 1D). We found all FLAG-tagged FAM98 paralogs able to associate with RTCB, DDX1, and CGI-99; however, ASW exclusively interacted with both truncated (Fig. 1D, right panel) and full-length (Supplementary Fig. 1A, right panel) forms of FAM98B. Mass spectrometry (MS) analysis confirmed that ASW is only enriched in FAM98B IPs (Fig. 1E and Supplementary Fig. 1B). We reciprocally detected all three FAM98 paralogs co-eluting with FLAG-tagged RTCB (Fig. 1F). Failing to detect a polypeptide of the right size for FAM98C (37 kDa) in whole cell lysates (Fig. 1F, FLAG IP input) is likely explained by its low abundance, estimated to be around 48 nM in HEK cells^37^, as well as the presence of cross-reacting polypeptides in whole cell lysates. We conclude that at least three different subpopulations of the tRNA-LC exist in human cells, containing either FAM98A, FAM98B or FAM98C, with ASW exclusively interacting with FAM98B-containing tRNA-LC. At odds with our findings, but adding value to our experimental approach, AlphaFold 3^38^ predicts that ASW should bind to all tRNA-LC isoforms, containing FAM98A, FAM98B, or FAM98C (Supplementary Fig. 2, 3). This suggests that the differences between the paralogs that ultimately allow or prevent ASW-binding might be very subtle. A concurrent manuscript by Pfleiderer et al. dissects these differences at the biochemical level^39^.

### FAM98B and ASW are required for efficient pre-tRNA splicing

**Fig. 2:**
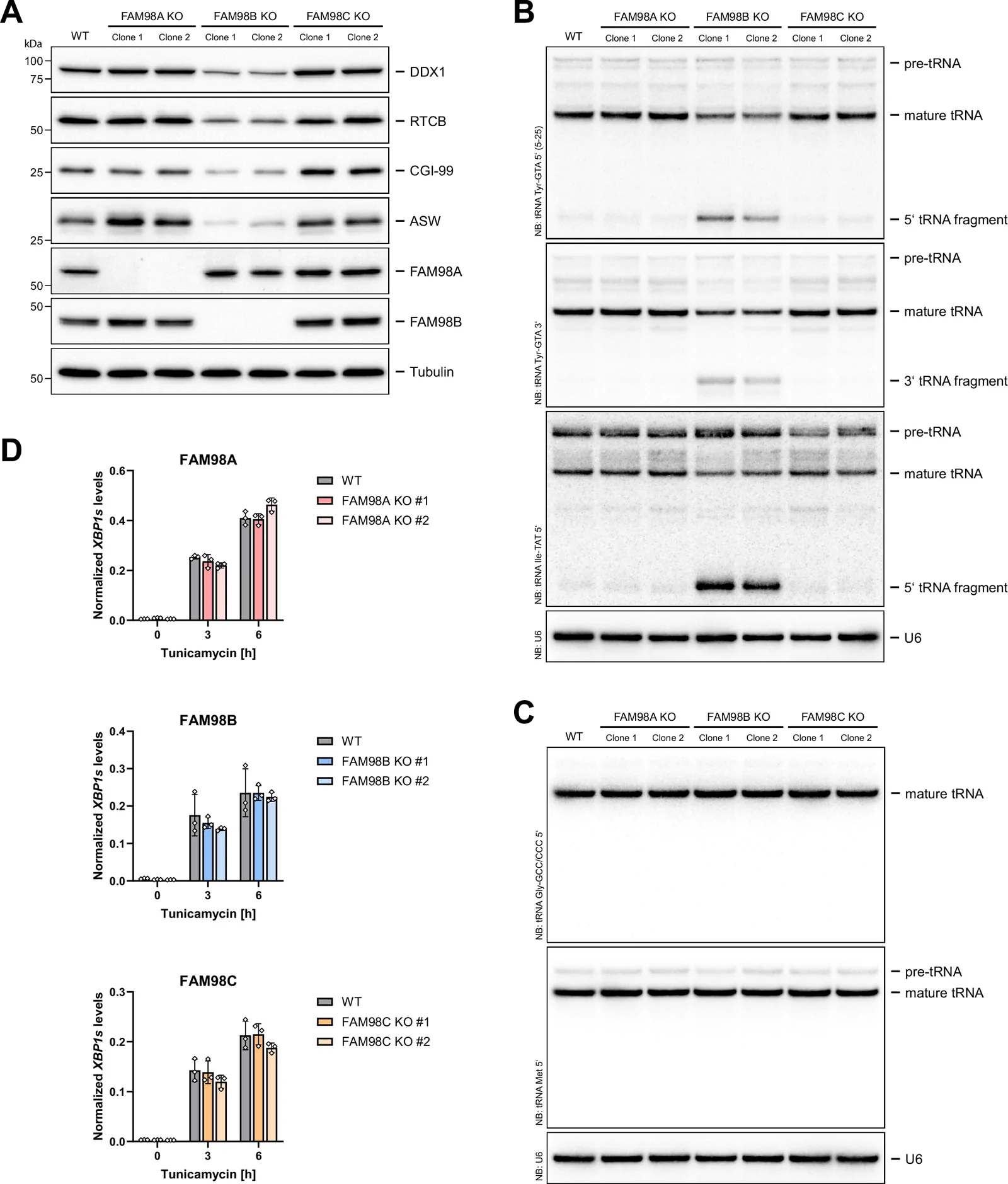
FAM98B is required for pre-tRNA splicing. **A** Whole cell lysates of HEK FITR WT, FAM98A KO, FAM98B KO, and FAM98C KO cells were analyzed by WB for levels of tRNA-LC subunits as well as Tubulin (evaluation of equal loading). Two clones were analyzed for each KO cell line. **B** Steady-state levels of tRNA Tyr-GTA and tRNA Ile-TAT as well as their precursors in HEK FITR WT, FAM98A KO, FAM98B KO, and FAM98C KO cells were determined by NB. 5 µg total RNA were loaded per lane. A probe against U6 snRNA was used as loading control. Two clones were analyzed for each KO cell line. **C** Same as in (**B**), but for tRNA Gly-GCC/CCC and tRNA Met. **D** Relative transcript levels of *XBP1s* (spliced) determined by qPCR during tunicamycin-induced UPR in HEK FITR WT, FAM98A KO, FAM98B KO, and FAM98C KO cells. Expression levels were normalized to *ACTB* mRNA levels. Relative expression levels were calculated with the 2^-ΔΔCt^ method^72^. Values represent means ± SD from three independent experiments. Two clones were analyzed for each KO cell line. Source data are provided as a Source Data file.

Having shown that the three FAM98 paralogs contribute to different tRNA-LCs, we wanted to understand their roles in the stability and enzymatic activity of the tRNA-LC. We therefore used CRISPR/Cas9 to delete the genes encoding FAM98A, FAM98B, and FAM98C. Deletion of FAM98B reduced the levels of DDX1, RTCB, CGI-99, and, more severely, ASW (Fig. 2A), while no changes were detected upon deletion of FAM98A and FAM98C. Unable to validate the knock-out (KO) of FAM98C through WBs, we turned to MS and IPs of FLAG-HA-RTCB to show efficient deletion of FAM98C (Supplementary Fig. 4A, B). We next tested the efficacy of different FAM98 paralogs to increase the levels of tRNA-LC subunits in FAM98B KO cells. Expression of truncated FAM98A, full-length and truncated FAM98B, as well as FAM98C restored the levels of CGI-99, RTCB, and DDX1, although to different extents (Supplementary Fig. 5). Overexpression of full-length FAM98A had no visible effect on the levels of other tRNA-LC subunits and, as expected, only re-expression of FAM98B could increase ASW levels.

We then tested whether depletion of any FAM98 paralog in HEK FITR cells would impair the ligation of tRNA exon halves during pre-tRNA splicing. We scored a potential defect in ligation via northern blotting (NB), monitoring the accumulation of tRNA fragments composed of 5’ leader and 5’ exon sequences (henceforth 5’ tRNA fragment), a phenomenon already observed when exposing cells to strong oxidants such as H_2_O_2_ and menadione^40^. Here, we detected an accumulation of 5’ tRNA fragments in both FAM98B KO clones, as recently reported by the Chivukula laboratory in HEK293T cells^26^, and a minor accumulation of 3’ tRNA fragments, with a simultaneous decrease in mature, spliced tRNAs (Fig. 2B and Supplementary Fig. 6A). In contrast, 5’ tRNA fragments did not accumulate in FAM98A or FAM98C KO clones, and mature tRNA levels remained intact. As expected, the absence of any FAM98 paralog had no impact on non-intron containing tRNAs, tRNA^Met-CAT^ and tRNA^Gly-CCC/GCC^ (Fig. 2C and Supplementary Fig. 6A). Despite a decrease in mature, spliced tRNAs, FAM98B KO cells did not display a consistently reduced growth rate, but both FAM98A and FAM98B KO cell lines showed longer lag phases (Supplementary Fig. 6B, C). Finally, neither cytoplasmic *XBP1*-mRNA splicing nor cellular protein synthesis were affected by any of the FAM98 KOs (Fig. 2D and Supplementary Fig. 6D, E).

**Fig. 3:**
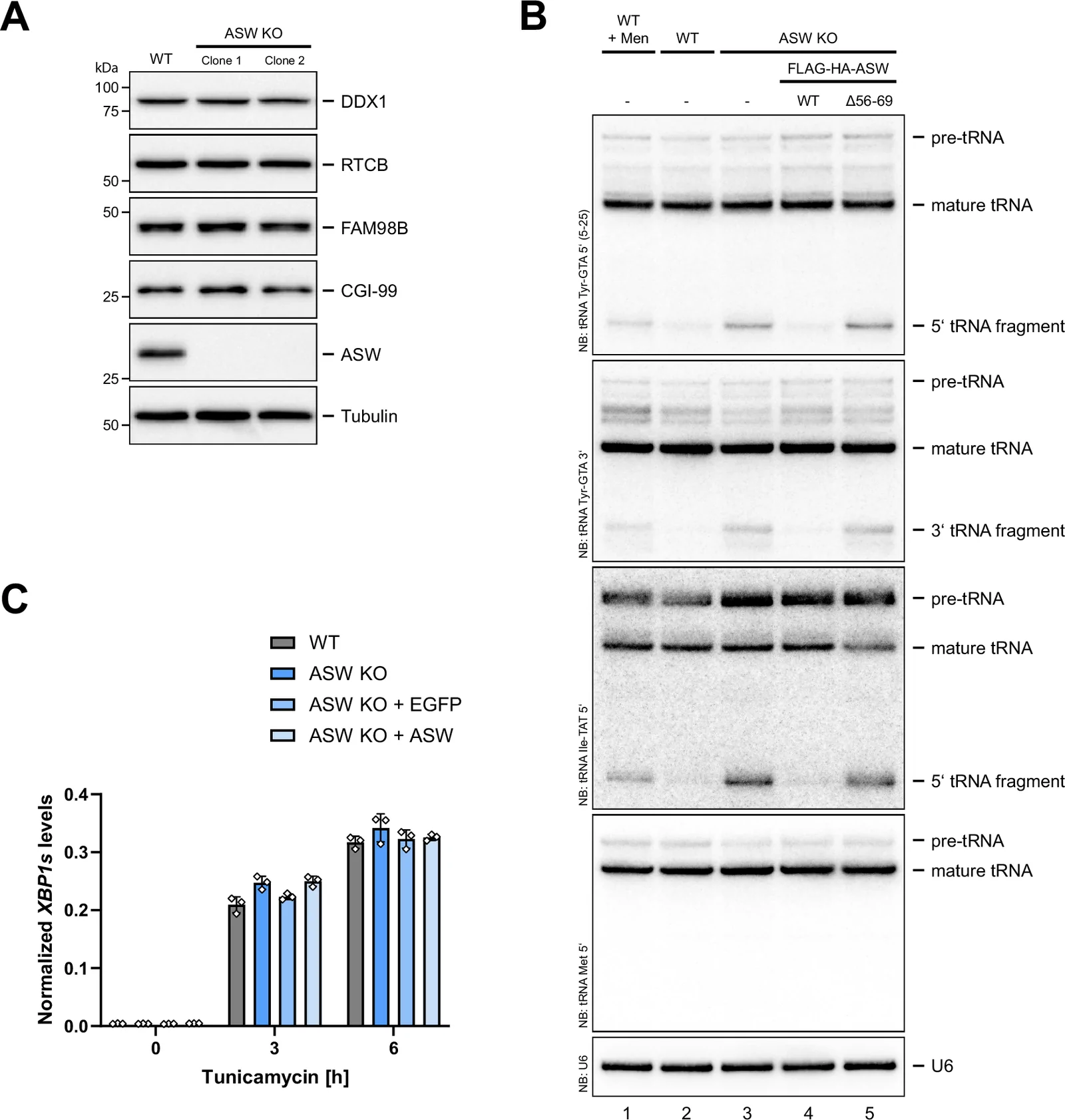
Loss of ASW leads to a pre-tRNA splicing defect. **A** Whole cell lysates of HEK FITR WT and ASW KO cells (2 clones) were analyzed by WB for levels of tRNA-LC subunits as well as Tubulin (evaluation of equal loading). **B** Steady-state levels of tRNA Tyr-GTA, tRNA Ile-TAT, and tRNA Met as well as their precursors in HEK FITR WT, ASW KO and rescued ASW KO cells were determined by NB. As a positive control for inhibition of the tRNA-LC, WT cells were treated with 40 µM menadione for 1 h prior to RNA isolation (lane 1). The rescued cells had the rescue constructs stably integrated and expressed by the addition of doxycycline for 48 h prior to RNA isolation. 5 µg total RNA were loaded per lane. A probe against U6 snRNA was used as loading control. **C** Relative transcript levels of *XBP1s* (spliced) determined by qPCR during tunicamycin-induced UPR in HEK FITR WT, ASW KO, and rescued ASW KO cells. The rescued cells had the rescue constructs stably integrated and expressed by the addition of doxycycline for 24 h prior to RNA isolation. Expression levels were normalized to *ACTB* mRNA levels. Relative expression levels were calculated with the 2^-ΔΔCt^ method^72^. Values represent means ± SD from three independent experiments. Source data are provided as a Source Data file.

With ASW assembling into the tRNA-LC in exclusive association with FAM98B, we wondered whether the defective pre-tRNA splicing observed in FAM98B KO cells is actually due to the concurrent absence of ASW, whose deletion had no impact on the other subunits of the tRNA-LC (Fig. 3A)^11,14^. Indeed, we also detected accumulation of 5’ tRNA fragments in ASW KO cells, similar to those detected upon inhibition of the tRNA-LC with menadione (Fig. 3B, lanes 1–3)^40^. However, the defect was milder than in FAM98B KO cells, as we did not observe a decrease in the levels of mature, spliced tRNAs (Fig. 3B and Supplementary Fig. 7A). Overexpression of FLAG-tagged WT ASW rescued pre-tRNA splicing, confirming that the defect is caused by the absence of ASW (Fig. 3B, lane 4). However, a mutant of ASW unable to associate with the tRNA-LC due to the deletion of residues 56 to 69 (Δ56-69) (Supplementary Fig. 7B) did not abrogate the accumulation of the 5’ tRNA fragments (Fig. 3B, lane 5), indicating that ASW supports pre-tRNA splicing via its association with the tRNA-LC. As in FAM98B KO cells, *XBP1*-mRNA splicing remained unaffected in ASW KO cells (Fig. 3C) and we did not observe anomalies in cell growth (Supplementary Fig. 7C, D).

### ASW localizes the tRNA ligase complex to the nucleus through two independent nuclear localization signals

**Fig. 4:**
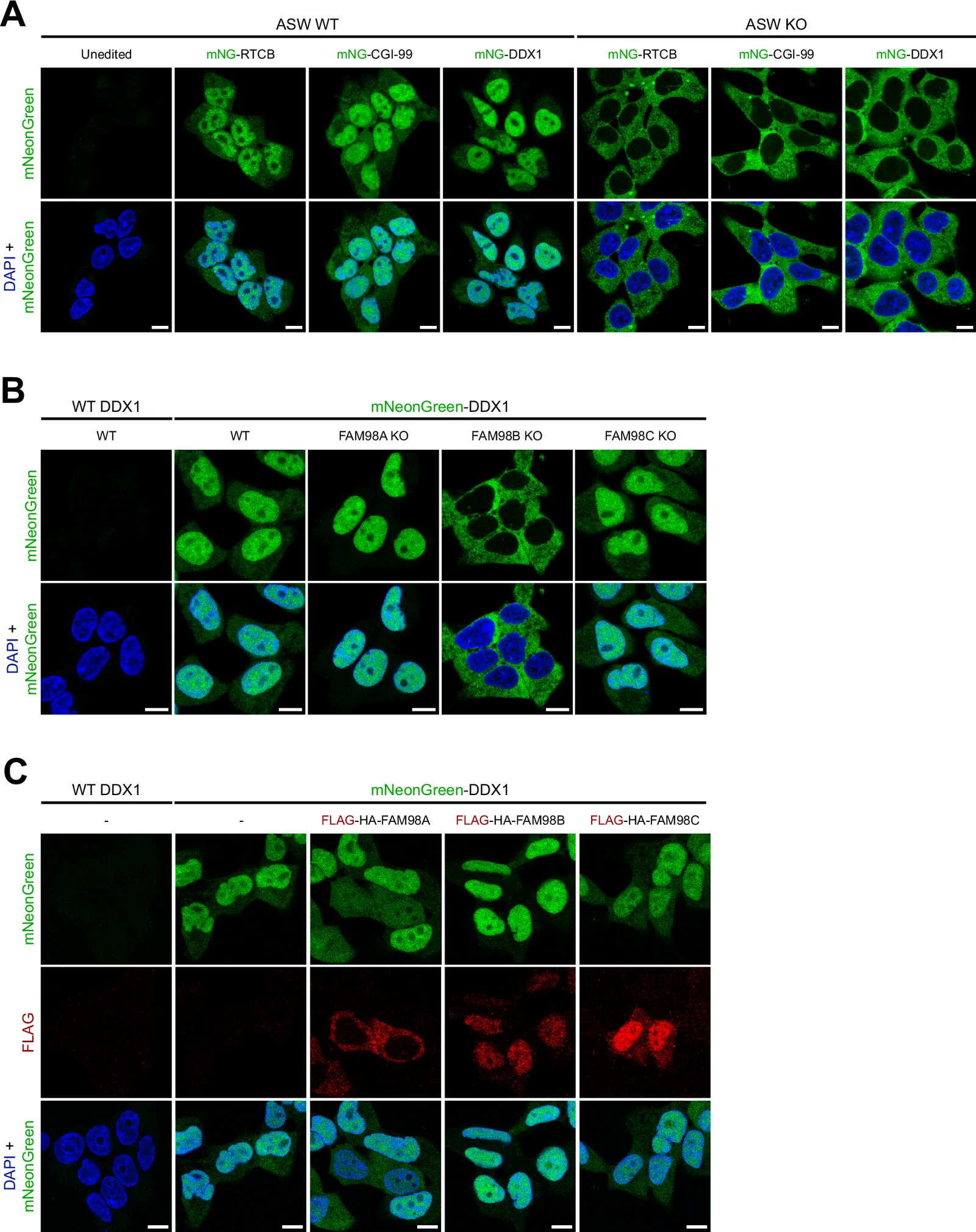
ASW and the FAM98 proteins define the subcellular localization of the tRNA-LC. **A** HEK FITR WT and ASW KO cells expressing endogenous mNeonGreen-RTCB, mNeonGreen-CGI-99, or mNeonGreen-DDX1 were fixed, permeabilized, and stained with DAPI, to determine the localization of endogenous tRNA-LC by microscopy. HEK FITR WT cells were used as negative control. **B** HEK FITR WT, FAM98A KO, FAM98B KO and FAM98C KO cells expressing endogenous mNeonGreen-DDX1 were fixed, permeabilized and stained with DAPI, to determine the localization of endogenous tRNA-LC by microscopy. HEK FITR WT cells were used as negative control. **C** HEK FITR WT cells endogenously expressing mNeonGreen-DDX1 and stably carrying doxycycline-inducible expression constructs coding for FLAG-HA-tagged FAM98A, FAM98B, or FAM98C were treated with doxycycline for 24 h. Anti-FLAG immunofluorescence was performed and cells were stained with DAPI, to determine the localization of endogenous tRNA-LC as well as the expressed constructs by microscopy. As control, HEK FITR WT cells and HEK FITR WT cells endogenously expressing mNeonGreen-DDX1 without any integrated construct (–) were used. **A–C** Scale bars indicate 10 μm.

Intrigued by the defective pre-tRNA splicing in cells deprived of ASW and the apparent dispensability of ASW for UPR – similar to cells deprived of FAM98B – we speculated that ASW might play a role in the subcellular localization of the tRNA-LC. Pre-tRNA splicing has been shown to occur in the nucleus of animal cells^4,41,42^ while the UPR-induced splicing of *XBP1* mRNA takes place in the cytoplasm^20,43^. Considering the subcellular locations of these processes, and in light of the reported, but untested potential NLSs in ASW^25^, we hypothesized that ASW might localize the tRNA-LC to the nucleus. Thus, we tagged endogenous RTCB, CGI-99, and DDX1 at the N-terminus with mNeonGreen (mNG)^44^ using CRISPR/Cas9, in both HEK FITR WT and ASW KO cells (Supplementary Fig. 8). We detected mNG-RTCB, mNG-CGI-99, and mNG-DDX1 mainly in the nucleus but also in the cytoplasm of WT cells (Fig. 4A), in agreement with a recent report on the spatial organization of the proteome^37^. In sharp contrast, mNG-RTCB, mNG-CGI-99, and mNG-DDX1 were barely detectable in the nucleus of ASW KO cells but rather accumulated in the cytoplasm, indicating that ASW is indeed required for the nuclear localization of the tRNA-LC (Fig. 4A). Expression of FLAG-tagged WT ASW, but not ASW Δ56-69, re-imported the tRNA-LC to the nucleus (Supplementary Fig. 9A, B).

We further wondered how the subcellular localization of the tRNA-LC would change upon perturbing the levels of the different FAM98 paralogs. We therefore tagged endogenous DDX1 at the N-terminus with mNG using CRISPR/Cas9, in HEK FITR FAM98A, B, and C KO cells. Loss of FAM98A led to a severe depletion of cytoplasmic tRNA-LC, while loss of FAM98B led to the nuclear exclusion of the tRNA-LC, phenocopying the loss of ASW. In contrast, loss of FAM98C had no significant effect on the localization of the tRNA-LC (Fig. 4B and Supplementary Fig. 9C). To test whether overexpression of FAM98 paralogs would alter the localization of endogenous tRNA-LC, we expressed FLAG-tagged FAM98A, B, or C in HEK FITR WT cells endogenously expressing mNG-DDX1 and imaged the localization of the constructs as well as the endogenous tRNA-LC. We detected FLAG-tagged FAM98B and C mainly in the nucleus, while FAM98A was only detected in the cytoplasm (Fig. 4C). Most strikingly, overexpression of FAM98A shifted the balance of endogenous tRNA-LC from the nucleus to the cytoplasm (Fig. 4C and Supplementary Fig. 9D). Contrary to that, overexpression of FAM98B led to a slight reduction in cytoplasmic tRNA-LC. Even though we found FAM98C mostly in the nucleus, its overexpression caused a slight decrease in nuclear tRNA-LC. Our data suggests that FAM98A-containing tRNA-LC is strictly cytoplasmic and that controlled expression of the different FAM98 paralogs balances the distribution of the tRNA-LC between the cytoplasm and nucleus.

**Fig. 5:**
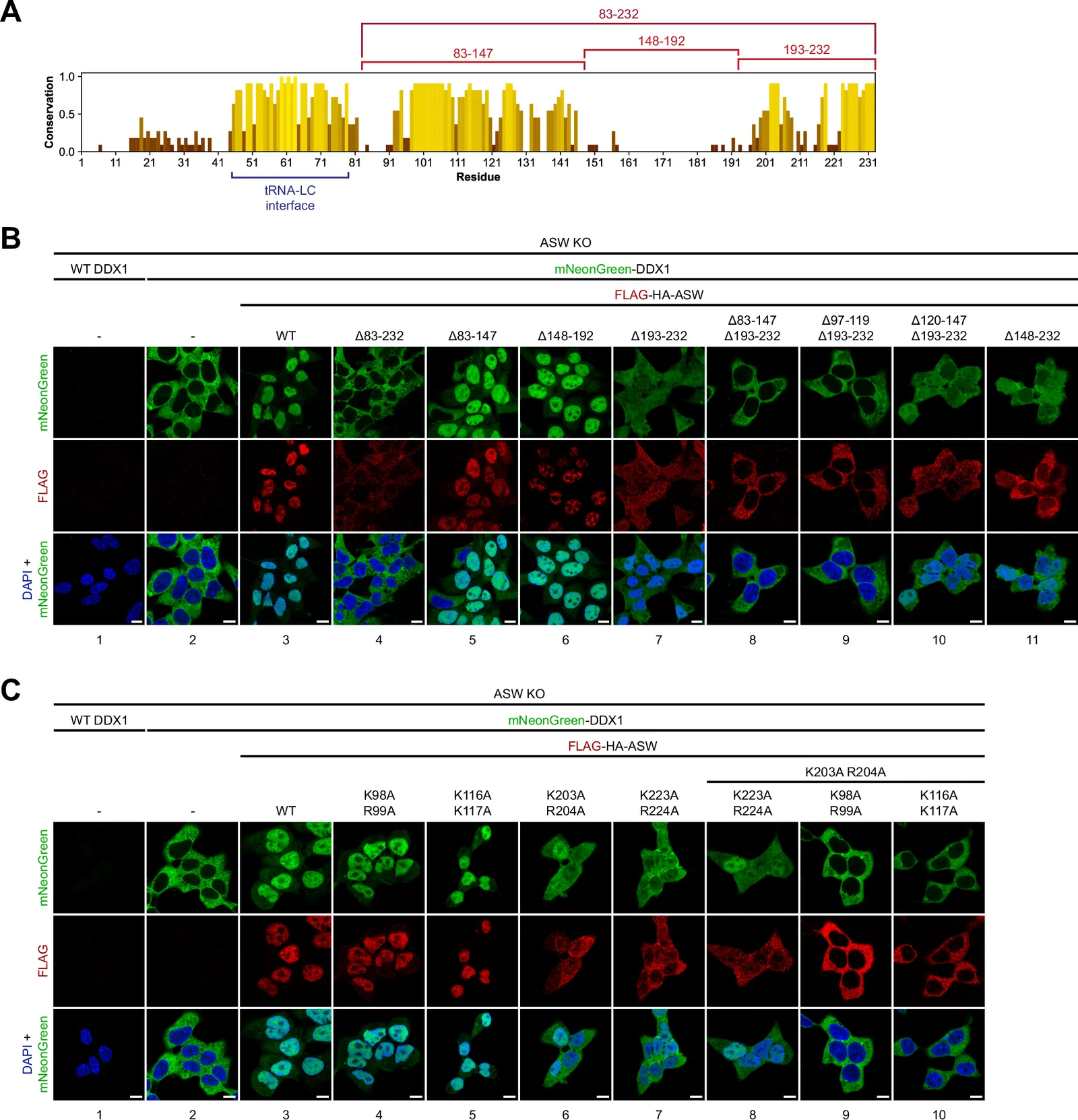
ASW localizes the tRNA-LC to the nucleus via two NLSs that require basic residues. **A** Conservation of human ASW derived from a multiple sequence alignment^58^ of 465 vertebrate ASW sequences. Source data are provided as a Source Data file. **B** HEK FITR ASW KO cells endogenously expressing mNeonGreen-DDX1 and stably carrying doxycycline-inducible expression constructs for different FLAG-HA-tagged ASW constructs (WT and truncation mutants) were treated with doxycycline for 24 h. Anti-FLAG immunofluorescence was performed and cells were stained with DAPI, to determine the localization of endogenous tRNA-LC as well as the expressed constructs by microscopy. As control, HEK FITR ASW KO cells and HEK FITR ASW KO cells endogenously expressing mNeonGreen-DDX1 without any integrated construct (–) were used. **C** Same as in (**B**), but with different point mutants of ASW instead of truncation mutants. **B**,** C** Scale bars indicate 10 μm.

To identify the amino acid sequences in ASW responsible for the nuclear localization of the tRNA-LC, we generated truncation mutants of FLAG-tagged ASW by removing most of the highly conserved regions (Fig. 5A). Expressing those mutants in HEK FITR ASW KO cells endogenously expressing mNG-DDX1 revealed NLSs in the last two-thirds of the coding sequence of ASW (residues 83-232), downstream of the identified tRNA-LC interface (Fig. 5B, panel 4). We further split the Δ83-232 mutant into three smaller truncations (panels 5–7) and found that only the very C-terminal truncation Δ193–232 (panel 7) impaired the localizing function, although not to the extent of the Δ83–232 mutant. We next combined the Δ193–232 mutant with other truncations to identify the remaining part of the NLS (panels 8-11). Here, only a combination of Δ193–232 with Δ83–147 (panel 8), which we could further restrict to Δ193–232 with Δ97–119 (panel 9), was able to fully abrogate the ability of ASW to localize the tRNA-LC to the nucleus, indicating that ASW harbors functional NLSs only in the regions between residues 97–119 and 193–232. We further confirmed that the ASW Δ83–232 mutant, unable to rescue the nuclear localization, is still able to bind the tRNA-LC (Supplementary Fig. 10A).

To further define the NLSs in ASW, we used NLS prediction algorithms on the sequence of the protein. The tool NLSExplorer^45^ predicted four NLS segments, all lying within the regions we had identified to be necessary for nuclear import (Supplementary Fig. 10B). Assuming that we were dealing with canonical NLSs that consist of basic amino acid clusters, we mutated arginine and lysine residues into alanine in the predicted NLSs as well as in other highly conserved parts of the central and C-terminal region of ASW (Supplementary Fig. 10C). While most mutants were still able to localize the tRNA-LC efficiently to the nucleus (Fig. 5C, panels 4 and 5; and Supplementary Fig. 10D), two mutants in the C-terminal region – K203A R204A and K223A R224A – partially lost this ability (Fig. 5C, panels 6 and 7). Similar to the truncation mutants, neither those mutants alone nor combined into a single construct were able to completely abrogate the nuclear localization (panel 8). However, combining the K203A R204A mutant with NLS mutants in the 97–117 region – K98A R99A or K116A K117A – resulted in complete loss of nuclear localization (panels 9 and 10).

We further tested whether the identified NLSs of ASW can localize an NLS reporter construct (GST-mNG-mNG) to the nucleus and found that both tested regions, ASW 97–117 and 201–225, can independently act as NLSs (Supplementary Fig. 11A). We therefore conclude that ASW harbors two functional NLSs, with NLS1 spanning residues 97–117 and NLS2 spanning residues 201–225, and that the function of both NLSs relies on highly conserved basic residues.

### The tRNA ligase complex must be localized to the nucleus to execute pre-tRNA splicing

**Fig. 6:**
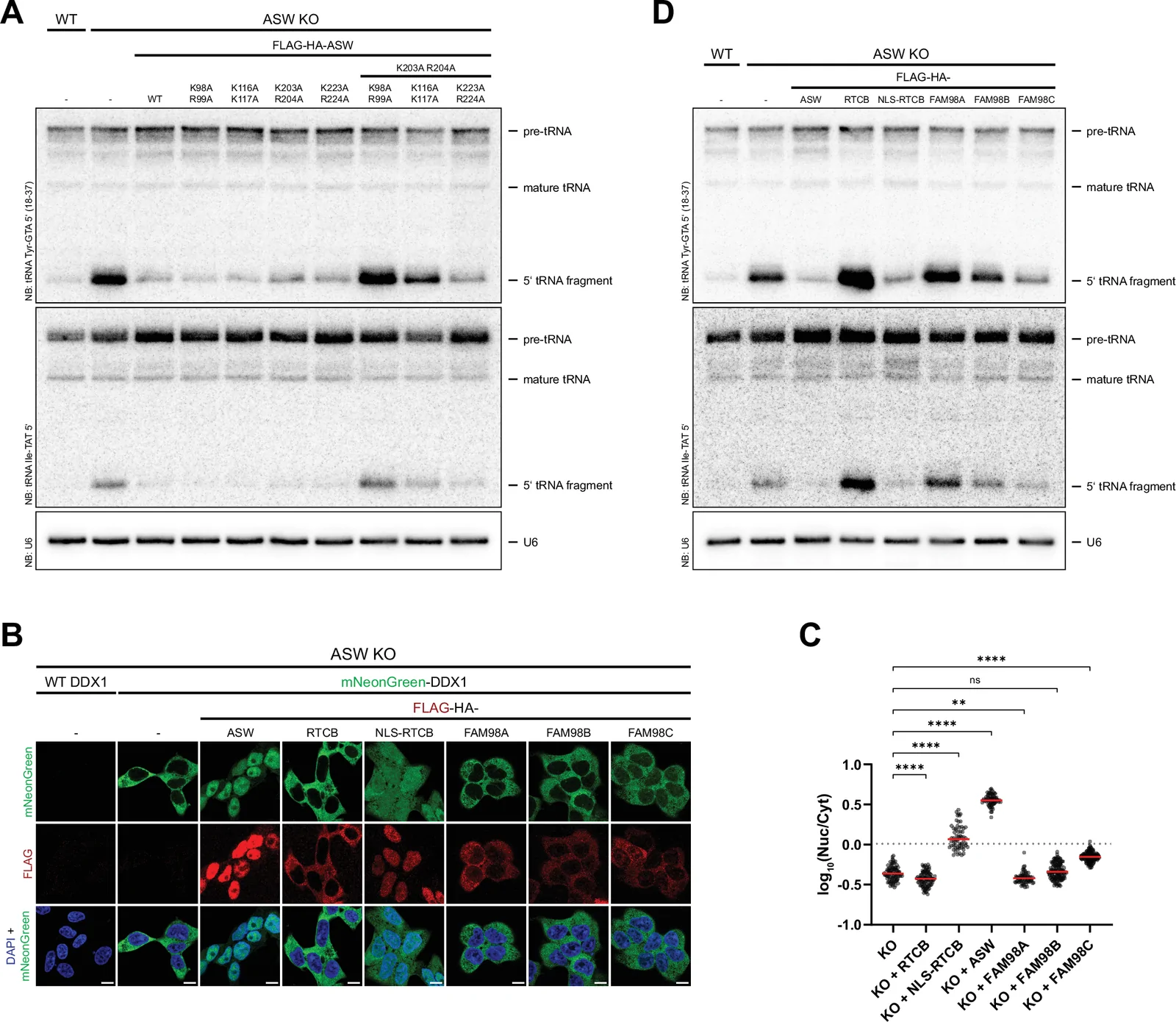
Nuclear localization of the tRNA-LC is essential for efficient pre-tRNA splicing. **A** Steady-state levels of tRNA Tyr-GTA and tRNA Ile-TAT as well as their precursors in HEK FITR WT, ASW KO and rescued ASW KO cells were determined by NB. The rescue cell lines stably carried doxycycline-inducible expression constructs for different FLAG-HA-tagged ASW constructs (WT and point mutants) and were treated with doxycycline for 48 h prior to RNA isolation. 5 µg total RNA were loaded per lane. A probe against U6 snRNA was used as loading control. **B** HEK FITR ASW KO cells endogenously expressing mNeonGreen-DDX1 and stably carrying doxycycline-inducible expression constructs for different FLAG-HA-tagged constructs (ASW, RTCB, c-MYC-NLS-RTCB, FAM98A, FAM98B, FAM98C) were treated with doxycycline for 24 h. Anti-FLAG immunofluorescence was performed and cells were stained with DAPI, to determine the localization of endogenous tRNA-LC as well as the expressed constructs by microscopy. As control, HEK FITR ASW KO cells and HEK FITR ASW KO cells endogenously expressing mNeonGreen-DDX1, without any integrated construct (–) were used. Scale bars indicate 10 μm. **C** Quantification of (**B**). The logarithm of the ratio between the mean nuclear and mean cytoplasmic signal of mNeonGreen-DDX1 was used to determine the nuclear-cytoplasmic distribution of the tRNA-LC. Red lines indicate the mean. Each dot represents a single quantified cell of the indicated sample. Number of quantified cells: 83 (KO), 91 (KO + RTCB), 63 (KO + NLS-RTCB), 69 (KO + ASW), 64 (KO + FAM98A), 130 (KO + FAM98B), 141 (KO + FAM98C). Indicated *P* values: ns *P* = 0.1076, ** *P* = 0.0012, **** *P* < 0.0001 (one-way ANOVA, Dunnett’s test). (**D**) NBs as described in (**A**), but with the constructs that were used for the microscopy experiments in (**B**). Source data are provided as a Source Data file.

We next tested whether impairing the nuclear localization of the tRNA-LC by mutating the NLSs of ASW would cause the previously observed defect in pre-tRNA splicing. We overexpressed FLAG-tagged WT ASW as well as truncation and NLS mutants in ASW KO cells and monitored the accumulation of 5’ tRNA fragments (Fig. 6A and Supplementary Fig. 12). With striking correlation, all mutants that were unable to redirect the tRNA-LC to the nucleus, were also unable to abrogate the accumulation of 5’ tRNA fragments (compare Fig. 5B, C with Fig. 6A and Supplementary Fig. 12). However, even ASW mutants that only partially restored the nuclear localization of the tRNA-LC (Δ193-232 in Fig. 5B; and mutants K203A R204A and K223A R224A in Fig. 5C) nearly completely abolished the accumulation of 5’ tRNA fragments (Fig. 6A and Supplementary Fig. 12).

We performed further rescue experiments in ASW KO cells by overexpressing FLAG-tagged FAM98A, B, or C, as well as FLAG-tagged WT RTCB and FLAG-tagged RTCB to which we added the c-MYC NLS (NLS-RTCB)^46^. While overexpression of WT RTCB did not re-localize the tRNA-LC to the nucleus, overexpression of NLS-RTCB did, although not completely (Fig. 6B, C). Overexpression of FAM98C showed a mild increase in the amount of nuclear tRNA-LC, which was neither observed for FAM98A nor for FAM98B. We validated these findings by once again monitoring the accumulation of 5’ tRNA fragments and found a positive correlation between nuclear exclusion of the tRNA-LC and their accumulation: overexpression of NLS-RTCB and FAM98C abrogated the accumulation of 5’ tRNA fragments in ASW KO cells almost similar to when overexpressing WT ASW (Fig. 6D). Interestingly, overexpression of WT RTCB or FAM98A increased the accumulation of 5’ tRNA fragments, possibly due to a reinforced nuclear exclusion of the tRNA-LC (Fig. 6C, D). Thus, our data indicates that pre-tRNA splicing requires the tRNA-LC to be present in the nucleus. While Ashwin has evolved to perform this function, we show that the tRNA-LC can still execute pre-tRNA splicing when reaching the nucleus by other means, such as artificially adding an NLS to RTCB or by increasing the levels of the FAM98C-containing tRNA-LC.

We further wondered how FAM98C might achieve nuclear localization of the tRNA-LC and suspected that its very C-terminal sequence, which ends with 7 basic residues (RKKKKKK), might encode an NLS. Thus, we evaluated the C-terminal sequence of FAM98C (residues 328-349) with the GST-mNG-mNG NLS reporter that we used for validating the NLSs of ASW. The reporter localized to both cytoplasm and nucleus, but surprisingly accumulated in nucleoli (Supplementary Fig. 11B). While the accumulation in nucleoli does not match the observed localization of FLAG-tagged FAM98C (Figs. 4C and 6B), it indicates that the C-terminus of FAM98C is at least able to provide nuclear localization.

**Fig. 7:**
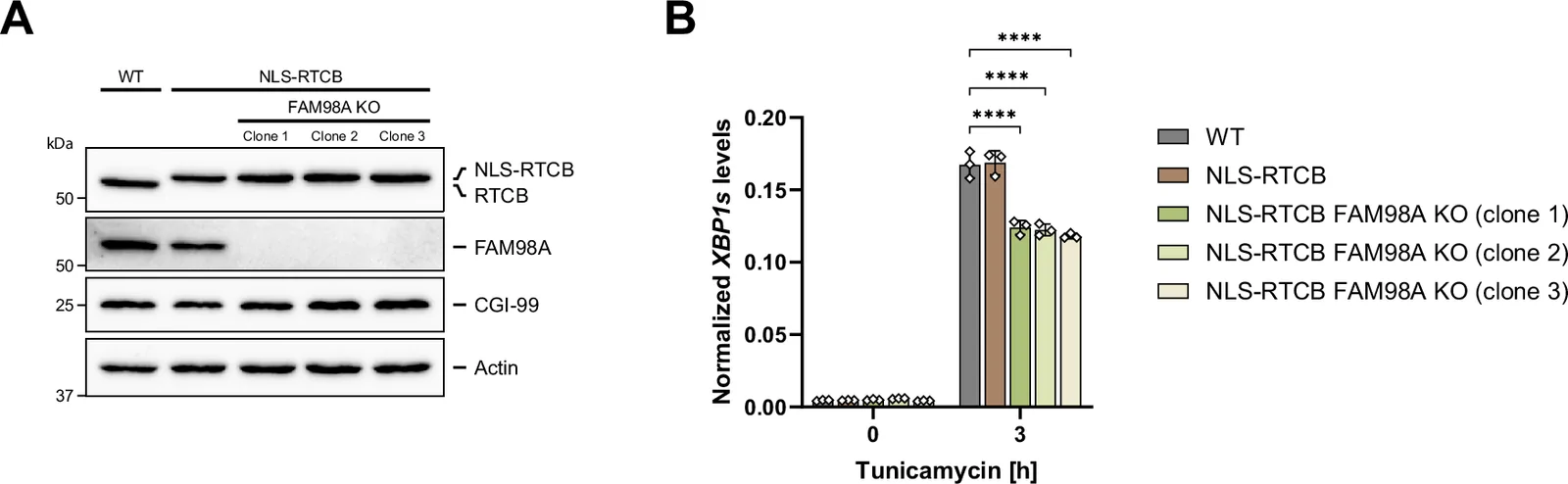
Adding an NLS onto endogenous RTCB while FAM98A is depleted leads to reduced *XBP1*-mRNA splicing during the unfolded protein response. **A** Whole cell lysates of HEK FITR WT, NLS-RTCB, and NLS-RTCB FAM98A KO cells were analyzed by WB to validate the editing of endogenous RTCB and the KO of FAM98A. Actin was used for evaluation of equal loading. Three clones were analyzed for the NLS-RTCB FAM98A KO cell line. **B** Relative transcript levels of *XBP1s* (spliced) determined by qPCR during tunicamycin-induced UPR in the cell lines that are shown in (**A**). Expression levels were normalized to *ACTB* mRNA levels. Relative expression levels were calculated with the 2^-ΔΔCt^ method^72^. Values represent means ± SD from three independent experiments. All indicated *P* values (****) < 0.0001 (two-way ANOVA, Dunnett’s test). Source data are provided as a Source Data file.

Finally, we wanted to address the spatial requirements of the tRNA-LC to execute *XBP1*-mRNA splicing, which is known to take place in the cytoplasm. Since depletion of FAM98A had no effect on *XBP1*-mRNA splicing, despite the clear reduction in cytoplasmic tRNA-LC levels, we speculated that minimal amounts of tRNA-LC could remain in the cytoplasm and be sufficient to perform *XBP1*-mRNA splicing. In such scenario, we suspected that nuclear import of the tRNA-LC might be incomplete or too slow due to limited levels of endogenous ASW. We therefore overexpressed ASW in FAM98A KO cells. However, *XBP1*-mRNA splicing remained fully functional (Supplementary Fig. 13). Aiming to achieve a more severe depletion of cytoplasmic tRNA-LC without overexpressing ASW, we used CRISPR/Cas9 to add the c-MYC NLS onto the N-terminus of endogenous RTCB and further to delete FAM98A from the c-MYC-NLS-RTCB cell line (Fig. 7A). These cells displayed reduced *XBP1*-mRNA splicing (Fig. 7B). Interestingly, the sole addition of the c-MYC NLS onto endogenous RTCB was not sufficient to cause a defect, indicating that FAM98A might prevent nuclear import of the tRNA-LC.

In summary, we have shown that all human FAM98 paralogs, FAM98A, B, and C, can alternatively assemble into the tRNA-LC, defining tRNA-LC subpopulations that differ in their localization. We found FAM98A to be cytoplasmic, while FAM98B localizes to the nucleus. ASW, which exclusively binds to FAM98B-containing tRNA-LC, enables the nuclear localization of the complex through two independent, conserved NLSs. Loss of FAM98B or ASW results in defective pre-tRNA splicing through the nuclear exclusion of the tRNA-LC. This defect is stronger when FAM98B is absent, likely due to the additional depletion of other tRNA-LC subunits. Restoring the nuclear localization of the tRNA-LC through other means than ASW, either FAM98C overexpression or adding an NLS to RTCB, also rescued pre-tRNA splicing, indicating that nuclear localization of the tRNA-LC, independently of how it is achieved, is what matters for efficient pre-tRNA splicing. Curiously, and deserving further investigation, *XBP1*-mRNA splicing is resilient towards the absence of any FAM98 protein or ASW and was only reduced when we added an NLS to endogenous RTCB while depleting FAM98A.

## Discussion

The advent of vertebrates coincided with the emergence of ASW and the diversification of FAM98 into three paralogs, FAM98A, B, and C, leading to differentially localized sub-populations of tRNA-LC. Here, we have shown that ASW provides nuclear localization to the tRNA-LC in strict association with FAM98B, thereby allowing pre-tRNA splicing. The nuclear importing function of ASW relies on two independent NLSs, whose critical residues are highly conserved throughout all analysed ASW homologues. Both NLSs show the characteristics of a bipartite NLS, having two short basic amino acid clusters that are separated by a linker region. While the linker regions are unusually long – 14 and 18 residues – and therefore do not fit the definition of canonical bipartite NLSs^47^, it was shown that bipartite NLSs with longer linker regions exist and can be functional^48^.

We observed large differences in the expression behavior of human FAM98 paralogs, with FAM98A and FAM98B being less stable than FAM98C. The long and aggregation-prone, glycine-rich tails of FAM98A and, to a larger extent, FAM98B^26^ might dictate this behavior; removing them equalized their expression. However, considering that FAM98B makes up the majority of the FAM98 proteins in the investigated cell line, the observed effects on stability could have been an artifact caused by overexpression-induced aggregation of FAM98B, which has recently been reported to cause neurodegenerative diseases^26^. Whether the different tails of the FAM98 proteins have any physiological role is yet to be determined.

Removing FAM98B from HEK cells caused a severe co-depletion of all other tRNA-LC subunits, even stronger than previously reported using siRNAs against FAM98B^11^ (see also^26^). Structural studies on the assembly of the tRNA-LC showed that FAM98B forms a dimer with CGI-99, which then binds RTCB^49,50^. We assume that both FAM98A and FAM98C should assemble equally well into the different tRNA-LCs, as the CGI-99 and RTCB interfaces are conserved between all FAM98 proteins. However, the FAM98 proteins affect the steady-state levels of the tRNA-LC in different ways. Only FAM98B and FAM98C expression restored the levels of the complex in FAM98B KO cells, while FAM98A was neutral in this regard. This would also explain why depleting FAM98A does not lead to a decrease in the levels of other tRNA-LC subunits. However, this might also depend on the cell line, as shRNA-mediated depletion of FAM98A was shown to reduce the levels of other core subunits in A549 cells^33^. The removal of FAM98C not impacting other subunits to an extent that is detectable by WB is likely explained by its low expression level in HEK cells, estimated to be around 48 nM^37^.

While we have unveiled a function of the FAM98B/ASW-containing tRNA-LC, the roles of the other tRNA-LC populations remain partially unclear. Hinted by our taxonomic analysis, we believe that the occurrence of ASW, together with the emergence of several FAM98 paralogs, evolved to support additional functions or regulatory layers for the tRNA-LC, one being the subcellular distribution of the different complexes. We propose that splicing of *XBP1* mRNA during the UPR is preserved by always keeping a certain number of tRNA-LC molecules in the cytoplasm. Our data suggests that this is mainly enforced by FAM98A, as we found the localization of FAM98A-containing tRNA-LC to be restricted to the cytoplasm. Complementary work by Pfleiderer et al., dissecting the structural framework of human tRNA-LC assembly^39^, supports this idea by showing that the overexpression of FAM98A leads to a slight defect in pre-tRNA splicing. This is likely caused by retaining large proportions of tRNA-LC in the cytoplasm due to FAM98A out-competing FAM98B for assembly into the tRNA-LC, as we have observed in our own experiments. However, since *XBP1*-mRNA splicing remains fully functional upon complete removal of FAM98A, we believe that a fraction of FAM98B- or FAM98C-containing tRNA-LCs also remains in the cytoplasm, which is sufficient to perform this task. Disrupting *XBP1*-mRNA splicing by changing the subcellular localization of the tRNA-LC proved to be difficult. Only adding an NLS onto endogenous RTCB, while simultaneously depleting FAM98A, led to a mild defect. Together with our other results, this implies that fully functional *XBP1*-mRNA splicing requires only a minimal amount of cytoplasmic tRNA-LC and that nuclear import by ASW and FAM98C is incomplete. This further raises the question whether FAM98A evolved to assure *XBP1*-mRNA splicing or rather to keep some tRNA-LC in the cytoplasm for a yet unknown function. Beyond differences in subcellular distribution, all three mouse FAM98 paralogs have been shown to play roles in osteoclastogenesis^51^. A specialized function of FAM98 paralogs in bone homeostasis would certainly align with their exclusive occurrence in vertebrates. However, which roles the tRNA-LC subpopulations play in such processes is unknown and would require further investigation.

Of all FAM98 paralogs, only the loss of FAM98B caused a defect in pre-tRNA splicing. This defect led to a reduction of mature, spliced tRNAs, but caused no global protein synthesis defect. Loss of FAM98B further had a negative, but still mild effect on cell growth. We believe that the pre-tRNA splicing defect in FAM98B KO cells is mainly caused by the tRNA-LC losing its nuclear localization, which is worsened by the co-depletion of all other tRNA-LC subunits. HEK cells lacking ASW also displayed a defect in pre-tRNA splicing, but neither showed a detectable reduction in levels of mature, spliced tRNAs, nor displayed reduced viability. We surmise that the phenotype is milder in ASW KO cells, as only the nuclear localization of the tRNA-LC is lost, but the levels of its core components are not reduced. We further hypothesize that a small amount of tRNA-LC might reach the nucleus independently of ASW and provide enough tRNA ligase activity to maintain pools of mature tRNAs. In fact, we have seen that a minimal amount of nuclear tRNA-LC is sufficient to rescue the observed splicing defect and we have shown that FAM98C allows the tRNA-LC to reach the nucleus. We therefore suspect that FAM98C might compensate to some extent for the loss of ASW. However, since FAM98C KO cells do not display a defect in pre-tRNA splicing, ASW would still remain the main driver for the nuclear localization of the tRNA-LC in HEK cells. This might not be true for other cell lines or tissues, where FAM98C might be expressed at higher levels than ASW. It is interesting that knocking-out FAM98B in adult mice leads to a reduction of mature, spliced tRNAs in the brain, but not in the liver, ultimately causing cerebellar pathology but no hepatotoxicity^26^. Protein levels of ASW and FAM98C differ between cerebellum and liver; ASW levels are high in the cerebellum and low in the liver, and the opposite goes for FAM98C^52,53^. This might explain why the cerebellum is more affected by the loss of FAM98B/ASW, while the liver might be able to compensate this loss with FAM98C.

Contrary to the lack of a strong phenotype in our experiments, it was shown that controlled levels of ASW are essential for cell survival and correct tissue patterning during embryogenesis of *X. laevis*^25^. This suggests that functions of ASW might be specifically needed in the context of tissue formation in multicellular organisms and could thus explain why our simplified tissue culture model fails to show severe phenotypes in the absence of ASW.

Very recently, C2orf49 (ASW) was identified together with ESF1 and ZNF117 as a differentially expressed gene and candidate biomarker for obstructive sleep apnea (OSA) and sarcopenia^54^. In addition, higher levels of a long non-coding RNA antisense to the ASW transcript (C2orf49-DT) was found associated to colorectal cancer and patients with poor prognosis^55^.

In turn, it remains to be determined how nuclear localization of the tRNA-LC is achieved in invertebrates. While there is previous evidence showing that pre-tRNA splicing in vertebrates takes place in the nucleus^4,41,42^, mechanistic insights into the subcellular localization of this process are missing for invertebrates, where other mechanisms may allow for nuclear translocation of the tRNA-LC.

Surprisingly, we did not observe a decrease in *XBP1*-mRNA splicing in cells lacking either ASW or any FAM98 protein. Loss of DDX1 was recently also shown to be innocuous towards *XBP1*-mRNA splicing^56^. These observations prompt the idea that *XBP1*-mRNA splicing might require no other subunit than RTCB – or no function provided by them although being present – excepting Archease and PYROXD1, essential factors for catalysis and protection of the tRNA-LC against oxidative stress^14,16,56^.

It has recently been shown that RTCB, together with other factors, participates in SOS splicing both in human cells and in *C. elegans*^57^. SOS splicing prevents DNA transposition by acting at the mRNA level, removing transposable elements through excision by a yet unidentified endonuclease, followed by ligation by RTCB. Based on our results, we hypothesize that SOS splicing, if a nuclear event, should be impaired in the absence of FAM98B or ASW.

FAM98B was recently shown to aggregate in fragile X-associated tremor/ataxia syndrome (FXTAS) and neuronal intranuclear inclusion disease (NIID), both neurodegenerative disorders linked to expanded GGC-repeats. These aggregates cause depletion of tRNA-LC and concomitant pre-tRNA splicing defects^26^. While that study supports that FAM98B-containing tRNA-LC is nuclear, we provide a mechanistic explanation of how this subpopulation reaches the nucleus and that ASW is ultimately the subunit enabling efficient pre-tRNA splicing due to its nuclear localizing function.

## Methods

### Cell culture, transfections and UPR-induction

Cells were cultured in complete DMEM [DMEM (Thermo, 41966029) supplemented with 10 % fetal calf serum (Thermo, 10270), 100 U/mL penicillin, 100 μg/mL streptomycin (Lonza, DE17-602E) and 0.2 M HEPES (Thermo, 15630080)] at 37 °C and 5 % CO_2_. Cells were regularly tested for mycoplasma contamination. For transient transfections, cells were transfected using Lipofectamine 3000 (Thermo, L3000015), following the manufacturer’s instructions. For experiments that required induction of UPR, we incubated the cells with 1.5 µg/mL tunicamycin for 3 or 6 h.

### Taxonomic and conservational analysis

The following orthologous cluster datasets were downloaded from OrthoDB v11^36^: 2522645at33208 (RTCB), 125852at33208 (DDX1), 5614at33208 (RTRAF/CGI-99), 7650at33208 (FAM98), and 2482041at33208 (ASW). We also retrieved a list of all metazoans that were analyzed by OrthoDB v11 and further split this list into invertebrates and vertebrates. For calculating the taxonomic coverage of the investigated orthologous clusters, we determined the percentage of species (metazoans, vertebrates, or invertebrates) that had at least one gene in the corresponding orthologous cluster. We further determined how many assigned genes each species had per investigated orthologous cluster. For conservational analysis of ASW, we performed a multiple sequence alignment of all protein sequences in the ASW cluster with Clustal Omega^58^. From the alignment, we then determined conservation scores for each residue with the software JalView 2.11.5.1^59^. As we were interested in the conserved residues of human ASW, we omitted all position of the alignment that corresponded to gaps in the sequence of human ASW.

### AlphaFold 3 predictions

All AlphaFold 3 (v3.0.0) predictions of the different tRNA-LCs were performed using the AlphaFold Server (https://alphafoldserver.com/)^38^ with protein sequences retrieved from UniProt (https://www.uniprot.org/)^60^: Q9BVC5-1 (ASW), Q9Y224 (CGI-99), Q9Y3I0 (RTCB), Q92499-1 (DDX1), Q8NCA5 (FAM98A), Q52LJ0-2 (FAM98B), and Q17RN3-1 (FAM98C). Used seeds for the predictions: 353676726 (FAM98A tRNA-LC), 1576571727 (FAM98B tRNA-LC), and 818145509 (FAM98C tRAN-LC). The predicted structures were visualized with ChimeraX 1.11^61^.

### Construct cloning and mutagenesis

All expression constructs were cloned into the pcDNA5/FRT/TO vector (Thermo, V652020) under a Tet-On-controlled CMV promotor. Constructs were assembled from gel-purified PCR products or from synthesized DNA fragments using NEBuilder HiFi DNA Assembly Master Mix (NEB, E262), according to the manufacturer’s instructions. PCR fragments were amplified using Q5 Hot Start High-Fidelity DNA Polymerase (NEB, M0493), either using cDNA or existing lab-internal plasmids as template. Primers for site-directed mutagenesis of constructs were designed with NEBaseChanger (https://nebasechanger.neb.com/) and the linear mutagenized constructs were generated with Q5 Hot Start High-Fidelity DNA Polymerase (NEB, M0493) from the non-mutagenized constructs. For circularization of linear mutagenized constructs, 2 μL of the unpurified PCR product was mixed with 2 μL 10x rCutSmart (NEB, B6004), 2 μL 10 mM ATP, 1 μL 100 mM DTT, 1 μL T4 DNA Ligase (NEB, M0202), 0.2 μL T4 PNK (NEB, M0201), 0.2 μL DpnI (NEB, R0176) and 11.6 μL nuclease-free water. The circularization reaction was performed in a thermocycler: 15 min at 37 °C, followed by 5 min at 23 °C. Cloning reactions, both from general cloning and mutagenesis, were transformed into competent NEB 5-alpha *E. coli* cells (NEB, C2987) and resulting colonies were further grown as liquid cultures, from which plasmid DNA was isolated. All generated constructs were validated by Sanger sequencing.

### Stable cell line generation (Flp-In T-Rex 293 cells)

To generate stable cell lines, HEK Flp-In T-REx 293 cells (Thermo, R78007) were co-transfected with the pOG44 vector (Thermo, V600520) and a pcDNA5/FRT/TO vector (Thermo, V652020) carrying the construct to be integrated under a Tet-On-controlled CMV promotor. As negative control for the selection, only the pOG44 vector was transfected. Two days after the transfection, the cells were split into selection medium [complete DMEM with 100 µg/mL Hygromycin B (Sigma, H3274)]. The selection was stopped after a minimum of two weeks and when all cells in the negative control were dead. The selected cells were then tested for construct expression upon doxycycline addition (1 μg/mL) and further passaged as usual. In experiments, construct expression was induced by adding doxycycline to a final concentration of 1 μg/mL.

### Cell growth analysis

Cells were seeded into 12-well plates (50,000 cells in 2 mL medium per well) and incubated over night for reattachment. The plates were then transferred into an Incucyte live-cell analysis system (Sartorius) and imaged over a period of 6 days (9 individual phase-contrast images per well, every 4 h). We used the Incucyte AI Confluence algorithm to determine the confluence of the cells on each acquired image. The data from the center image of each well was not used for further analysis, as the cells slightly accumulate in this part of the well during seeding. The measured confluences from the other 8 images of each well were then averaged for each timepoint. We used the R package Growthcurver 0.3.1^62^ with R 4.4.1 to fit logistic curves to our measured growth data. The determined growth rate corresponds to the parameter r of the fitted curve. Statistical analysis was performed using GraphPad Prism 10.5.0.

### Puromycin-incorporation assay

Cells were seeded in a 6-well plate and grown to 80 % confluence (per replicate: three wells of WT, one well for each FAM98 KO clone). The medium was exchanged and the cells were incubated for 2 h. One well with WT cells was then treated with cycloheximide (10 μg/mL) and the cells were further incubated for 90 min. Puromycin was added to each well, except one of the untreated WT wells, to a final concentration of 10 μg/mL. After 10 min of incubation, the cells were put on ice, the medium was removed and the cells were washed once with ice-cold PBS. Cell lysates were prepared, separated by SDS-PAGE and blotted onto a PVDF membrane. Western blots for puromycin-labeled proteins and GAPDH (redeveloped from the same membrane) were performed. For quantification, we used Fiji 2.16.0^63^ to measure the intensities of the puromycin-labeled-proteins (per lane) as well as the GAPDH bands. Within a replicate, the puromycin intensities were first normalized to the loading control (GAPDH) and then further normalized to the control sample (WT + puromycin). Statistical analysis was performed using GraphPad Prism 10.5.0.

### Cell lysate preparation and immunoprecipitation

Medium was removed, the cells were put on ice and were washed once with ice-cold PBS (Thermo, 14190094). The cells were resuspended by adding ice-cold PBS and pipetting several times up and down. The cells were pelleted at 1000 × *g*, 4 °C for 1 min. The supernatant was completely removed and the pellet was resuspended in a small volume of lysis buffer (30 mM HEPES pH 7.3, 100 mM KCl, 5 mM MgCl_2_, 104 µM AEBSF, 1.0 % NP-40, 10 % Glycerol). The crude lysate was rotated at 18 rpm for 30 min at 4 °C and then pelleted at 21,100 × *g* and 4 °C for 10 min. The cleared lysate (supernatant) was transferred into a new tube and the total protein concentration was measured with a Bradford assay (Biorad, 5000006). The lysate was diluted to the desired concentration with lysis buffer. All lysates that belonged to the same experiment were diluted to the same concentration. Anti-FLAG M2 magnetic beads (Sigma, M8823) were equilibrated by washing them three times with 1 mL ice-cold lysis buffer. For binding, the prepared lysates were added to the beads and incubated for 2 h at 4 °C rotating at 18 rpm. For all samples that belonged to the same experiment, equal amounts of beads as well as lysate was used. A small volume of lysate was not added to the beads and kept as input sample. After the binding, the beads were collected with a magnetic separator and the supernatant was removed. The beads were washed five times with 1 mL ice-cold lysis buffer. For elution, ice-cold lysis buffer containing 0.5 mg/mL 3xFLAG peptide was added and mixed by shortly vortexing on the highest setting. The beads were then incubated at 4 °C in a shaker at 1200 rpm for 45 min. The beads were pelleted at 500 g for 1 min at 4 °C and were then collected with a magnetic separator. The eluate was transferred into a new tube. 5x SDS loading dye (5 % SDS w/v, 62.5 mM Tris pH 6.8, 50 % Glycerol v/v, 25 mM EDTA, 0.025 % Bromophenol blue w/v, 5% β-Mercaptoethanol v/v) was added to the eluates as well as the input samples to a final concentration of 1x. The samples were mixed, boiled at 98 °C for 1 min and then stored at –20 °C until further analysis.

### Western blotting

Samples in 1x SDS loading dye were boiled for 1 min at 98 °C, mixed and then separated by SDS-PAGE. Polyacrylamide gels were blotted onto methanol-activated PVDF membranes (Biorad, 1620175) using Trans-Blot SD Semi-Dry Electrophoretic Transfer Cells (Biorad, 1703940) and Trans-Blot Turbo Transfer Buffer (Biorad, 10026938). Membranes were then blocked in 5% milk in PBS-T (PBS with 0.05 % Tween-20) for 1 h on a shaker. The blocking solution was removed, diluted primary antibody was added and incubated at 4 °C on a shaker overnight (ON). The antibody solution was removed and the blot was washed three times with 20 mL PBS-T for 10 min at RT. Secondary antibody diluted 1:5000 in 5 % milk PBS-T was added to the membrane and incubated for 1 h at RT on a shaker. The antibody solution was removed and the membrane was washed three times with 20 mL PBS-T for 10 min at RT. The blot was developed with Clarity Western ECL Substrate (Biorad, 1705061) and imaged with a ChemiDoc Touch (Biorad, 1708370). Used primary antibodies: anti-RTCB (1:2000; Bethyl Laboratories, A305-079A), anti-DDX1 (1:2000; Bethyl Laboratories, A300-521A), anti-FAM98A (1:1000; Abcam, ab204083), anti-FAM98B (1:1000; Thermo Fisher Scientific, PA5-52568), anti-FAM98C (1:1000; Thermo Fisher Scientific, PA5-59356), anti-CGI-99 (1:2000; Abcam, ab188326), anti-ASW (1:100; self-made), anti-Puromycin (1:5000; Sigma Aldrich, ZMS1016), anti-Tubulin (1:2000; Abcam, ab44928), anti-ACTA1 (1:1000; Sigma Aldrich, A2066), anti-GAPDH (1:2000; Cell Signaling, 2118), anti-FLAG (1:1000; Sigma Aldrich, F1804). Used secondary antibodies: anti-rabbit HRP-conjugated (1:5000; Invitrogen, 65-6120), anti-mouse HRP-conjugated (1:5000; Invitrogen, 62-6520).

### Co-IP/MS experiments

For the co-IP/MS experiments, the bait constructs were immunoprecipitated as described above, but were washed eight times in detergent-free lysis buffer. The beads, with bound proteins, were resuspended in 30 µL 2 M urea and 50 mM ammonium bicarbonate and were then digested with 150 ng LysC (MS grade, FUJIFILM Wako chemicals) at RT for 90 minutes. The supernatant was transferred to a new tube. The beads were rinsed with 30 µL 50 mM ammonium bicarbonate and combined with the supernatant. Disulfide bonds were reduced with 2.4 µL of 250 mM DTT for 30 min at RT before adding 2.4 µL of 500 mM iodoacetamide and incubating for 30 min at RT in the dark. The remaining iodoacetamide was quenched with 1.2 µL of 250 mM DTT for 10 min. Proteins were digested with 150 ng trypsin (Trypsin Gold, Promega) in 1.5 µL 50 mM ammonium bicarbonate overnight. The digest was stopped by the addition of trifluoroacetic acid (TFA) to a final concentration of 0.5 %, and the peptides were desalted using C18 Stagetips^64^. LC-MS analysis was performed on an UltiMate 3000 RSLCnano LC system (Thermo Scientific) coupled to a timsTOF HT (Bruker). The system was equipped with a CaptiveSpray ion source (Bruker), and a Butterfly Portfolio Heater (Phoenix S&T). Peptides were loaded onto a trap column (Acclaim PepMap 100 C18 HPLC Column, 5 mm × 0.3 mm, 5 μm particle size, Thermo Scientific) using 0.1 % TFA as mobile phase, and separated on an analytical column (Aurora Ultimate XT C18, 25 cm × 75 µm, 1.7 µm particle size, IonOpticks), applying a linear gradient starting with a mobile phase of 98 % solvent A (0.1 % FA) and 2 % solvent B (80 % acetonitrile, 0.08 % FA), increasing to 35 % solvent B over 60 min at a flow rate of 300 nL/min. The analytical column was heated to 50 °C. The mass spectrometer was operated in data-independent acquisition (DIA) parallel accumulation serial fragmentation (PASEF) mode^65^. MS2 data were acquired with eight PASEF scans per duty cycle, each containing three m/z windows. The m/z window widths were adjusted based on the expected precursor density, covering a total range of 300–1200 m/z. The ion mobility range was set to 0.64-1.42 V*s/cm, and the accumulation and ramp time was set to 100 ms. TIMS elution voltages were calibrated linearly to obtain the reduced ion mobility coefficients (1/K0) using three Agilent ESI-L Tuning Mix ions (m/z 622, 922, and 1222). Collision energy for fragmentation was scaled linearly with precursor mobility (1/K0), ranging from 20 eV (at 1/K0  =  0.6 V*s/cm) to 59 eV at (at 1/K0  =  1.6 V*s/cm). Raw MS data were converted to htrms format using HTRMS converter (Biognosys). Then it was searched with Spectronaut (19.1, Biognosys) in directDIA+ mode against the Uniprot human reference proteome (version 2024_01, www.uniprot.org), as well as a database of most common contaminants (version 2023_03, https://github.com/maxperutzlabs-ms/perutz-ms-contaminants). The search was performed with full trypsin specificity and a maximum of two missed cleavages at a protein and peptide spectrum match false discovery rate of 1 %. Carbamidomethylation of cysteine residues was set as fixed, oxidation of methionine, and N-terminal acetylation as variable modifications. The cross-run normalization was turned off and all other settings were left as default. Computational analysis was performed using Python and the in-house developed Python library MsReport (version 0.0.25, https://zenodo.org/records/16677904). LFQ protein intensities reported by Spectronaut were log2-transformed and normalized across samples using the ModeNormalizer from MsReport. The missing normalized LFQ intensity values were imputed by drawing random values from a normal distribution after filtering out contaminants, proteins with less than 2 peptides and less than 2 quantified values in at least one group. Differences between groups were statistically evaluated using the LIMMA 3.52.1^66^ at 5 % FDR (Benjamini-Hochberg). 21 samples were analyzed in total (three biological replicates per condition, one technical replicate per biological replicate). The samples from HEK FITR cells not expressing any construct were used as control for unspecific protein binding.

### KO cell line generation

For each targeted gene, we selected a pair of gRNA sequences that cut both in the same and one of the first exons. The distances between the cut sites were chosen so that they would cause a frameshift if repaired without the cut-out fragment and without any additional indels. We cloned the two gRNA sequences into two different versions of the pX458 vector^67^, one co-expressing mCherry, the other co-expressing GFP, as described by ref. ^67^. Cells were co-transfected with the two sgRNA/Cas9 vectors. Two days after the transfection, the cells were sorted by FACS (BD FACSMelody) for both GFP and mCherry expression. The mCherry- and GFP-positive cells were expanded for 5 days and then single-cell sorted for negative mCherry and GFP expression. The clonal cell lines were expanded for two weeks and then genotyped by PCR. Clones that showed the intended homozygous deletion were subsequently validated by Sanger sequencing to ensure that the wanted frameshift was in place. The KO clones were finally validated by western blotting. As validation of the FAM98C KO was not possible by western blotting, we validated the KO by mass spectrometry. Used gRNA sequences are listed in Supplementary Table 1.

### FAM98C KO validation by MS

HEK FITR WT and HEK FITR FAM98C KO cells were grown to 90 % confluence in a 6-well plate. The medium was removed and the cells were carefully washed once with 1 mL ice-cold PBS. The cells were resuspended by adding ice-cold PBS and pipetting several times up and down. The cells were pelleted at 1000 × *g*, 4 °C for 1 min. The supernatant was completely removed and the pellets were lysed in 100 µL 2 % sodium deoxycholate (SDC) and 100 mM Tris-HCl pH 8.8 at 80 °C for 10 min. After cooling down, 1 µL Benzonase was added and kept on ice for 15 min. Then the samples were sonicated using a Bioruptor at high energy for 5 cycles 30/30. After centrifugating at RT 15,000 × *g* for 10 min, the supernatant was transferred to a new tube. 4 times samples volume of cold (–20 °C) 100 % acetone was added to the tube. After thoroughly mixing, the precipitation was done at 4 °C overnight. After centrifugation at 10 °C 15,000 × *g* for 10 min, the supernatant was removed. The pellets were washed with 500 µL cold (–20 °C) 80 % acetone. After centrifugation at 10 °C 15,000 × *g* for 10 min, the supernatant was removed. After completely removing acetone, the pellets were air dried in hood for 5 min. The protein pellets were dissolved in 100 µL 2 % SDC in 100 mM Tris-HCl pH 8.8, at 23 °C shaking 800 rpm for 1 h. Then the samples were sonicated using a Bioruptor at high energy for 3 cycles 30/30. The pellets were disrupted by vortexing and mixing and then incubated at 23 °C shaking 800 rpm for 1 h. After centrifugating at 10 °C 15,000 × *g* for 10 min, the supernatant was transferred to a new tube and the protein concentration was determined with a micro BCA assay (Thermo) following the vendor’s protocol. 50 µg protein in 15 µL were reduced by adding 250 mM DTT to final concentration of 10 mM for 30 min at 50 °C and alkylated by adding 500 mM IAA to final concentration of 20 mM and incubating for 30 min at RT in the dark. The remaining IAA was quenched for 10 min by adding 5 mM DTT. The samples were diluted to 1 % SDC with 15 µL 100 mM Tris-HCl pH 8.8. Proteins were digested by adding 500 ng LysC (MS grade, FUJIFILM Wako chemicals) at RT for 3 h. Then, 1 µg trypsin (Trypsin Gold, Promega; 1 µg/μL solution in 1 mM HCl) was added and incubated at 37 °C overnight. The digest was stopped by the addition of 10 % TFA to reach a final concentration of 2 %. SDC was pelleted by centrifugation at 18,500 g for 10 min. The resulting supernatant was desalted using C18 Stagetips^64^. LC-MS analysis was performed on a Vanquish Neo UHPLC system (Thermo Scientific) coupled to an Orbitrap Exploris 480 mass spectrometer (Thermo Scientific). The system was equipped with a FAIMS Pro interface (Thermo Scientific), a Nanospray Flex ion source (Thermo Scientific), coated emitter tips (PepSep, MSWil), and a Butterfly Portfolio Heater (Phoenix S&T). Peptides were loaded onto a trap column (PepMap Neo C18 5 mm × 300 µm, 5 μm particle size, Thermo Scientific) using 0.1 % TFA as mobile phase, and separated on an analytical column (Acclaim PepMap 100 C18 HPLC Column, 50 cm × 75 µm, 2 μm particle size, Thermo Scientific), applying a linear gradient starting with a mobile phase of 98 % solvent A (0.1% FA) and 2% solvent B (80 % acetonitrile, 0.08% FA), increasing to 35% solvent B over 30 min at a flow rate of 230 nL/min. The analytical column was heated to 30 °C. The mass spectrometer was operated in DDA mode. Survey scans were acquired from 350 to 1500 m/z at CV -42, normalized AGC target of 100%, resolution of 60,000. The PRM parameters - precursor m/z and retention time–were built based on a test run. Precursor of peptides of interest were isolated in a 0.7 m/z window and fragmented with 30% HCD collision energy. Orbitrap resolution was set to 30,000, the normalized AGC target to 100%. Maximal injection time for target peptides was set to 300 ms. PRM measurements were analysed in Skyline (24.1.1.284^68^,). After loading the list of the 4 PRM targets, the raw data were imported. All peptides and their transitions were validated manually based on retention time, relative ion intensities, and mass accuracy. Three of the most intense non-interfering transitions of the target peptides were selected and their peak areas were exported from Skyline. The summed peak areas were used for peptide quantification. 8 samples were analyzed in total (three biological replicates per condition as well as two blank controls).

### Endogenous mNeonGreen-tagging

We selected gRNA sequences that cut in the region surrounding the start codon of the targeted gene and cloned them into the pX330-U6-Chimeric_BB-CBh-hSpCas9-hGem(1/110)-P2A-mCherry vector (modified from^69^) as described by ref. ^67^. Repair templates were cloned so that they have the following structure: LeftHomologyArm-mNeonGreen-linker-RightHomologyArm. The homology arms contained the sequences surrounding the gRNA cut site (~500 bp each), with the 3’ end of the LeftHomologyArm ending just after the start codon of the endogenous locus and the 5’ end of the RightHomologyArm beginning with the first codon after the start codon of the endogenous locus. Cells were co-transfected with the corresponding repair template and the sgRNA/Cas9 vector. Two days after the transfection, the cells were sorted by FACS (BD FACSMelody) for mCherry expression. The mCherry-positive cells were expanded for 5 days and then single-cell sorted for positive mNeonGreen expression and negative mCherry expression. The clonal cell lines were expanded for two weeks and then genotyped by PCR. Clones that had the intended homozygous knock-in were subsequently validated by Sanger sequencing of the insertion locus and by western blotting. Used gRNA sequences are listed in Supplementary Table 1.

### c-MYC-NLS-tagging of endogenous RTCB

For adding the c-MYC NLS onto endogenous RTCB, we used the same sgRNA/Cas9 vector that we used for the mNeonGreen-tagging on RTCB. As repair template, we used an ssDNA oligo containing the nucleotide sequence of the c-MYC NLS flanked by left and right homology arms (50 bp each) that contained the sequences surrounding the gRNA cut site. The 3’ end of the left homology arm ended just after the start codon of the endogenous locus and the 5’ end of the right homology arm began with the first codon after the start codon of the endogenous locus. Cells were co-transfected with the repair template and the sgRNA/Cas9 vector. Two days after the transfection, the cells were sorted by FACS (BD FACSMelody) for mCherry expression. The mCherry-positive cells were expanded for 5 days and then single-cell sorted for negative mCherry expression. The clonal cell lines were expanded for two weeks and then genotyped by PCR. Clones that had the intended homozygous knock-in were subsequently validated by Sanger sequencing of the insertion locus and by western blotting. Used gRNA sequences are listed in Supplementary Table 1.

### Northern blotting

Total RNA was isolated from cells using TRIzol (Thermo, 15596018), following the manufacturer’s instructions. The RNA samples were mixed with an equal volume of 2x FA buffer (90 % Formamide, 50 mM EDTA pH 8.0, 0.025 % Bromophenol blue w/v, 0.025 % Xylene cyanol w/v), boiled at 95 °C for 3 min and then separated by 10 % UREA-PAGE (National Diagnostics, EC-833), using 0.5x TBE as running buffer (65 mM Tris pH 7.6, 22.5 mM boric acid, 1.25 mM EDTA). The gels were then blotted onto Amersham Hybond-N+ nylon membranes (Cytiva, RPN303B) at a constant current of 250 mA for 3 h, using 0.5x TBE as transfer buffer. After the transfer, the RNA was UV-crosslinked to the membrane and the membrane was blocked in 30 mL pre-heated hybridization buffer [20 mM sodium phosphate pH 7.2, 75 mM sodium citrate, 750 mM NaCl, 7 % SDS w/v, 3 mg sonicated salmon sperm DNA (Thermo, AM9680)] for 1 h, rotating at 50 °C. Radiolabelled DNA probes were added to the hybridization buffer and the membranes were incubated ON rotating at 50 °C. The hybridization solution was discarded and the membranes were washed twice for 1 min in pre-heated (50 °C) low stringency wash buffer (75 mM sodium citrate pH 7.0, 750 mM NaCl, 5% SDS w/v) and then for 1 min in pre-heated (50 °C) high stringency wash buffer (15 mM sodium citrate pH 7.0, 150 mM NaCl, 1% SDS w/v). For visualization of the radioactive signal, phosphor screens were exposed to the northern blots and subsequently imaged using an Amersham Typhoon biomolecular imager. For quantification, we used Fiji 2.16.0^63^ to measure the intensities of the imaged bands. Within an experimental replicate, the intensities were first normalized to the loading control (snRNA U6) and then further normalized to the control sample (WT). Statistical analysis was performed using GraphPad Prism 10.5.0. Used DNA probes are listed in Supplementary Table 2.

### RT-qPCR

cDNA was generated from total RNA using the Maxima First Strand cDNA Synthesis Kit (Thermo, K1672), following the manufacturer’s instructions, and was diluted 1:10 in nuclease-free water before downstream analysis. For quantitative measurement of mRNA levels using RT-qPCR, a master mix was prepared for each set of primers: per reaction 5 µL of 2x GoTaq (Promega, A600A), 0.8 µL primer master mix (5 µM forward primer, 5 µM reverse primer) and 2.2 µL nuclease-free water were mixed. A single reaction, in a well of a 384-well plate, consisted of 2 µL diluted cDNA and 8 µL reaction master mix. The RT-qPCR was performed in a CFX384 Touch thermocycler (Bio-Rad) using the following protocol: 95 °C for 5 min, 95 °C for 10 s, 60 °C for 30 s, plate read, repeat previous 3 steps for 60 cycles, 95 °C for 10 s, melt curve determination from 55  to 95 °C in 0.5 °C increments. The quality of the PCR products was evaluated by melting curve analysis. Relative expression levels were calculated from Ct values with the 2^-ΔΔCt^ method, normalizing expression levels to *ACTB* mRNA levels^70^. Statistical analysis was performed using GraphPad Prism 10.5.0. Used primer sequences are listed in Supplementary Table 2.

### Immunofluorescence and confocal microscopy

UV-sterilized, quadratic, 12 mm #1.5 coverslips were transferred into a 12-well plate, coated with poly-L-lysine (Sigma, P6282) and seeded with 50–100 k HEK cells. Optionally, constructs for imaging were either transiently transfected or, if using stable cell lines, induced by addition of doxycycline (1 μg/mL). Transiently transfected cells were further processed 48 h after the transfection. Cells that were treated with doxycycline were further processed 24 h after doxycycline addition. The medium was removed and the cells were carefully washed twice with 500 µL PBS. For fixation, 500 µL 4 % paraformaldehyde in PBS was added per well and incubated 15 min at RT. The solution was removed and the cells were washed once with 500 µL PBS. For permeabilization, 500 µL 0.3 % Triton X-100 in PBS was added per well and incubated 5 min at RT. The solution was removed and the cells were washed once with 500 µL PBS. The coverslips were blocked with 50 µL blocking solution (5% normal goat serum in PBS) for 45 min at RT in the dark. The blocking solution was removed and 30 µL primary antibody diluted in blocking solution was added and incubated for 1 h at RT in the dark. The antibody solution was removed and the coverslips were washed twice with 100 µL PBS. 60 µL secondary antibody diluted 1:1000 in blocking solution was added and incubated for 45 min at RT in the dark. The solution was removed and the coverslips were washed once with 100 µL PBS. 80 µL of 0.5 µg/mL 4’,6-diamidino-2-phenylindole (DAPI) in PBS was added and incubated 10 min at RT in the dark. The solution was removed and the coverslips were washed twice with 100 µL PBS. Finally, the coverslips were mounted onto glass slides with VECTASHIELD PLUS mounting media (Vector Laboratories, H-1900) and sealed with clear nail polish. The slides were stored at 4 °C in the dark until imaging. Images were acquired using an LSM 980 Airyscan 2 confocal microscope (Zeiss). For all samples that belonged to the same experiment, the same laser power settings as well as photomultiplier voltages were used. Used primary antibodies: anti-FLAG (1:200; Sigma Aldrich, F1804). Used secondary antibodies: anti-mouse Alexa Fluor Plus 647 (1:1000; Invitrogen, A32728).

### Quantification of nuclear-cytoplasmic tRNA-LC distribution from microscopy images

Cells were identified and segmented from microscopy images using CellProfiler 4.2.8^71^. First, nuclei were identified from the DAPI signal. Then, using the identified nuclei as seeds, cell boundaries were identified from the mNeonGreen signal. Based on the identified nuclear area, each cell was segmented into nucleus and cytoplasm. For each identified and segmented cell, the mean intensity of the mNeonGreen signal in the cytoplasm and nucleus was calculated. As measure for nuclear-cytoplasmic distribution of the tRNA-LC, we calculated the logarithm of the ratio between mean nuclear and mean cytoplasmic mNeonGreen signal. As a final step, as the automated segmentation is not perfect, we manually curated the identified cells. We excluded cells that were undergoing cell division (condensed chromatin), had wrongly defined boundaries (either for the cell or the nucleus) or had more than one nucleus (multiple cells identified as a single one). Furthermore, when quantifying cells expressing a FLAG-tagged construct, we excluded cells that did not express the construct (very low mean anti-FLAG signal, similar to cells not expressing a FLAG-tagged protein; the cut-off value was the same for all cells within an experiment). Statistical analysis was performed using GraphPad Prism 10.5.0.

### Monoclonal anti-ASW antibody generation

Mouse monoclonal anti-ASW antibody, clone 1C6-H6, was generated against full-length recombinant human ASW (UniProt: Q9BVC5-1). Briefly, BALB/c mice were 4x immunized with 50 µg of Ashwin antigen per immunization. 4 days after the final boost, splenocytes were fused with X63.Ag8-653 mouse myeloma cells by addition of Hybri-Max polyethylen glycol (Sigma). Hybridoma clones were grown under HAT selection for 8 days and supernatants were screened for the presence of ASW-specific antibodies by western blot with cell lysate of HEK FITR cells over-expressing FLAG-HA-tagged ASW as well as with HEK FITR WT versus ASW KO cell lysates. Single clone 1C6-H6 was obtained by minimal dilution of mix clone 1C6 under HAT selective conditions, and further characterized for ASW-specificity as described above.

### Reproducibility

All experiments were independently performed multiple times and yielded similar results. All experiments were performed three times, with the exception of the experiments displayed in figures: 1 F (two replicates), 2 A (two replicates), 2B (four replicates), 2 C (five replicates), 4B (two replicates), 4 C (two replicates), 6B (two replicates), S4B (two replicates), S5 (two replicates), S6B (six replicates), S6D (five replicates), S7B (two replicates), S7C (six replicates), S11A (two replicates) and S11B (two replicates). Western blots meant to confirm successful genome editing rather than generating experimental data were not replicated (Fig. 7A and Supplementary Fig. 8).

### Reporting summary

Further information on research design is available in the Nature Portfolio Reporting Summary linked to this article.

## Supplementary information

Supplementary Information

Reporting Summary

Transparent Peer Review file

## Source data

Source data

## Data Availability

Raw mass spectrometry data generated in this study have been deposited to the ProteomeXchange Consortium via the PRIDE partner repository with the dataset identifiers PXD076703 (co-IP/MS of the human FAM98 proteins) and PXD076694 (FAM98C KO validation). Other data that support the findings of this study are available within the paper and its supplementary information. Source data are provided with this paper.
