# Supplementary material for: Ashwin and FAM98 paralogs define nuclear and cytoplasmic RNA ligase complexes for tRNA biogenesis
Source: Nat Commun. 2026 Sep 17;17:9882. doi: 10.1038/s41467-026-77451-x (PMC13586215; doi:10.1038/s41467-026-77451-x)
Supplement: Supplementary file 1 [file 41467_2026_77451_MOESM1_ESM.pdf]

# Supplementary Figures

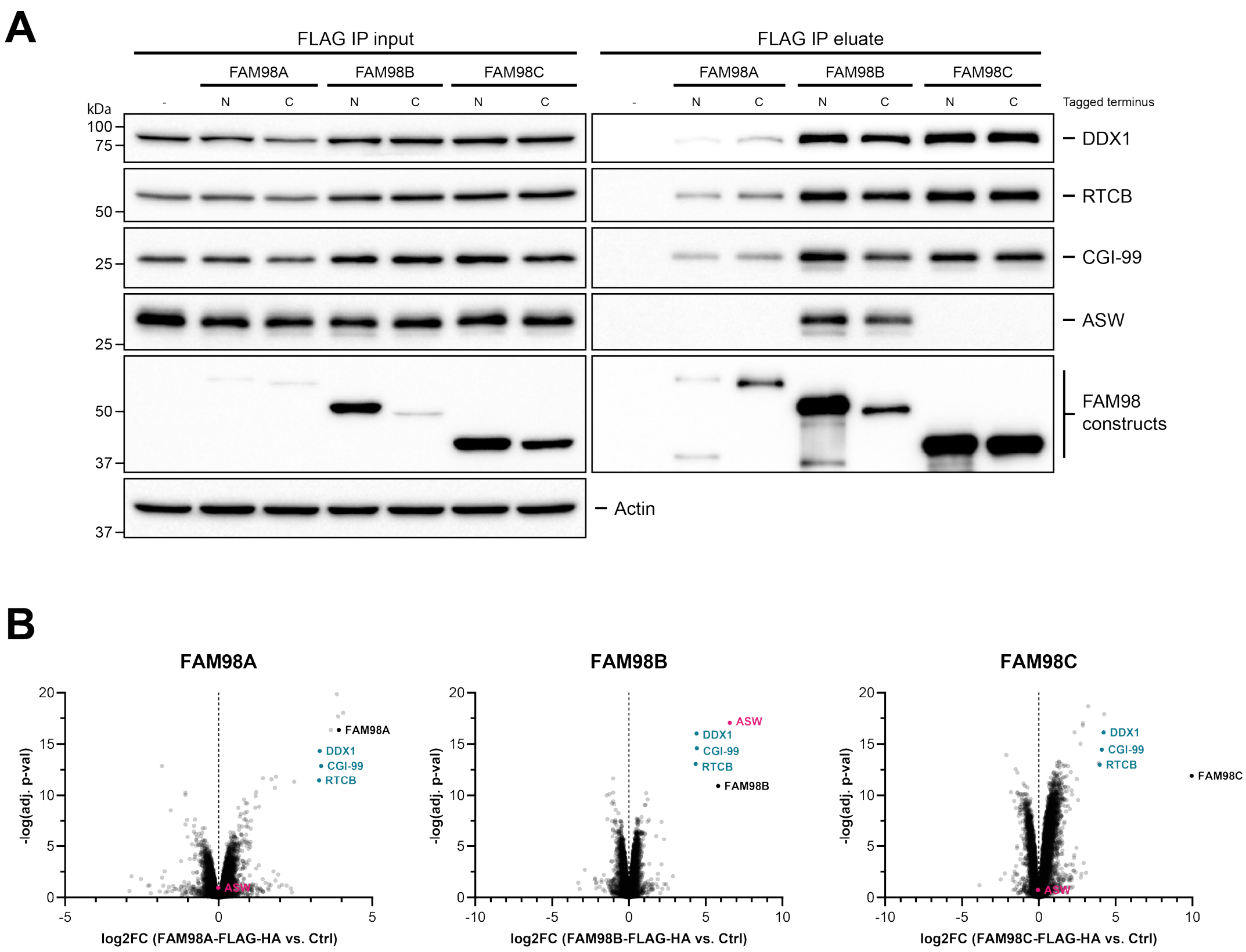


**Supplementary Figure 1: Only FAM98B-containing tRNA-LC can bind ASW.** (**A**) HEK FITR cell lines stably carrying doxycycline-inducible expression constructs for either N- or C-terminally FLAG-HA-tagged FAM98A, FAM98B or FAM98C were treated with doxycycline for 24 h. As control, HEK FITR cells without any integrated construct (-) were used. The expressed constructs were isolated using an anti-FLAG IP. Input and eluate samples of the IP were analyzed by WB for DDX1, RTCB, CGI-99, ASW, FLAG and ACTA1 (evaluation of equal loading). Equal amounts of total protein (input) or volume (eluate) were loaded per lane. (**B**) Anti-FLAG IP as in **A**, but with HEK FITR cells expressing C-terminally FLAG-HA-tagged FAM98A, FAM98B or FAM98C. After binding and washing, the beads were subjected to tandem mass spectrometry to identify and quantify bound proteins. The displayed protein enrichments are the results from three independent experiments. Statistical significance was calculated by performing two-sided t-tests and the resulting p values were adjusted with the Benjamini-Hochberg procedure. Source data are provided as a Source Data file.

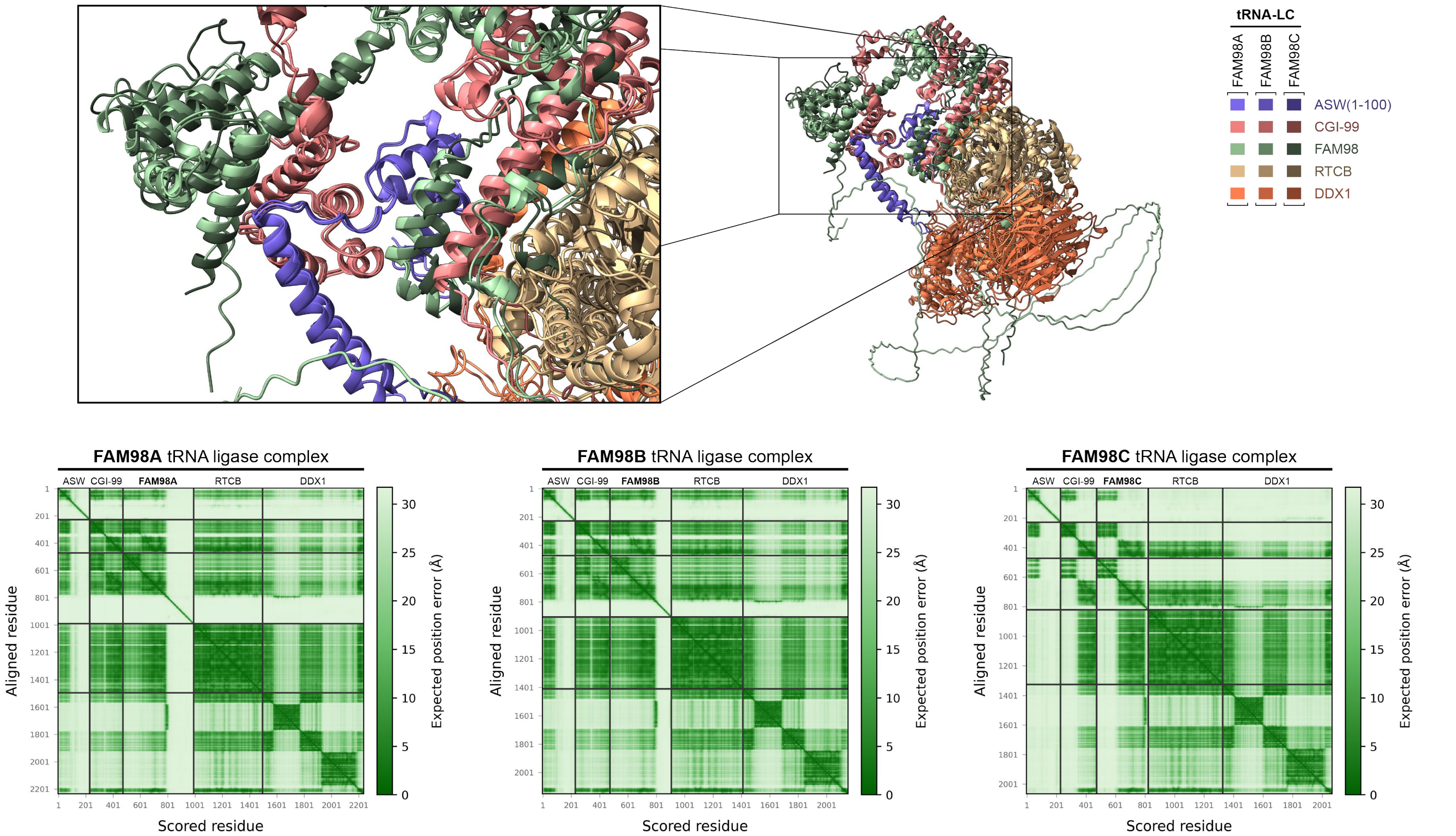


**Supplementary Figure 2: AlphaFold 3 cannot to predict the inability of FAM98A- and FAM98C-containing tRNA-LC to bind ASW.** AlphaFold 3 predictions of different tRNA ligase complexes, containing ASW, CGI-99, RTCB and DDX1 as well as either FAM98A, FAM98B or FAM98C. For the overlay, the predicted structures were aligned based on the positioning of ASW. Sequences used for the prediction (UniProt): Q9BVC5-1 (ASW), Q9Y224 (CGI-99), Q9Y3I0 (RTCB), Q92499-1 (DDX1), Q8NCA5 (FAM98A), Q52LJ0-2 (FAM98B) and Q17RN3-1 (FAM98C).

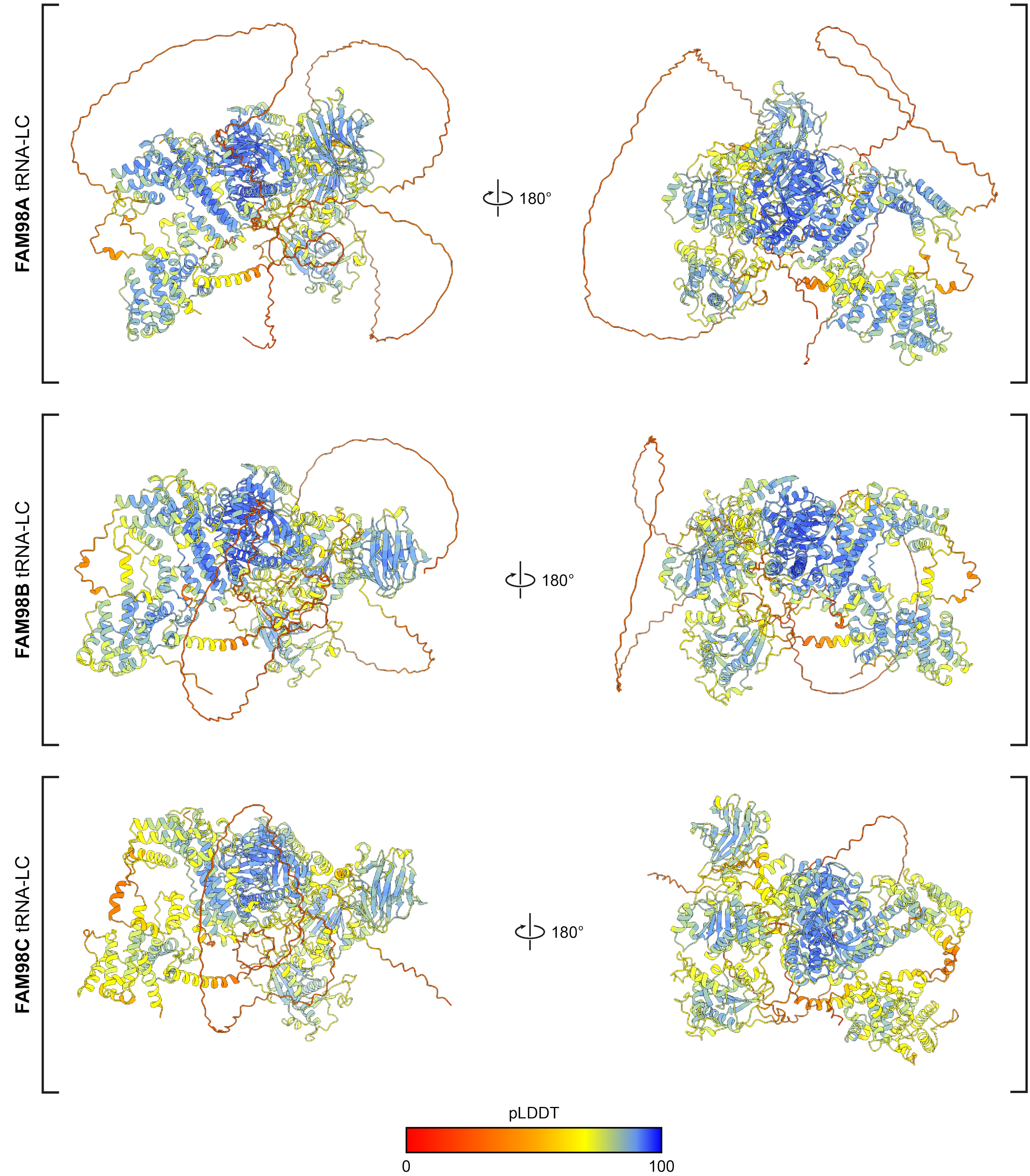


**Supplementary Figure 3: pLDDT score overlays of the tRNA-LC AlphaFold predictions.** The three different tRNA-LC AlphaFold models, that are overlayed in Supplementary Figure 2, with residues colored based on their individual pLDDT scores. Two different viewing angles are shown per model.

**A**

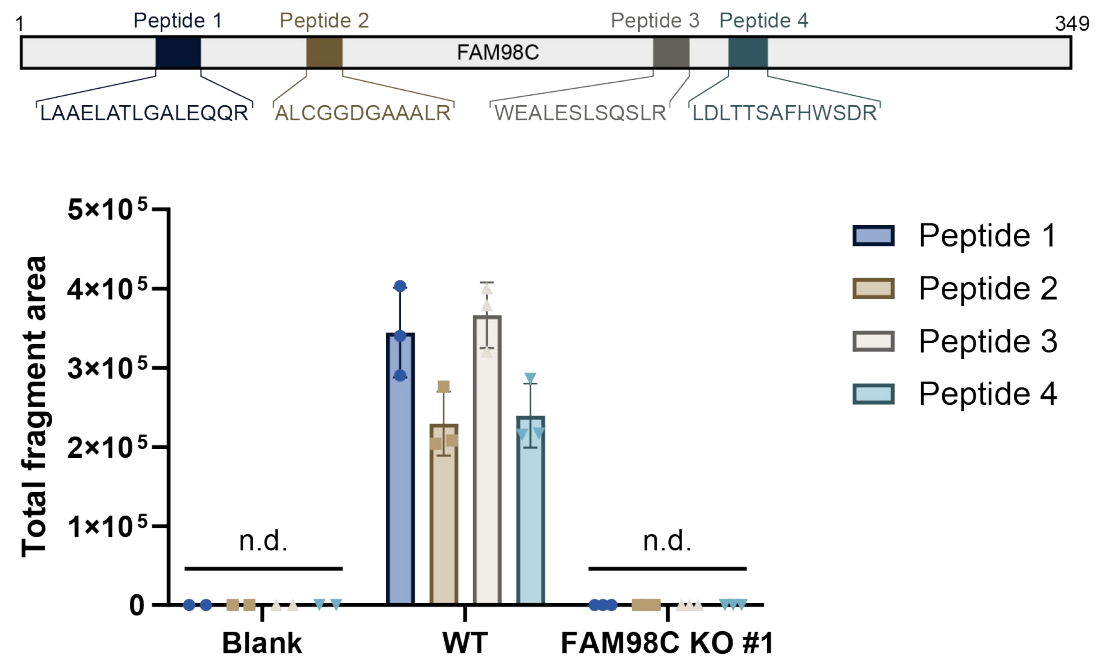



**B**

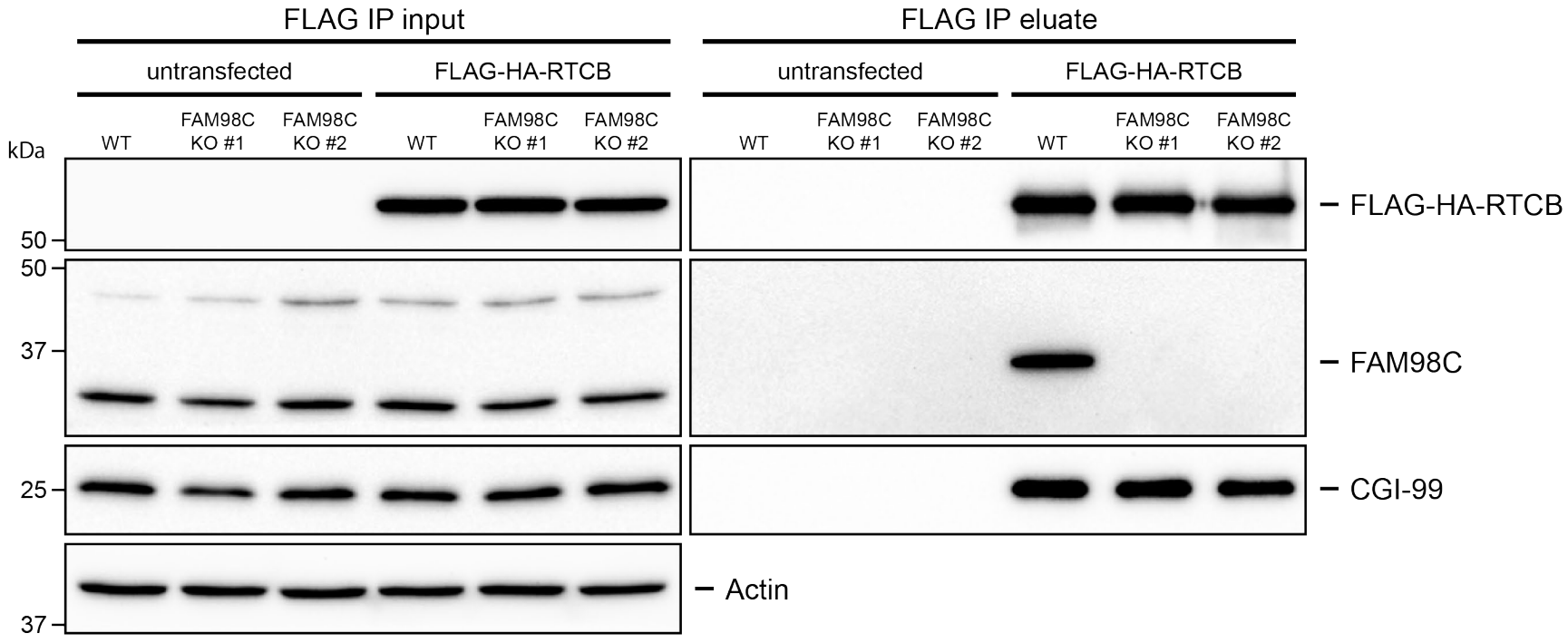



**Supplementary Figure 4: Validation of the FAM98C KO cell lines.** (**A**) The presence of four FAM98C-specific peptides was determined by tandem mass spectrometry in cell lysates of HEK FITR WT and HEK FITR FAM98C KO (clone 1) cells. Lysis buffer was used as negative control (blank). Summarized data from two (blank) and three (lysates) independent experiments. (**B**) HEK FITR WT and HEK FITR FAM98C KO cells were transiently transfected with an expression construct coding for FLAG-HA-RTCB. As control, cells without any transfected construct were used. The expressed constructs were isolated using an anti-FLAG IP. Input and eluate samples of the IP were analyzed by WB for FLAG, FAM98C, CGI-99 and ACTA1 (evaluation of equal loading), to validate the KO of FAM98C. Equal amounts of total protein (input) or volume (eluate) were loaded per lane. Source data are provided as a Source Data file.

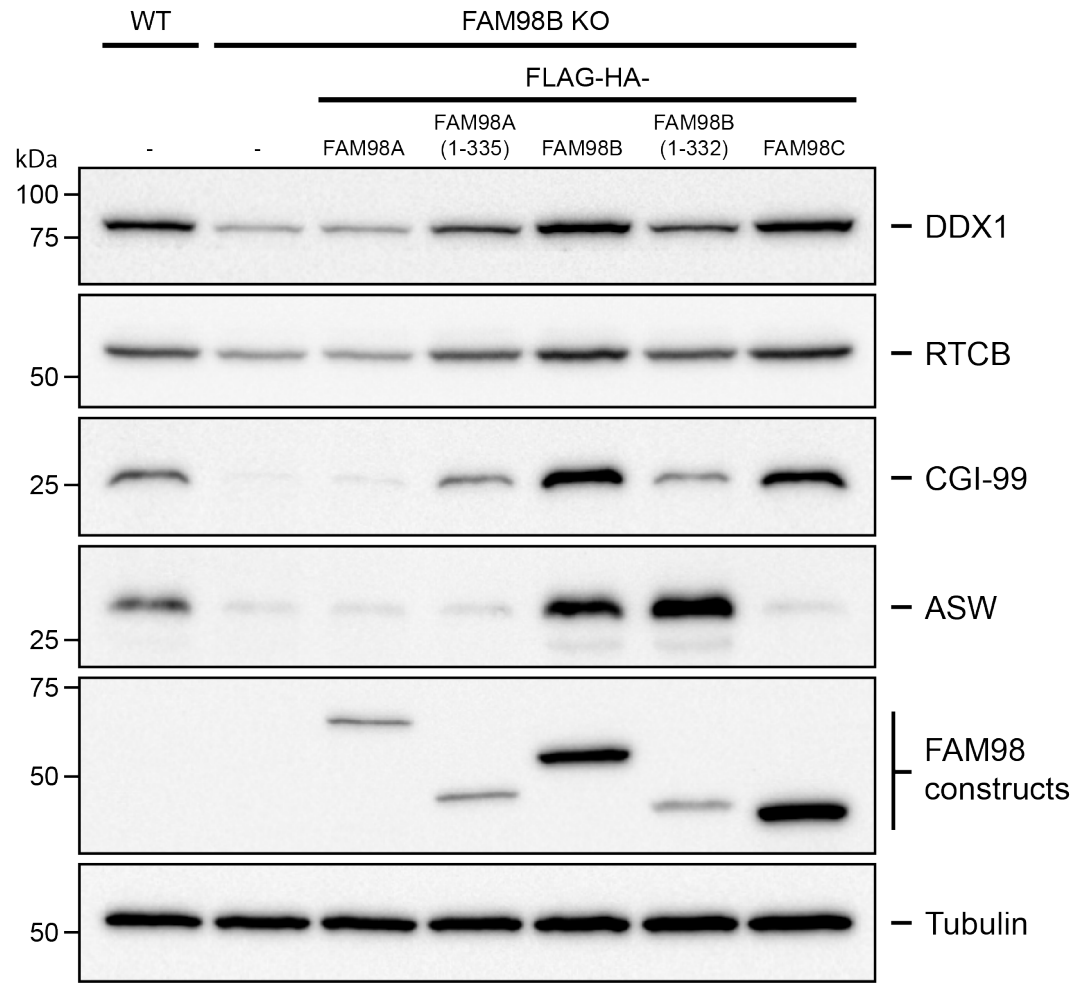


**Supplementary Figure 5: Expression of FAM98A, FAM98B or FAM98C restores the levels of the other tRNA-LC subunits to different extents in FAM98B KO cells.** HEK FITR FAM98B KO cell lines stably carrying doxycycline-inducible expression constructs for FLAG-HA-tagged FAM98A, FAM98A(1-335), FAM98B, FAM98B(1-332) or FAM98C were analyzed by WB for levels of tRNA-LC subunits, FLAG (construct expression) as well as Tubulin (evaluation of equal loading). As control, HEK FITR WT and HEK FITR FAM98B KO cells without any integrated construct (-) were used. All cell lines were treated with doxycycline for 48 h prior to lysate preparation. Source data are provided as a Source Data file.

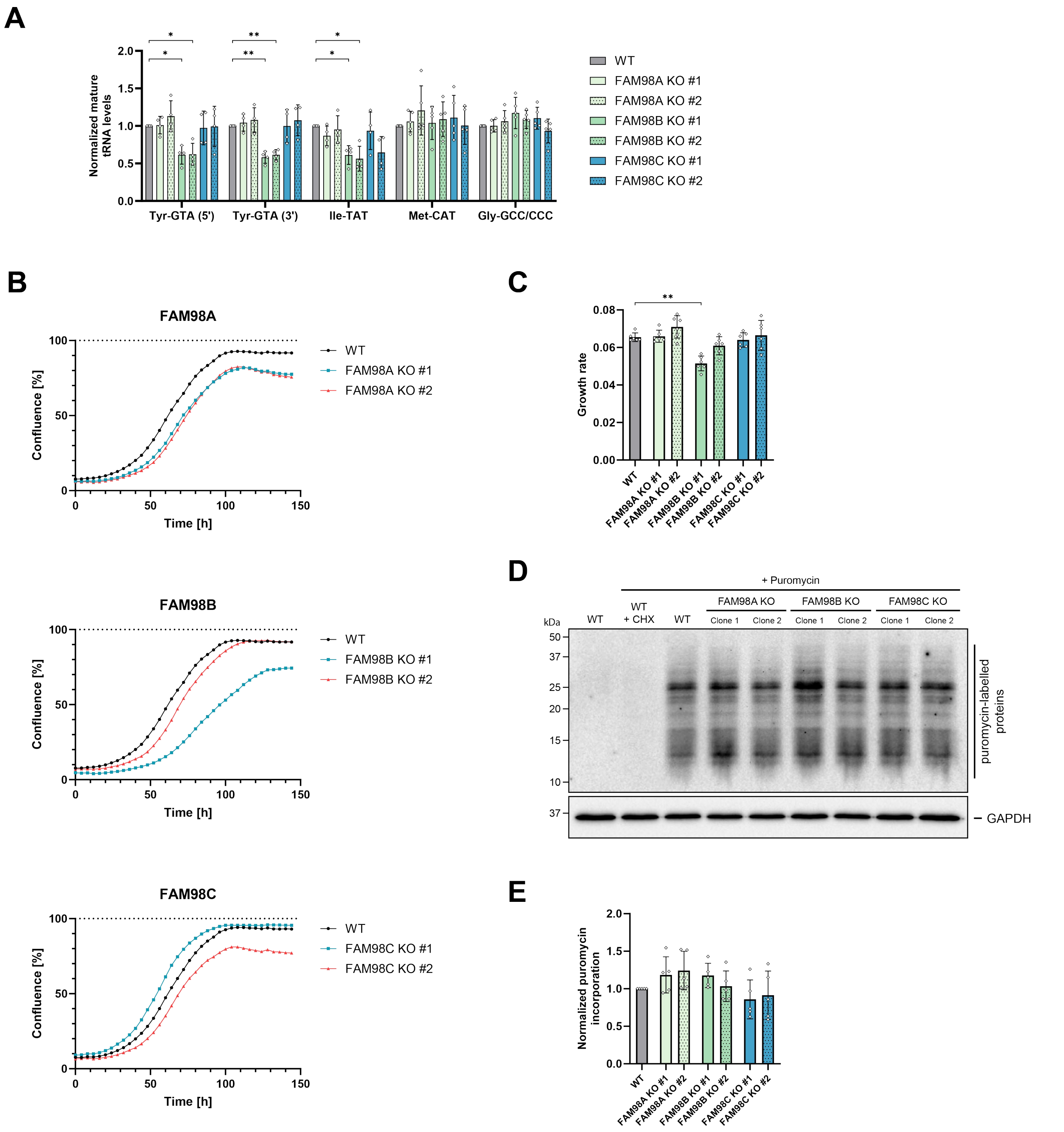


**Supplementary Figure 6: Characterization of the FAM98 KO cell lines.** (**A**) Quantification of **Fig. 2B, C**. Mature tRNA levels were normalized to the levels of snRNA U6 and then to the WT sample. Values represent means ± SD from 4 (Tyr and Ile) or 5 (Gly and Met) independent experiments. From left to right: * P = 0.0275, * P = 0.0434, ** P = 0.0063, ** P = 0.0049, * P = 0.0266, * P = 0.041 (two-way ANOVA, Dunnett's test). (**B**) Growth curves of HEK FITR WT, FAM98A KO, FAM98B KO and FAM98C KO cells were acquired with an Incucyte live-cell analysis system, taking images every 4 h. Two clones were analyzed for each KO cell line. (**C**) Growth rates of WT cells and FAM98 KO cell lines, derived from logistic curves that were fitted to the growth data of **B**. Data represents means ± SD from 6 independent experiments. ** P = 0.0015 (one-way ANOVA, Dunnett's test). (**D**) Global protein synthesis in HEK FITR WT, FAM98A KO, FAM98B KO and FAM98C KO cells was evaluated by WB for puromycin-labelled proteins after the cells have been treated with 10 µg/mL puromycin for 10 min. As control, HEK FITR WT cells without puromycin addition or cycloheximide-pre-treated (10 µg/mL for 90 min prior to puromycin addition) WT cells were used. WBs against GAPDH were used as evaluation of equal loading. Two clones were analyzed for each KO cell line. Equal amounts of total protein were loaded per lane. (**E**) Quantification of **D**. Puromycin-incorporation was normalized to GAPDH levels and then to the WT sample. Data represents means ± SD from 5 independent experiments. Source data are provided as a Source Data file.

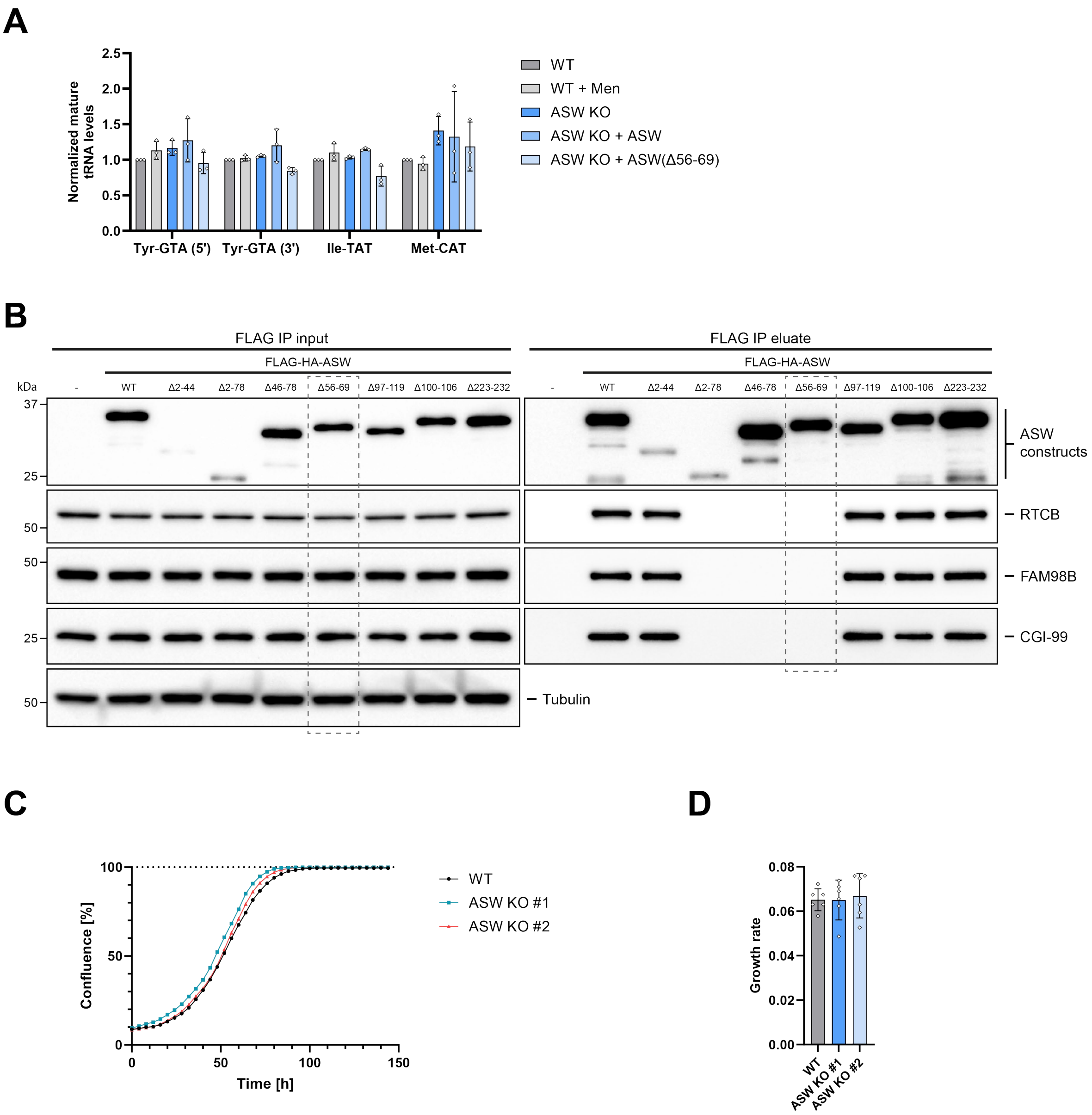


**Supplementary Figure 7: Characterization of the ASW KO cell lines and validation of ASW tRNA-LC interface mutants.** (**A**) Quantification of **Fig. 3B**. Mature tRNA levels were normalized to the levels of snRNA U6 and then to the WT sample. Values represent means ± SD from three independent experiments. (**B**) HEK FITR WT cells were transiently transfected with different FLAG-HA-ASW expression constructs (WT and truncation mutants). As control, cells without any transfected construct (-) were used. The expressed constructs were isolated using an anti-FLAG IP. Input and eluate samples of the IP were analyzed by WB for FLAG, RTCB, FAM98B, CGI-99 and Tubulin (evaluation of equal loading), to identify mutants that do not bind the tRNA-LC. Equal amounts of total protein (input) or volume (eluate) were loaded per lane. (**C**) Growth curves of HEK FITR WT and ASW KO cells (2 clones) were acquired with an Incucyte live-cell analysis system, taking images every 4 h. (**D**) Growth rates of WT cells and the ASW KO cell lines, derived from logistic curves that were fitted to the growth data of **C**. Data represents means ± SD from 6 independent experiments. Source data are provided as a Source Data file.

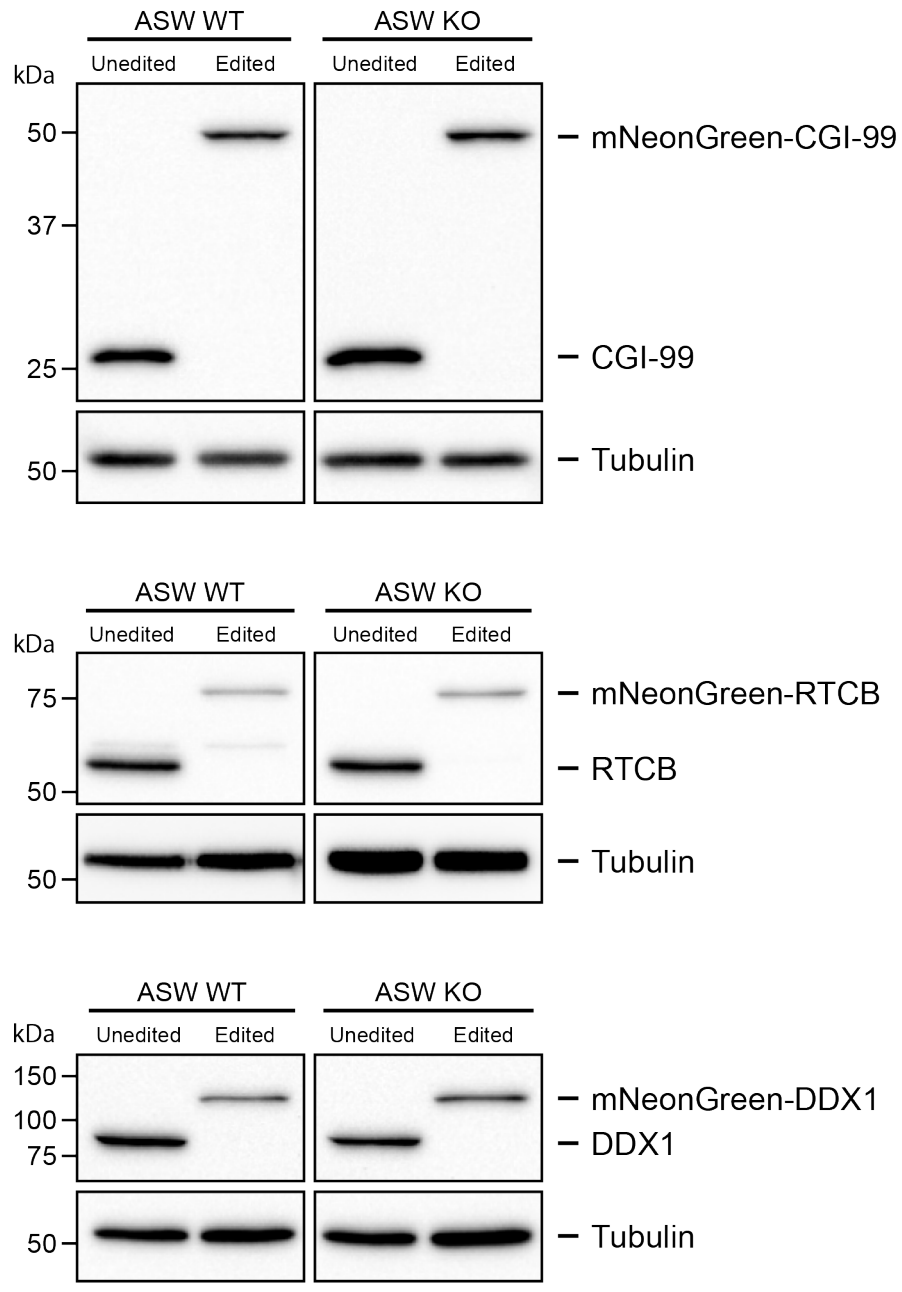


**Supplementary Figure 8: Validation of the mNeonGreen knock-ins on endogenous CGI-99, RTCB and DDX1.** Whole cell lysates of HEK FITR WT and ASW KO cells endogenously expressing mNeonGreen-RTCB, mNeonGreen-CGI-99 or mNeonGreen-DDX1 were evaluated by WB for levels of the edited protein as well as Tubulin. Equal amounts of total protein were loaded per lane. Source data are provided as a Source Data file.

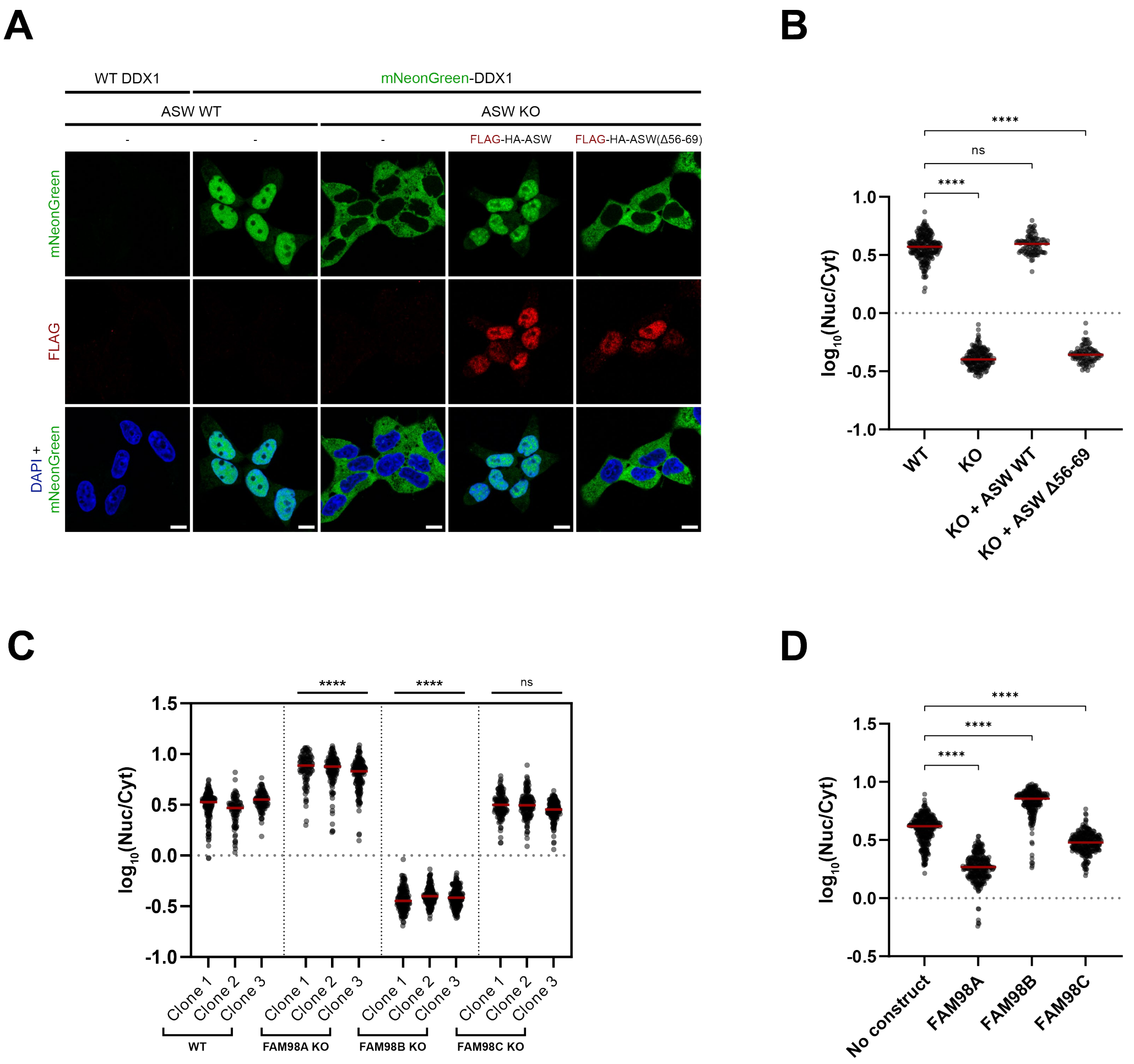


**Supplementary Figure 9: ASW and the FAM98 proteins define the subcellular localization of the tRNA-LC.** (**A**) HEK FITR ASW KO cells endogenously expressing mNeonGreen-DDX1 and stably carrying doxycycline-inducible expression constructs for different FLAG-HA-tagged ASW constructs (WT and Δ56-69) were treated with doxycycline for 24 h. Anti-FLAG immunofluorescence was performed and cells were stained with DAPI, to determine the localization of endogenous tRNA-LC as well as the expressed constructs by microscopy. As control, HEK FITR WT and ASW KO cells endogenously expressing mNeonGreen-DDX1 without any integrated construct (-) were used. Scale bars indicate 10 µm. (**B**) Quantification of **A**. The logarithm of the ratio between the mean nuclear and mean cytoplasmic signal of mNeonGreen-DDX1 was used to determine the nuclear-cytoplasmic distribution of the tRNA-LC. Red lines indicate the mean. Each dot represents a single quantified cell of the indicated sample. Number of quantified cells: 214 (WT), 164 (KO), 92 (KO + ASW), 89 (KO + ASW Δ56-69). Indicated P values: ns P = 0.1391, **** P < 0.0001 (one-way ANOVA, Dunnett's test). (**C**) Quantification of **Fig. 4B**, done as in **B**. Red lines indicate the mean. Each dot represents a single quantified cell of the indicated sample. Number of quantified cells: 155 (WT clone 1), 89 (WT clone 2), 104 (WT clone 3), 101 (FAM98A KO clone 1), 130 (FAM98A KO clone 2), 165 (FAM98A KO clone 3), 149 (FAM98B KO clone 1), 187 (FAM98B KO clone 2), 178 (FAM98B KO clone 3), 125 (FAM98C KO clone 1), 147 (FAM98C KO clone 2), 171 (FAM98C KO clone 3). Indicated statistical significance is relative to WT. Indicated P values: ns P = 0.9491, **** P < 0.0001 (nested one-way ANOVA, Dunnett's test). (**D**) Quantification of **Fig. 4C**, done as in **B**. Red lines indicate the mean. Each dot represents a single quantified cell of the indicated sample. Number of quantified cells: 317 (no construct), 267 (FAM98A), 232 (FAM98B), 254 (FAM98C). All indicated P values (****) < 0.0001 (one-way ANOVA, Dunnett's test). Source data are provided as a Source Data file.

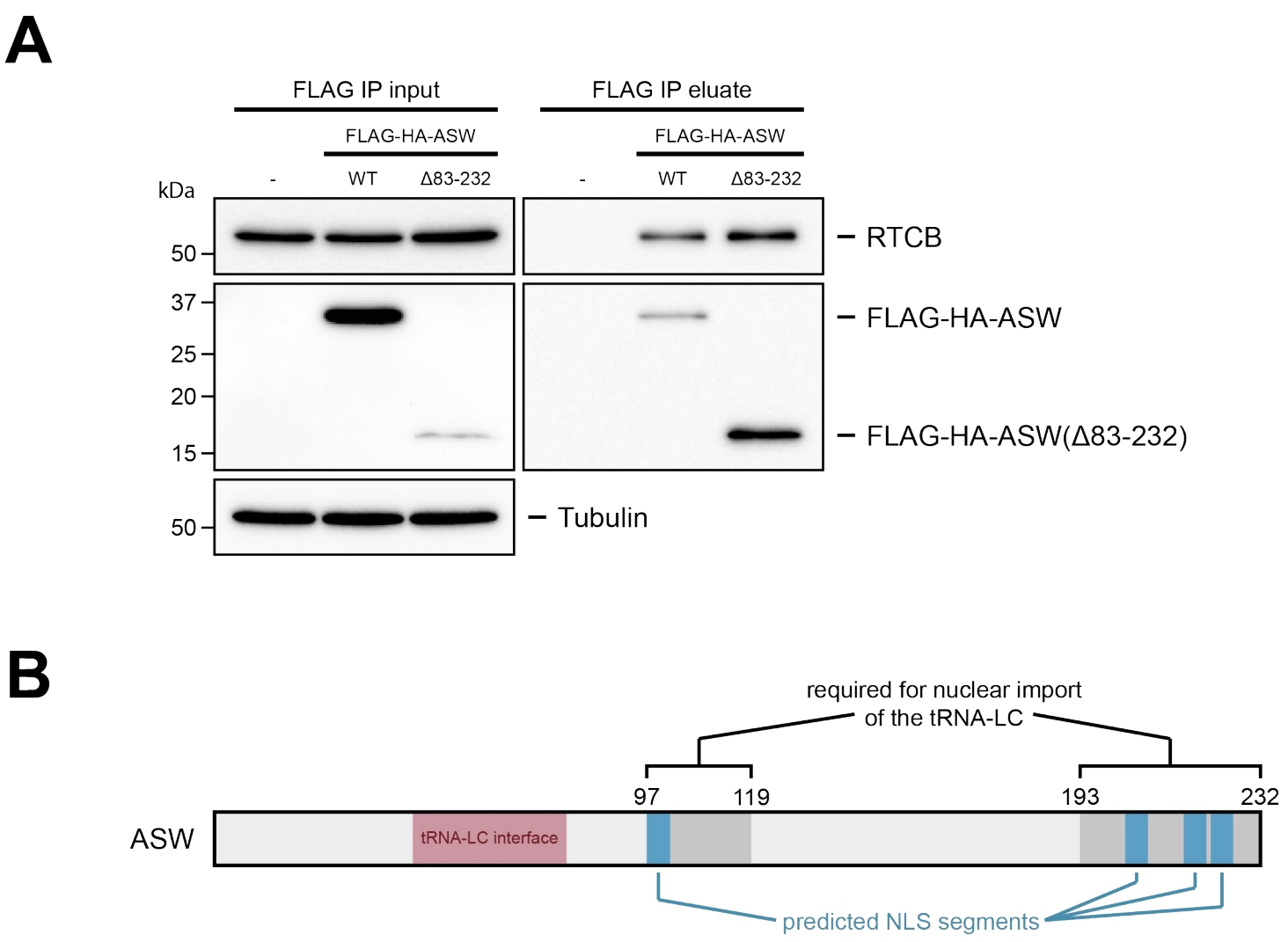


| Position | Recommendation score | Entropy score | Recommended NLS segment |
|---|---|---|---|
| 216-220 | 0.97955 | 1.92193 | NLKPP |
| 97-101 | 0.95852 | 1.92193 | RKRPL |
| 222-226 | 0.94971 | 1.92193 | AKRKI |
| 203-207 | 0.94006 | 1.92193 | KRAAP |

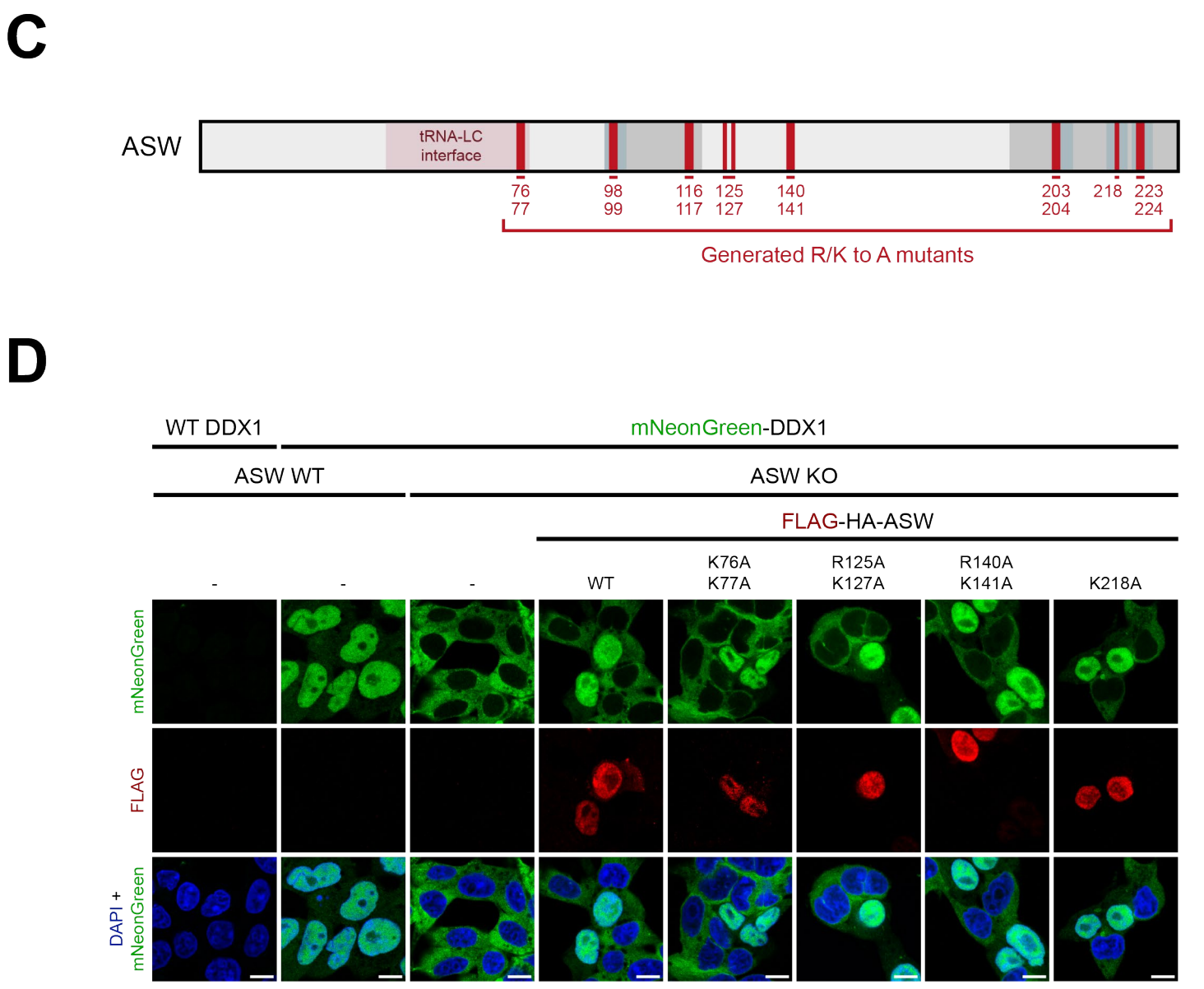


**Supplementary Figure 10: Data related to Figure 5.** (**A**) HEK FITR WT cells were transiently transfected with different FLAG-HA-ASW expression constructs (WT or Δ83-232). As control, cells without any transfected construct (-) were used. The expressed constructs were isolated using an anti-FLAG IP. Input and eluate samples of the IP were analyzed by WB for RTCB, FLAG and Tubulin (evaluation of equal loading), to validate that the Δ83-232 ASW mutant still binds the tRNA-LC. Equal amounts of total protein (input) or volume (eluate) were loaded per lane. Source data are provided as a Source Data file. (**B**) NLS segments in ASW that were predicted by the tool NLSExplorer overlaid on regions of ASW that we identified to contain an NLS. (**C**) Overview of R/K to A mutants that we generated and tested for inactivating the NLSs in ASW. (**D**) HEK FITR ASW KO cells expressing endogenous mNeonGreen-DDX1 were transiently transfected with different FLAG-HA-ASW rescue constructs (WT or R/K to A mutants). Anti-FLAG immunofluorescence was performed and cells were stained with DAPI, to determine the localization of endogenous tRNA-LC as well as the expressed constructs by microscopy. As control, HEK FITR WT and ASW KO cells endogenously expressing mNeonGreen-DDX1 without any transfected construct (-) were used. Scale bars indicate 10 µm.

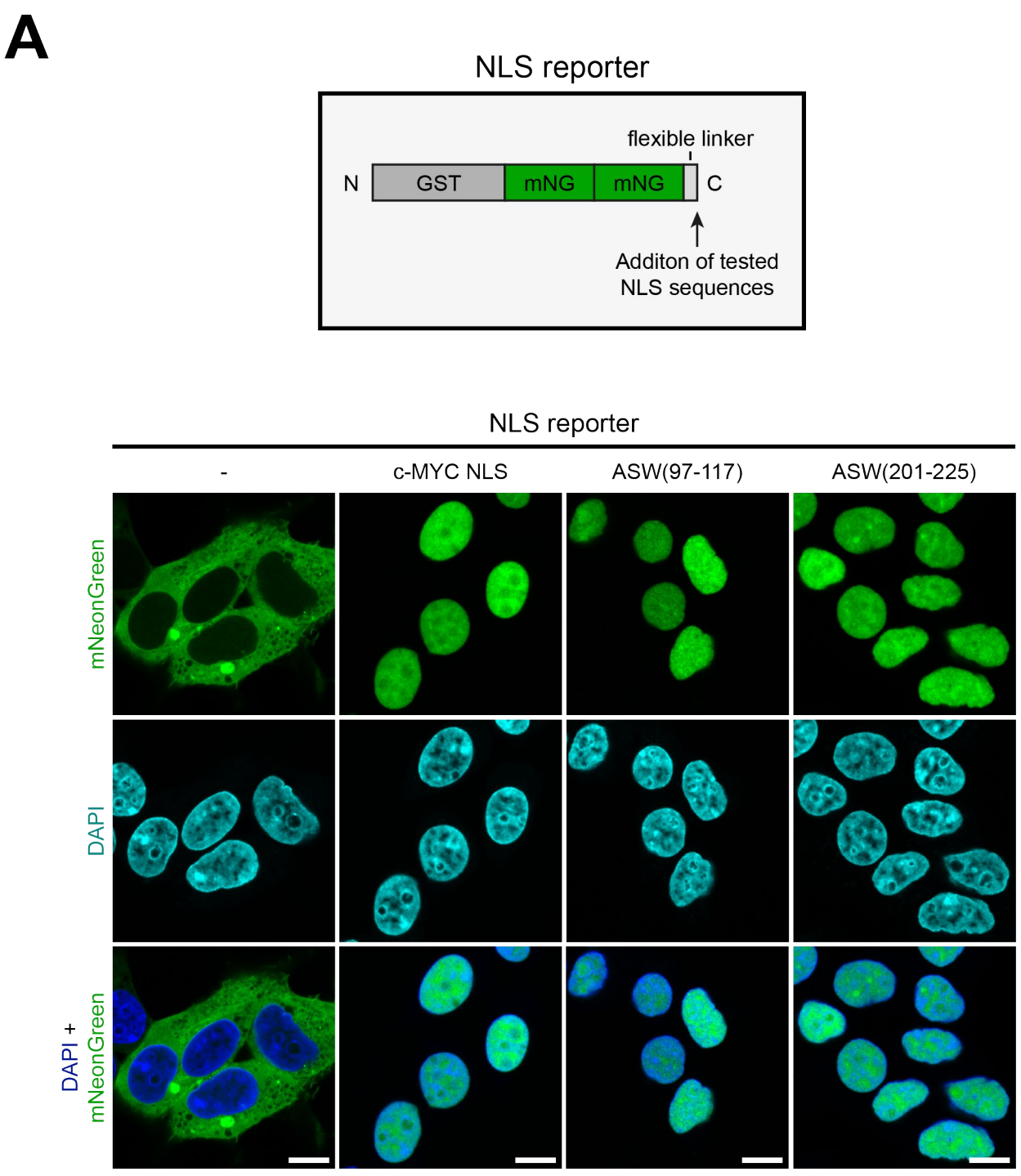


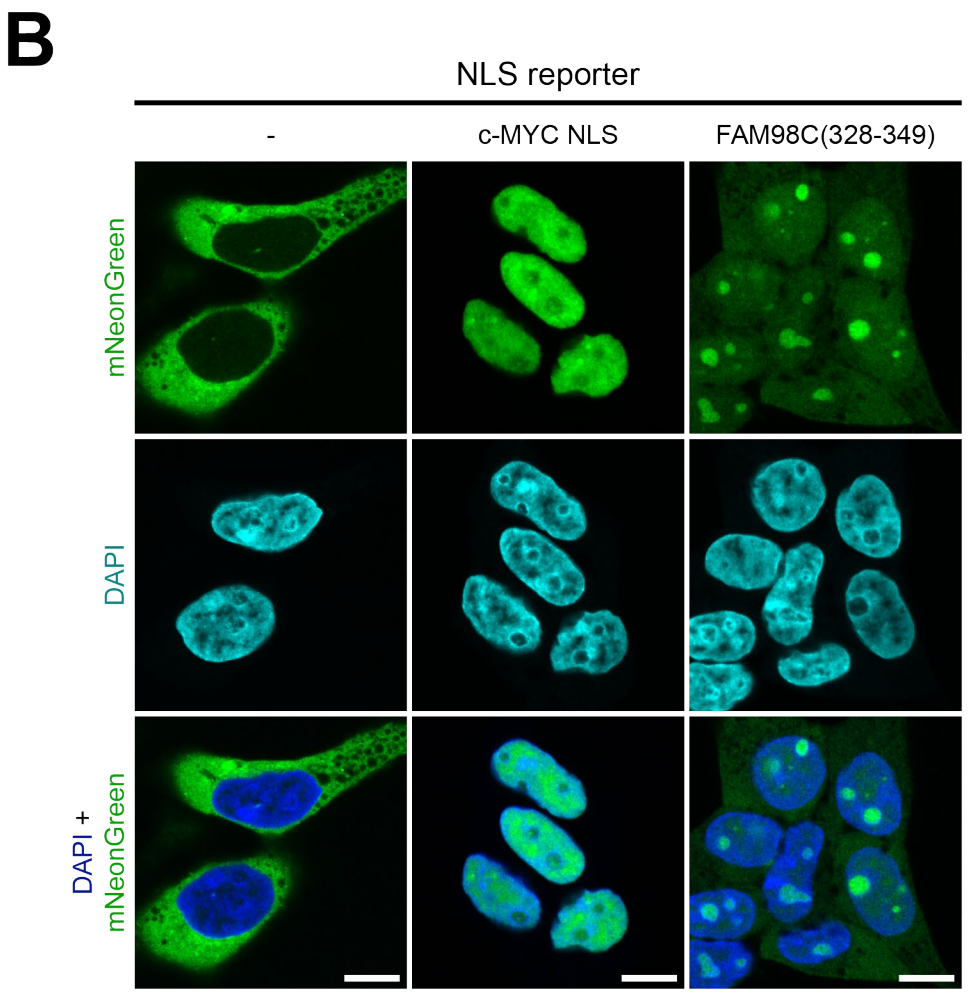


**Supplementary Figure 11: Validation of the identified NLSs of ASW and the C-terminus of FAM98C with a GST-mNG-mNG NLS reporter.** (**A**) HEK FITR cell lines stably carrying doxycycline-inducible expression constructs coding for NLS reporters (GST-mNeonGreen-mNeonGreen) with different C-terminally added sequences [c-MYC NLS, ASW(97-117) or ASW(201-225)] were treated with doxycycline for 24 h. Cells expressing the NLS reporter without any C-terminally added sequence (-) were used as control. The cells were fixed, permeabilized and stained with DAPI, to determine the localizations of the NLS reporters by microscopy. (**B**) Same as in **A**, but testing the C-terminal sequence of FAM98C (residues 328-349) with the GST-mNG-mNG NLS reporter. (**A, B**) Scale bars indicate 10 μm.

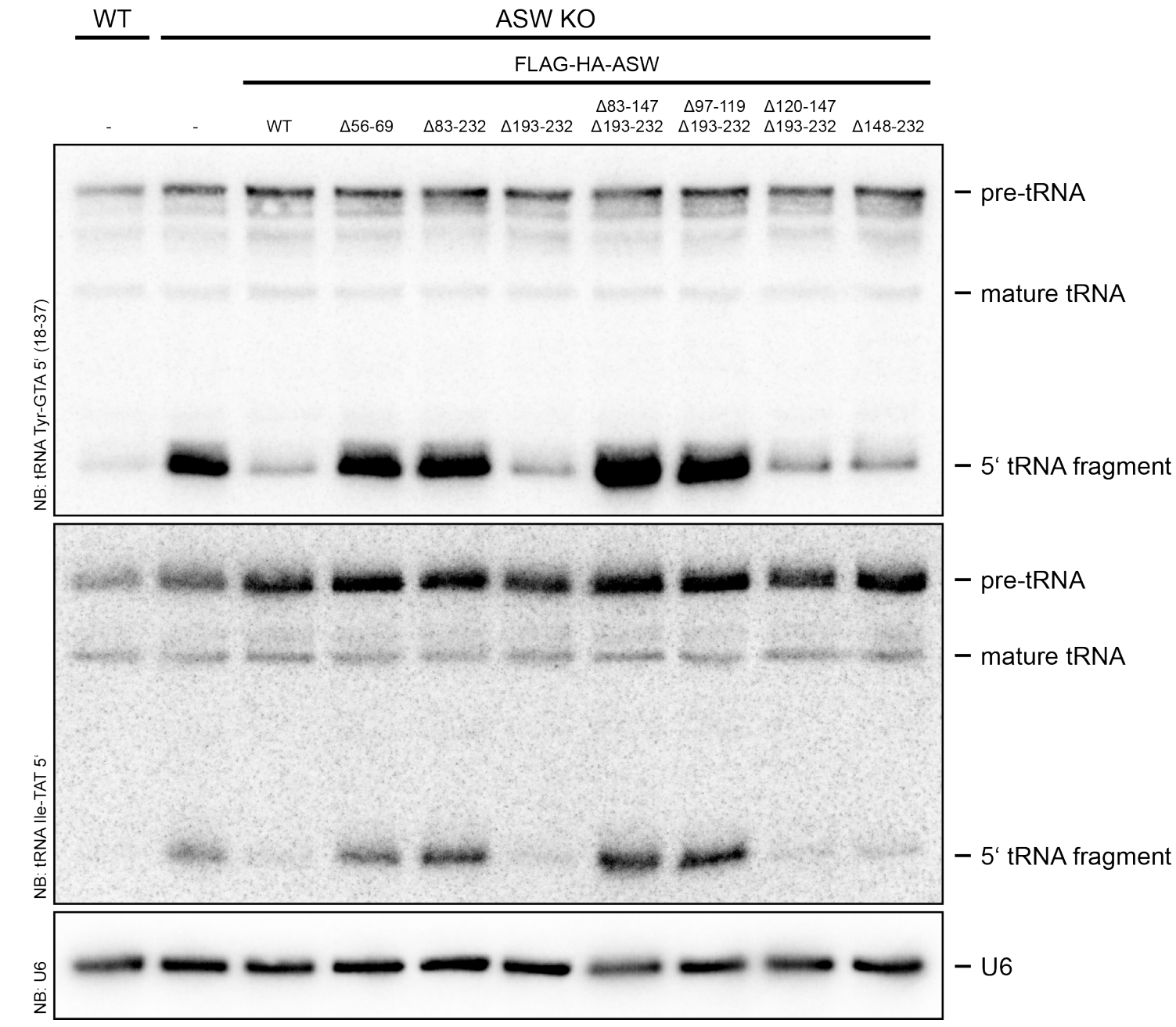


**Supplementary Figure 12: ASW truncation mutants that cannot bind the tRNA-LC or lack both NLSs cannot rescue the pre-tRNA splicing defect of ASW KO cells.** Steady-state levels of tRNA Tyr-GTA and tRNA Ile-TAT as well as their precursors in HEK FITR WT, ASW KO and rescued ASW KO cells were determined by NB. The rescue cell lines stably carried doxycycline-inducible expression constructs for different FLAG-HA-tagged ASW constructs (WT and truncation mutants) and were treated with doxycycline for 48 h prior to RNA isolation. 5 µg total RNA were loaded per lane. A probe against U6 snRNA was used as loading control. Source data are provided as a Source Data file.

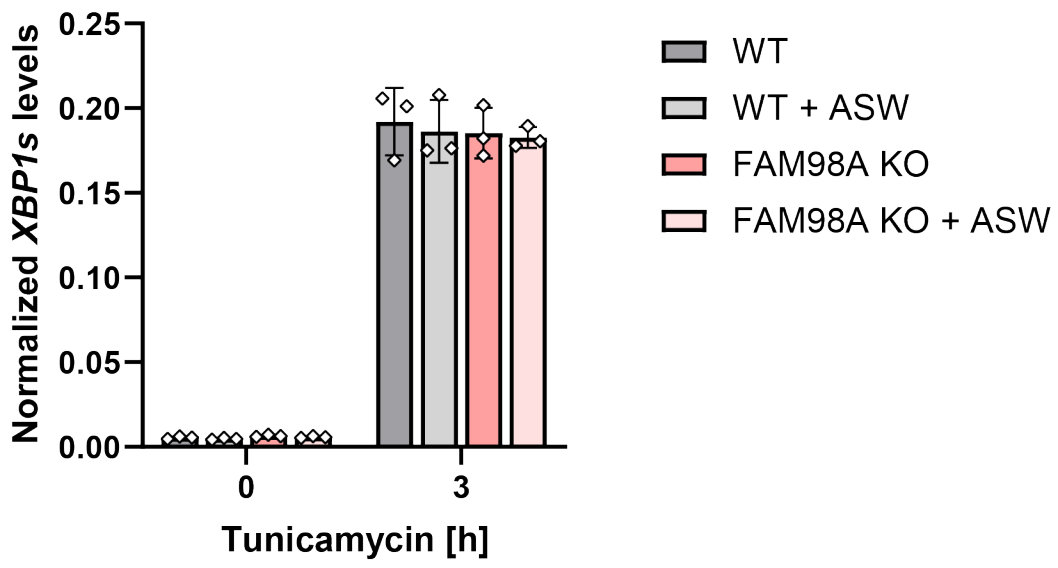


**Supplementary Figure 13: Overexpression of ASW in WT and FAM98A KO cells does not affect *XBP1*-mRNA splicing.** Relative transcript levels of *XBP1s* (spliced) determined by qPCR during tunicamycin-induced UPR in HEK FITR WT and FAM98A KO cells, both without stably integrated expression constructs and with stably integrated FLAG-HA-ASW expression constructs. Construct expression was induced by the addition of doxycycline for 24 h prior to RNA isolation. Expression levels were normalized to *ACTB* mRNA levels. Relative expression levels were calculated with the $2^{-\Delta\Delta Ct}$ method. Values represent means ± SD from three independent experiments. Source data are provided as a Source Data file.

# Supplementary Tables

**Supplementary Table 1: Used gRNA sequences.**

| Name | Used for | Sequence |
|---|---|---|
| gRNA_ASW_KO_pair1_a | ASW KO | ATTTGCCGAAGAATAGATGG |
| gRNA_ASW_KO_pair1_b | | GAGGCAATGGTATTGCATGT |
| gRNA_FAM98A_KO_pair1_a | FAM98A KO | AAGGGCCCATTGTTGGAAGA |
| gRNA_FAM98A_KO_pair1_b | | ACAGAGTTTGGTAAACTCGG |
| gRNA_FAM98B_KO_pair1_a | FAM98B KO | AGGGCTTGCTCTTCTAACAA |
| gRNA_FAM98B_KO_pair1_b | | CTTACAAAGGCGGCAGAGGG |
| gRNA_FAM98C_KO_pair1_a | FAM98C KO | ACTTCAGGGGGCTGTGCGTG |
| gRNA_FAM98C_KO_pair1_b | | CGCCTCTCGCTGCTGCTCGA |
| gRNA_RTCB_N-term | mNeonGreen-tagging RTCB<br>c-MYC-NLS-tagging RTCB | ATTATAGCTGCGACTCATGG |
| gRNA_DDX1_N-term | mNeonGreen-tagging DDX1 | TTCCACAAACGCACCGGAGA |
| gRNA_CGI-99_N-term | mNeonGreen-tagging CGI-99 | CGTCAACTTGCGTCGGAACA |

**Supplementary Table 2: Used northern blot probes and RT-qPCR primers.**

| Name | Used for | Sequence |
|---|---|---|
| NB_probe_Tyr_5p (5-25) | Northern blotting | GCTCTACCAGCTGAGCTATCG |
| NB_probe_Tyr_5p (18-37) | Northern blotting | CTACAGTCCTCCGCTCTACC |
| NB_probe_Ile_5p | Northern blotting | TATAAGTACCGCGCGCTAAC |
| NB_probe_Gly_5p | Northern blotting | TACCACTGAACCACCAATGC |
| NB_probe_Met _5p | Northern blotting | GGGCCCAGCACGCTTCCGCTGCGCCACTCTGC |
| NB_probe_U6 | Northern blotting | GCAGGGGCCACGCTAATCTTCTCTG |
| q_ACTB_FWD | RT-qPCR | GCAGAAGGAGATCACTGCCC |
| q_ACTB_REV | RT-qPCR | GTACTTGCGCTCAGGAGGAG |
| q_XBP1u_FWD | RT-qPCR | ACTACGTGCACCTCTGCAG |
| q_XBP1u_REV | RT-qPCR | GGAAGGGCATTTGAAGAACA |
| q_XBP1s_FWD | RT-qPCR | GAGTCCGCAGCAGGTG |
| q_XBP1s_REV | RT-qPCR | GGAAGGGCATTTGAAGAACA |
| q_XBP1total_FWD | RT-qPCR | GCGCTGAGGAGGAAACTGAAAAAC |
| q_XBP1total_REV | RT-qPCR | CCAAGCGCTGTCTTAACTCC |
