# Supplementary material for: Ashwin and FAM98 paralogs define nuclear and cytoplasmic RNA ligase complexes for tRNA biogenesis
Source: Nat Commun. 2026 Sep 17;17:9882. doi: 10.1038/s41467-026-77451-x (PMC13586215; doi:10.1038/s41467-026-77451-x)
Supplement: Supplementary file 2 [file 41467_2026_77451_MOESM2_ESM.pdf]

Corresponding author(s): Javier Martinez

Last updated by author(s): Jun 16, 2026

Reporting Summary

Nature Portfolio wishes to improve the reproducibility of the work that we publish. This form provides structure for consistency and transparency in reporting. For further information on Nature Portfolio policies, see our Editorial Policies and the Editorial Policy Checklist.

Statistics

For all statistical analyses, confirm that the following items are present in the figure legend, table legend, main text, or Methods section.

n/a	Confirmed	
☐	☒	The exact sample size (*n*) for each experimental group/condition, given as a discrete number and unit of measurement
☐	☒	A statement on whether measurements were taken from distinct samples or whether the same sample was measured repeatedly
☐	☒	The statistical test(s) used AND whether they are one- or two-sided<br>*Only common tests should be described solely by name; describe more complex techniques in the Methods section.*
☒	☐	A description of all covariates tested
☐	☒	A description of any assumptions or corrections, such as tests of normality and adjustment for multiple comparisons
☐	☒	A full description of the statistical parameters including central tendency (e.g. means) or other basic estimates (e.g. regression coefficient) AND variation (e.g. standard deviation) or associated estimates of uncertainty (e.g. confidence intervals)
☐	☒	For null hypothesis testing, the test statistic (e.g. *F*, *t*, *r*) with confidence intervals, effect sizes, degrees of freedom and *P* value noted<br>*Give P values as exact values whenever suitable.*
☒	☐	For Bayesian analysis, information on the choice of priors and Markov chain Monte Carlo settings
☒	☐	For hierarchical and complex designs, identification of the appropriate level for tests and full reporting of outcomes
☒	☐	Estimates of effect sizes (e.g. Cohen's *d*, Pearson's *r*), indicating how they were calculated

Our web collection on statistics for biologists contains articles on many of the points above.

Software and code

Policy information about availability of computer code

Data collection	RT-qPCR: Performed in a BioRad CFX384 Touch thermocycler, operated with BioRad CFX Maestro software (version 4.0.2325.0418). Cq values were determined from the amplification curves by the BioRad CFX Maestro software.<br>Microscopy: Images were acquired using an inverse point scanning confocal Zeiss LSM980 Microscope equipped with a Plan-Apochromat 40x/1.4 Oil DIC M27 (WD 0.13mm) objective, running with Zeiss ZEN blue 3.3 software (version 3.3.89.0008).<br>Western blots: Chemiluminescence and colorimetric images were acquired with a BioRad ChemiDoc Touch Imaging System (software version 2.3.0.07).<br>Northern blots: Images were acquired with a Cytiva Amersham Typhoon (software version 3.0.0.2)<br>Growth curves: Images were acquired and analyzed with a Sartorius Incucyte Live-Cell Analysis system (software versions 2025C).<br>Structural predictions: AlphaFold 3 (webserver, v3.0.0).<br>NLS predictions: NLSExplorer (webserver).<br>Ortholog clusters: data was retrieved from OrthoDB (v11).<br>Mass-spec (FAM98 co-IP): data was collect with an UltiMate 3000 RSLCnano LC system (Thermo Scientific) coupled to a timsTOF HT (Bruker).<br>Mass-spec (FAM98C KO validation): data was collected with a Vanquish Neo UHPLC system (Thermo Scientific) coupled to an Orbitrap Exploris 480 mass spectrometer (Thermo Scientific).
Data analysis	GraphPad Prism 10.5.0: generation of plots, statistical analyses.<br>Fiji/ImageJ 2.16.0/ 1.54p: processing and quantification of western and northern blots; processing of microscopy images.<br>Microsoft Excel Professional Plus 2021: analysis of qPCR data, analysis of quantification data from western and northern blots.<br>R 4.4.1, using Growthcurver 0.3.1: analysis of growth curves.<br>Clustal Omega (EMBL-EBI webserver): MSA generation from vertebrate ASW sequences.<br>Jalview 2.11.5.1: analysis of human ASW residue conservation from an MSA.

CellProfiler 4.2.8: analysis and quantification of microscopy images.
ChimeraX 1.11: used to visualize predicted structures.
Skyline 24.1.1.284: mass-spec data processing and analysis.
Spectronaut 19.1: mass-spec data processing and analysis.
MsReport 0.0.25: mass-spec data processing and analysis.
LIMMA 3.52.1: mass-spec data analysis.

For manuscripts utilizing custom algorithms or software that are central to the research but not yet described in published literature, software must be made available to editors and reviewers. We strongly encourage code deposition in a community repository (e.g. GitHub). See the Nature Portfolio guidelines for submitting code & software for further information.

Data

Policy information about availability of data

All manuscripts must include a data availability statement. This statement should provide the following information, where applicable:

- Accession codes, unique identifiers, or web links for publicly available datasets
- A description of any restrictions on data availability
- For clinical datasets or third party data, please ensure that the statement adheres to our policy

Raw mass spectrometry data were deposited to the ProteomeXchange Consortium via the PRIDE partner repository with the dataset identifiers PXD076703 (co-IP/MS of the human FAM98 proteins) and PXD076694 (FAM98C KO validation). Other data that support the findings of this study are available within the paper and its supplementary information. Source data are provided with this paper.

Research involving human participants, their data, or biological material

Policy information about studies with human participants or human data. See also policy information about sex, gender (identity/presentation), and sexual orientation and race, ethnicity and racism.

Reporting on sex and gender	NA
Reporting on race, ethnicity, or other socially relevant groupings	NA
Population characteristics	NA
Recruitment	NA
Ethics oversight	NA

Note that full information on the approval of the study protocol must also be provided in the manuscript.

Field-specific reporting

Please select the one below that is the best fit for your research. If you are not sure, read the appropriate sections before making your selection.

☒ Life sciences ☐ Behavioural & social sciences ☐ Ecological, evolutionary & environmental sciences

For a reference copy of the document with all sections, see nature.com/documents/nr-reporting-summary-flat.pdf

Life sciences study design

All studies must disclose on these points even when the disclosure is negative.

Sample size

No statistical methods were used to predetermine sample size. Sample sizes were chosen based on our previous experience and similar studies published previously.
RT-qPCR was performed in three biological replicates and each individual sample was measured in technical triplicates. This sample size was chosen because it allows for statistical analysis. Since we were in all cases able to replicate our results, we deemed the sample size sufficient.
Mass spectrometry experiments were performed in biological triplicates. This sample size was chosen because it allows for statistical analysis. Since we were in all cases able to replicate our results, we deemed the sample size sufficient.
IPs followed by western blotting were performed in biological duplicates (ASW tRNA-LC interface mutants, FAM98C KO validation by co-IP) or triplicates (other IP/WB experiments). Since we were in all cases able to replicate our results, we deemed the sample size sufficient.
Microscopy experiments were performed in two or more biological replicates, with multiple images per sample and replicate. Since we were in all cases able to replicate our results, we deemed the sample size sufficient.
Western blot experiments showing the effects on expression levels of tRNA-LC subunits in different KO conditions were performed as biological duplicates (FAM98 KO) or triplicates (ASW KO). Since we were in all cases able to replicate our results, we deemed the sample size sufficient.
Northern blot experiments were performed in three or more biological replicates. This sample size was chosen because it allows for statistical analysis. Since we were in all cases able to replicate our results, we deemed the sample size sufficient.
Puromycin-incorporation experiments were performed in five biological replicates. This sample size was chosen because we observed greater variation throughout the replicates than in other experiments and thus wanted to have a bigger sample size for more powerful statistical

analyses.
Growth curve determination was performed in 6 biological replicates. This sample size was chosen because it allows for powerful statistical analysis. Since we were in all cases able to replicate our results, we deemed the sample size sufficient.
Western blots during cell line generation were performed once, since they were meant to confirm the genotype and protein expression and not to generate experimental data.

Data exclusions

For analysis of the nuclear-cytoplasmic distribution of the tRNA-LC from microscopy images, cells were automatically identified and segmented. This process was error-prone and we thus manually went through the data and excluded quantified cells that: were undergoing cell division (condensed chromatin), had wrongly defined boundaries (either for the cell or the nucleus) or had more than one nucleus (multiple cells identified as a single one). Furthermore, when quantifying cells expressing a FLAG-tagged construct, we excluded cells that did not express the construct (low anti-FLAG signal, similar to cells not expressing a FLAG-tagged protein; cut-off value was the same for all cells within an experiment). These criteria were pre-established in the experimental design.
We honestly believe that such a processing scheme is common practice for these kind of experiments and thus we are not sure whether it actually counts as data exclusion or not, but just want to be fully transparent.
No other data were excluded.

Replication

All attempts at replication were successful.
RT-qPCR was performed in three biological replicates with similar results, each individual sample was measured in technical triplicates.
Mass spectrometry experiments were performed in three biological replicates, results were successfully replicated.
IP experiments followed by western blotting were performed in two or three biological replicates, results were successfully replicated.
Microscopy experiments were performed in two or more biological replicates, with multiple images per sample and replicate. Results were successfully replicated.
Western blot experiments showing the effects on expression levels of tRNA-LC subunits in different KO conditions were performed in two or three biological replicates, results were successfully replicated.
Northern blot experiments were performed in three or more biological replicates and results were successfully replicated.
Puromycin-incorporation experiments were performed in five biological replicates with similar results.
Growth curve determination was performed in 6 biological replicates. Results were successfully replicated.
Western blots meant to confirm successful CRISPR/Cas9 editing rather than generate experimental data were not replicated.

Randomization

Randomization was not performed for this study. Samples (cell lines) were allocated to experimental groups based on genotype and paired with a control, i.e. KO cell lines were compared to a control WT cell line or cells expressing FLAG-HA-tagged proteins were compared to control cells not expressing a construct. All cell lines were grown under the same conditions and experiments were performed under identical conditions.

Blinding

The investigators were not blinded in this study since they were involved in the planning, execution and analysis of the experiments. Within-experiment sample groups and the respective controls were prepared, processed and analysed at the same time and under identical conditions to eliminate any bias, so prior knowledge had no impact on data output. No subjective process was involved in the analysis of the data. Analysis of mass spectrometry data was carried out bioinformatically applying the same parameters to all samples without need for investigator blinding.

Reporting for specific materials, systems and methods

We require information from authors about some types of materials, experimental systems and methods used in many studies. Here, indicate whether each material, system or method listed is relevant to your study. If you are not sure if a list item applies to your research, read the appropriate section before selecting a response.

Materials & experimental systems

n/a	Involved in the study	
☐	☒	Antibodies
☐	☒	Eukaryotic cell lines
☒	☐	Palaeontology and archaeology
☒	☐	Animals and other organisms
☒	☐	Clinical data
☒	☐	Dual use research of concern
☒	☐	Plants

Methods

n/a	Involved in the study	
☒	☐	ChIP-seq
☒	☐	Flow cytometry
☒	☐	MRI-based neuroimaging

Antibodies

Antibodies used

Primary:
anti-FLAG (Sigma Aldrich, F1804, clone M2; used 1:1000 for WB and 1:200 for IF)
anti-RTCB (Bethyl Laboratories, A305-079A, polyclonal; used 1:2000)
anti-DDX1 (Bethyl Laboratories, A300-521A, polyclonal; used 1:2000)
anti-FAM98A (Abcam, ab204083, polyclonal; used 1:1000)
anti-FAM98B (Thermo, PA5-52568, polyclonal; used 1:1000)
anti-FAM98C (Thermo, PA5-59356, polyclonal; used 1:1000)
anti-CGI-99 (Abcam, ab188326, clone EPR15799; used 1:2000)
anti-ASW (self-made, clone 1C6-H6; mouse monoclonal against recombinant full-length human ASW, expressed and purified from E. coli; sequence of the epitope: UniProt Q9BVC5-1; used 1:100)
anti-Puromycin (Sigma Aldrich, ZMS1016, clone 12D10; used 1:5000)

anti-Tubulin (Abcam, ab44928, mix of clones DM1A and DM1B; used 1:2000)
anti-ACTA1 (Sigma Aldrich, A2066, polyclonal; used 1:1000)
anti-GAPDH (Cell Signaling, 2118, clone 14C10; used 1:2000)

Secondary:
anti-mouse Alexa Fluor Plus 647 (Invitrogen, A32728; used 1:1000)
anti-rabbit, HRP (Invitrogen, 65-6120; used 1:5000)
anti-mouse, HRP (Invitrogen, 62-6520; used 1:5000)

Validation

IF:
anti-FLAG: this study (4C, 5B, 5C, 6B, S9A, S10D), signal only detectable in cells that express a FLAG-tagged protein.

WB:
anti-FLAG: this study (1D, 1F, S1A, S4B, S5, S7B, S10A), signal only detectable in cells that express a FLAG-tagged protein; also used in >2000 publications, for example used for WBs in https://www.nature.com/articles/s41467-025-56564-9
anti-RTCB: this study (7A, S8), the band that is detected in WT cells has the right size and when endogenous RTCB is homozygously tagged, the band completely shifts to the expected size of the tagged RTCB; also used in https://linkinghub.elsevier.com/retrieve/pii/S1097-2765(21)00312-9
anti-DDX1 (S8): this study, same principle as for RTCB antibody validation; also validated in https://www.nature.com/articles/nature13284
anti-FAM98A: this study (2A, 7A), a single band detected at the expected size in WT cells, no detectable bands in FAM98A KO cells
anti-FAM98B: this study (2A), two bands detected at the expected size in WT cells (FAM98B , no detectable bands in FAM98B KO cells
anti-FAM98C: this study (1F, S4B), single band with the right size in elutions of RTCB IPs, but only from WT cells, this band is not detectable when doing RTCB IPs from MS-validated FAM98C KO cells; however, several unspecific bands in western blots of whole cell lysates from HEK cells; we therefore only used this antibody for a readout of RTCB IPs
anti-CGI-99: this study (S8), same principle as for RTCB antibody validation; also validated in https://link.springer.com/article/10.1186/s12967-016-0801-4
anti-ASW: this study (3A), a detected band at the expected size in WT cells, no detectable bands in ASW KO cells
anti-Puromycin: this study (S6D), no detectable bands in cells that were not treated with puromycin
anti-Tubulin: used in >100 publications; for example in https://www.nature.com/articles/ncomms8889 or https://www.nature.com/articles/s41586-018-0353-2
anti-ACTA1: used in >4000 publications; for example in https://www.nature.com/articles/s41467-022-31905-0
anti-GAPDH: used in >1000 publications; for example in https://www.nature.com/articles/s41467-025-56564-9

Eukaryotic cell lines

Policy information about cell lines and Sex and Gender in Research

Cell line source(s)	HEK Flp-In T-REx 293 cells were acquired from Thermo Fisher Scientific (R78007). All other cell lines used in this paper (knock-in and knock-out cell lines) were generated by us from this starting cell line.
Authentication	The cell line was not authenticated.
Mycoplasma contamination	All cell lines that we used were regularly tested for mycoplasma contaminations (qPCR-based) and tested negative.
Commonly misidentified lines (See ICLAC register)	No commonly misidentified cell lines were used in this study.

Plants

Seed stocks	*Report on the source of all seed stocks or other plant material used. If applicable, state the seed stock centre and catalogue number. If plant specimens were collected from the field, describe the collection location, date and sampling procedures.*
Novel plant genotypes	*Describe the methods by which all novel plant genotypes were produced. This includes those generated by transgenic approaches, gene editing, chemical/radiation-based mutagenesis and hybridization. For transgenic lines, describe the transformation method, the number of independent lines analyzed and the generation upon which experiments were performed. For gene-edited lines, describe the editor used, the endogenous sequence targeted for editing, the targeting guide RNA sequence (if applicable) and how the editor was applied.*
Authentication	*Describe any authentication procedures for each seed stock used or novel genotype generated. Describe any experiments used to assess the effect of a mutation and, where applicable, how potential secondary effects (e.g. second site T-DNA insertions, mosiacism, off-target gene editing) were examined.*
