# Supplementary material for: Ashwin and FAM98 paralogs define nuclear and cytoplasmic RNA ligase complexes for tRNA biogenesis
Source: Nat Commun. 2026 Sep 17;17:9882. doi: 10.1038/s41467-026-77451-x (PMC13586215; doi:10.1038/s41467-026-77451-x)
Supplement: Supplementary file 3 [file 41467_2026_77451_MOESM3_ESM.pdf]

nature portfolio

Peer Review File

Ashwin and FAM98 paralogs define nuclear and cytoplasmic RNA ligase complexes for tRNA biogenesis

Corresponding Author: Professor Javier Martinez

This file contains all reviewer reports in order by version, followed by all author rebuttals in order by version.

Version 0:

Reviewer comments:

Reviewer #1

(Remarks to the Author)
RTCB-type RNA ligases are found across all kingdoms of life. They catalyze the GTP-dependent ligation of RNA strands by the formation of new phosphodiester bonds. RTCB ligases fulfill various important functions in RNA processing and repair mechanisms depending on the respective species. In metazoans, RTCB plays an essential role in tRNA splicing as part of pre-tRNA maturation, as well as the non-conventional mRNA splicing in the IRE1 branch of the unfolded protein response (UPR). In the course of evolution, an expanding set of accessory proteins has evolved to assist RTCB in RNA ligation. In 2011, the Martinez Lab identified human RTCB as the key ligase enzyme in tRNA splicing and described it as part of a stable pentameric tRNA ligase complex (tRNA-LC) including the subunits DDX1, CGI-99, FAM98B, and ASW (Ashwin). While the understanding of the overall architecture of the complex has increased, particularly through recent cryo-EM structures, the role of the individual subunits has remained largely unknown.
In this manuscript by the Martinez Lab, Leitner et al report a cellular role of ASW in nuclear localization of the tRNA-LC and the formation of subpopulations of the complex based on FAM98 paralogs. The authors present in a concise way that ASW and FAM98B are linked as subunits of a (predominantly) nuclear subpopulation of the tRNA-LC. The study presents clear evidence for the presence of two NLS segments within ASW, thus identifying a new cellular role for this subunit in ensuring sufficient ligase activity in the nucleus. The A and C paralogs of FAM98 do not interact with ASW. While FAM98A promotes cytoplasmic localization of the tRNA-LC, FAM98C promotes nuclear localization independent of ASW. The main findings of this study regarding subcellular localization are well substantiated by the experimental results. However, some of the functional implications of the distinct subpopulations of the tRNA-LC require further clarification.
In summary, I support publication of this manuscript by Leitner et al in Nature Communications after resolving the following comments.
- The results section begins by mentioning co-evolutionary analyses and phylogenetic distribution. I think it is remarkable how the authors use such seemingly straightforward analyses to make important discoveries about the tRNA-LC and its partner proteins. Could the authors expand these analyses to the level of conservation of functional regions within the FAM98 paralogs? In light of the recent cryoEM structures of truncated versions of the FAM98B/ASW-containing complex, are the residues within the ASW/FAM98B interface conserved? Can the exclusive binding of ASW to the B paralog be explained at the sequence level when comparing the interface residues to the A and C paralogs?
- On a related note, there is good agreement between the recently published EM structure (Glatt/Nowotny labs) and AlphaFold3 predictions of the complex. I think the authors could use AF3 models with the A and C paralogs to provide additional insights into the exclusive interaction specificity.
- FAM98C promotes nuclear localization independent of ASW (Fig. 3C). Have the authors analyzed the C paralog for an NLS? I would appreciate a short discussion of how nuclear localization of FAM98C is achieved. I do not request additional experiments, but presumptive NLS segments could be tested using the same experimental setup as in Fig. 3.
- In all depicted Northern blots, the levels of mature tRNA seem surprisingly low compared to intron-containing pre-tRNA. The authors should comment on that.
- The authors did not detect an impact on XBP1 mRNA splicing in the various FAM98 KO cell lines. It would be interesting to see if a combination of FAM98A KO with RTCB-NLS or FAM98B overexpression would show any reduction in XBP1s. Similarly, the levels of mature tRNA are not reduced in any of the KO cell lines, despite fragment accumulation in some cases. While the authors fully acknowledge and address these aspects in the discussion, the inclusion of orthogonal sequencing approaches would strengthen the findings. As mentioned above, the low levels of mature tRNA in the Northern blots complicate the interpretation further.
Minor points:

- The authors should consider changing “destabilized” to something more neutral when describing the results in Fig. 2A (page 5, line 126). Primarily, they observed reduced levels. Whether the protein levels are reduced by increased degradation/destabilization, by some feedback mechanisms leading to reduced expression, or a combination of multiple effects, is difficult to discern. Does overexpression of the A or C paralogs return the levels of all subunits except for ASW back to normal?

Reviewer #2

(Remarks to the Author)
This study demonstrates a role for the ASW subunit of the vertebrate tRNA ligase complex (tRNA-LC) in localizing the ligase to the nucleus where tRNA splicing occurs and shows that ASW binding to complex subunit FAM98B (one of three FAM98 paralogs) specifically leads to nuclear localization of FAM98B-containing ligase complexes. By contrast, complexes containing paralog FAM98A reside in the cytoplasm where they perform noncanonical mRNA splicing in the unfolded protein response. In a clever maneuver, the authors show that appending an NLS to RtcB restores nuclear localization of tRNA-LC, and reverses the accumulation of 5’ tRNA fragments, in cells deleted for ASW. These are important findings.

Comments:

CRISPR-knockouts of the FAM98 paralog genes and ASW in 293 cells reveal that deletion of FAM98B or ASW specifically leads to accumulation of tRNA 5’ exon fragments. It is most noteworthy that these KO cells do not have lower steady-state levels of the mature spliced forms of these same tRNAs. This would seem to call into question the assertion (line 123) that “FAM98B and ASW are ESSENTIAL for effective pre-RNA splicing”. Given that tRNA splicing is essential per se for cell viability, it would behoove the authors to show data regarding the growth properties of the cell lines in which ASW and FAM98B are deleted. Is there a defect in protein synthesis?

Taken at face value, the results suggest that cells are able to somehow compensate for the loss of ASW and FAM98B and continue to splice intron-containing tRNAs. How might this happen? Does a residual level of nuclear localization of tRNA-LC sustain tRNA splicing in their absence? The authors favor this idea in the Discussion. (Alternatively, can some level of tRNA splicing occur in the cytoplasm when ASW and FAM98B are missing?)

The accumulation of 5’ tRNA fragments is taken as synonymous with a primary defect in exon ligation. I would suggest strongly that the authors also assay by Northerns the fate of the 3’ exons. Also, it would be very valuable to separate nucleus and cytoplasm and determine the compartment in which the 5’-tRNA fragments accumulate.

Is it possible that that 5’ fragments accumulate because of failure to repair tRNA breaks inflicted “off-pathway” from the cleavage of pre-tRNA by TSEN? Precise mapping the 3’-ends of the 5’-fragments (by Seq) would provide valuable information in that regard, i.e., whether the 3’ ends correspond to the cleaved exon-intron junction.

Given that the Northern analysis performed on total RNA, it is surprising that the steady state levels of pre-tRNA consistently exceed those of the mature tRNA. How to explain that seeming anomaly?

Minor point:
Lines 117-119: “band” and “bands” are inappropriate slang terms. Change to “polypeptide” and “polypeptides”.

Version 1:

Reviewer comments:

Reviewer #1

(Remarks to the Author)
During revision, the authors of Leitner et al. have extensively addressed reviewers’ comments and added new experiments, data and analyses. I have re-evaluated the revised version of the manuscript and the detailed point-by-point response provided by the authors. The authors have thoroughly addressed my remarks. I especially appreciate the systematic optimization of the Northern blotting protocols and nuanced discussion of the results.
I support publication of this manuscript in Nature Communications and commend the authors on their work.
Jirka Peschek

Reviewer #2

(Remarks to the Author)
The revised paper is improved as the authors have now performed additional experiments to optimize tRNA detection by Northern blotting at reduced annealing temperature (Fig. 2B). This resolved major concerns with the original data that seriously underestimated the level of mature tRNA while showing accumulation of an unligated 5’ tRNA half. As requested, they also now document the presence of 3’ tRNA half. Requested assays of cell growth and protein synthesis have been added.

It is unclear why they did not incorporate the technical improvement to redo the Northerns shown in Fig. 5A, B and E (seemingly unchanged from the original version, where mature tRNA is nearly undetectable). Doing so would be a plus.

Open Access This Peer Review File is licensed under a Creative Commons Attribution 4.0 International License, which permits use, sharing, adaptation, distribution and reproduction in any medium or format, as long as you give appropriate credit to the original author(s) and the source, provide a link to the Creative Commons license, and indicate if changes were made.
In cases where reviewers are anonymous, credit should be given to 'Anonymous Referee' and the source.
The images or other third party material in this Peer Review File are included in the article's Creative Commons license, unless indicated otherwise in a credit line to the material. If material is not included in the article's Creative Commons license and your intended use is not permitted by statutory regulation or exceeds the permitted use, you will need to obtain permission directly from the copyright holder.
To view a copy of this license, visit https://creativecommons.org/licenses/by/4.0/

Reviewer #1 (Remarks to the Author):

RTCB-type RNA ligases are found across all kingdoms of life. They catalyze the GTP-dependent ligation of RNA strands by the formation of new phosphodiester bonds. RTCB ligases fulfill various important functions in RNA processing and repair mechanisms depending on the respective species. In metazoans, RTCB plays an essential role in tRNA splicing as part of pre-tRNA maturation, as well as the non-conventional mRNA splicing in the IRE1 branch of the unfolded protein response (UPR). In the course of evolution, an expanding set of accessory proteins has evolved to assist RTCB in RNA ligation. In 2011, the Martinez Lab identified human RTCB as the key ligase enzyme in tRNA splicing and described it as part of a stable pentameric tRNA ligase complex (tRNA-LC) including the subunits DDX1, CGI-99, FAM98B, and ASW (Ashwin). While the understanding of the overall architecture of the complex has increased, particularly through recent cryo-EM structures, the role of the individual subunits has remained largely unknown.
In this manuscript by the Martinez Lab, Leitner et al report a cellular role of ASW in nuclear localization of the tRNA-LC and the formation of subpopulations of the complex based on FAM98 paralogs. The authors present in a concise way that ASW and FAM98B are linked as subunits of a (predominantly) nuclear subpopulation of the tRNA-LC. The study presents clear evidence for the presence of two NLS segments within ASW, thus identifying a new cellular role for this subunit in ensuring sufficient ligase activity in the nucleus. The A and C paralogs of FAM98 do not interact with ASW. While FAM98A promotes cytoplasmic localization of the tRNA-LC, FAM98C promotes nuclear localization independent of ASW. The main findings of this study regarding subcellular localization are well substantiated by the experimental results. However, some of the functional implications of the distinct subpopulations of the tRNA-LC require further clarification.
In summary, I support publication of this manuscript by Leitner et al in Nature Communications after resolving the following comments.

We thank the reviewer for this precise summary of our findings.

Major points:

1) The results section begins by mentioning co-evolutionary analyses and phylogenetic distribution. I think it is remarkable how the authors use such seemingly straightforward analyses to make important discoveries about the tRNA-LC and its partner proteins. Could the authors expand these analyses to the level of conservation of functional regions within the FAM98 paralogs? In light of the recent cryoEM structures of truncated versions of the FAM98B/ASW-containing complex, are the residues within the ASW/FAM98B interface conserved? Can the exclusive binding of ASW to the B paralog be explained at the sequence level when comparing the interface residues to the A and C paralogs?

We thank the reviewer for the feedback and the interest in deepening our evolutionary analysis. We tried to identify residues/regions within FAM98A and FAM98C that are unique and conserved as well as potentially important for ASW interaction, in this case by using the ASW/FAM98B interface from the solved human tRNA-LC structure from Pfleiderer et al., co-submitted with our manuscript.

In terms of conservation, we first built a consensus sequence for each FAM98 paralog from MSAs (Clustal Omega and EMBOSS Cons) of ortholog sequences from mammals (419 sequences for FAM98A, 399 for FAM98B and 398 for FAM98C). We then aligned the three consensus sequences (T-Coffee) and looked at differences in FAM98A and C in regions where FAM98B would have residues that bind ASW. However, we could only find minimal differences in these regions. Most notably:

a) A glutamate (E) that is conserved in FAM98A and FAM98B (E21 in human FAM98B) which interacts with R69 of ASW, is a leucine in FAM98C. In the MSA of FAM98C sequences, the position corresponding to residue 21 of human FAM98B is occupied by L (68.6 %), Q (15.8 %), R (7.8 %) and some outliers with < 1 % (the only acidic amino acid occurring here is E with 0.3 %). In the FAM98A MSA, this position is occupied by E (98.8 %), D (0.2 %) and G (0.2 %), while in the FAM98B MSA, this position is occupied by E (96.5 %), D (1.0 %) and A (0.3 %). Also, there are slight differences in the surrounding residues in FAM98C, which seem to be involved in CGI-99 binding. In turn, since CGI-99 is also binding ASW, these changes might position CGI-99 slightly differently in the FAM98C tRNA-LC and thus also prevent ASW-binding.
b) A tyrosine that is highly conserved in FAM98B (Y258 in human FAM98B) which is involved in the binding of ASW, is not conserved in FAM98A and FAM98C. In the FAM98A MSA, this position is occupied by S (96.7 %), T (2.4 %) and L (0.2 %). In the FAM98C MSA, this position is occupied by E (79.4 %), G (11.3 %), Q (2.8 %), K (2.0 %), R (1.5 %) and some outliers < 1 %. Again, for FAM98C, the surrounding residues are also slightly different, which might have an impact as well.

Overall, FAM98C seems to have more sequence changes that might hinder the binding of ASW when compared to FAM98A.

We did not perform experiments to validate the importance of these residues, as a concurrent manuscript by Pfleiderer et al. dissects the different FAM98 tRNA-LCs on the biochemical and structural level, testing which parts of the FAM98 paralogs enable or prevent the binding of ASW. To avoid redundancy, we would like to refer to their manuscript for experimental data on the ASW-enabling or -disabling features of the FAM98 paralogs. The manuscript is available as pre-print on bioRxiv (doi: https://doi.org/10.1101/2025.08.01.668197).

2) On a related note, there is good agreement between the recently published EM structure (Glatt/Nowotny labs) and AlphaFold3 predictions of the complex. I think the authors could use AF3 models with the A and C paralogs to provide additional insights into the exclusive interaction specificity.

We thank the reviewer for this suggestion. We previously predicted the A and C complexes, including ASW, with AF2. Interestingly, all models predicted that ASW should assemble into the complex independently of the assembled FAM98 paralog. We got similar results when repeating the predictions using AF3 (see **Supplementary Fig. 1C**). We believe that the differences between FAM98 paralogs that

ultimately allow or prevent ASW-binding are subtle, and AF3 fails to model them. In our opinion, this increases the value of our experiments, and highlights currently existing limits for structural predictions.

3) FAM98C promotes nuclear localization independent of ASW (Fig. 3C). Have the authors analyzed the C paralog for an NLS? I would appreciate a short discussion of how nuclear localization of FAM98C is achieved. I do not request additional experiments, but presumptive NLS segments could be tested using the same experimental setup as in Fig. 3.

We thank the reviewer for this request. We tried two NLS prediction algorithms on the sequence of human FAM98C:

- NLSExplorer:
  - No recommended NLS segment
  - Highest scoring fragment: residues 343-349 (RKKKKKK) with a recommendation score of 0.24523
- NLStradamus (2 state HMM static):
  - Predicted an NLS: residues 342-349 (GRKKKKKK) (both Viterbi Path and Posterior @ 0.6)

From the predictions, it seemed that the C-terminus of FAM98C would potentially enable the nuclear localization. The C-terminus of FAM98C is also the most unique feature of FAM98C among the FAM98 paralogs, as it is highly enriched in basic amino acids:

Human FAM98C residues 331-349: SRREDGGPQCWGRKKKKKK

While not specifically requested, we decided to clone the C-terminus of FAM98C (residues 328-349) onto our GST-mNG-mNG NLS reporter and expressed it in HEK cells. The construct localized to both cytoplasm and nucleus, but surprisingly accumulated in nucleoli (see **Supplementary Fig. 5**). While this does not match the observed localization of FLAG-tagged FAM98C (see **Fig. 4C and 5C**), it indicates that the C-terminal amino acid sequence of FAM98C is at least able to provide nuclear localization. Further nucleolar localization could be either prevented by the remaining sequence of FAM98C or by other components of the tRNA-LC.

4) In all depicted Northern blots, the levels of mature tRNA seem surprisingly low compared to intron-containing pre-tRNA. The authors should comment on that.

We thank the reviewer for raising this very valid concern. We believe that the low levels of mature tRNAs observed in our northern blots against intron-containing tRNAs are caused by chemical modifications present on mature tRNAs but absent in pre-tRNA and tRNA fragments. These modifications likely interfere with the binding of the probe, resulting in decreased affinity and leading to a low signal. With such hypothesis in mind, we tried to optimize the detection of mature tRNAs for Tyr-GTA and Ile-TAT by:

- Trying two additional probes for each tRNA that bind more towards the 5’ region of the tRNA.
- Lowering the hybridization temperature (40 °C or 45 °C instead of 50 °C).

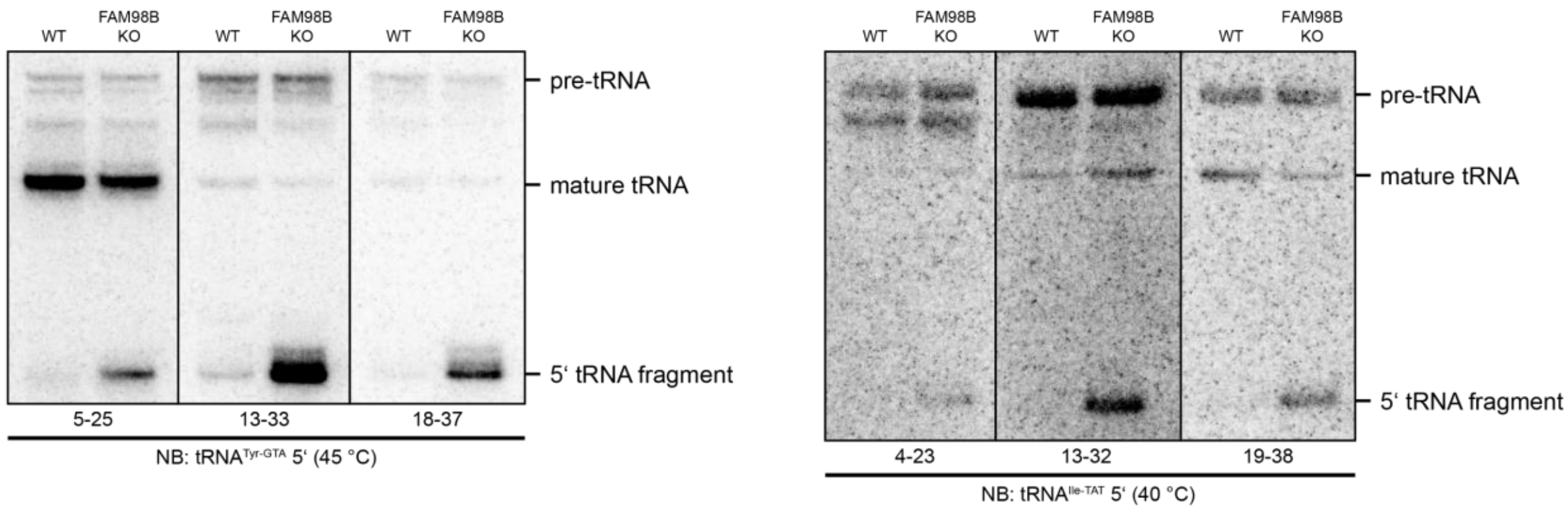

Testing new tRNA probes for northern blots. New DNA probes targeting the 5’ regions of tRNA$^{Tyr-GTA}$ and tRNA$^{Ile-TAT}$ were hybridized against total RNA from HEK FITR WT and FAM98B KO cells (5 µg per lane). Tyr probes were hybridized at 45 °C; Ile probes at 40 °C. Binding positions (in nucleotides) are depicted below the blots. The old probes (Tyr 18-37 and Ile 19-38) were used as control. Compared northern blots were exposed onto the same screen and got the same contrast adjustments.

Both new Tyr-GTA probes (5-25 and 13-33) detected the same bands as the original probe (18-37), but with large differences in their intensities (45 °C hybridization instead of 50 °C for all three tested probes):

- <u>Tyr-GTA 5-25:</u> high signal for mature tRNAs; good signal for tRNA fragments and pre-tRNAs.
- <u>Tyr-GTA 13-33:</u> very high signal for tRNA fragments; good signal for pre-tRNAs and weak signal for mature tRNAs (still better than the old probe).
- <u>Tyr-GTA 18-37 (old):</u> high signal for tRNA fragments; weakest signal for pre- and mature tRNAs.

From these results we feel that our initial guess was correct, and that the original probe was sub-optimal to detect mature tRNAs due to modifications interfering with the binding. Interestingly, we did not observe the same for Ile-TAT, where the new probes either performed similarly or even worse than the original probe. However, by merely lowering the hybridization temperature down to 40 °C, the signal for the mature Ile-TAT tRNA band increased to levels that could be used for quantification (see figure below).

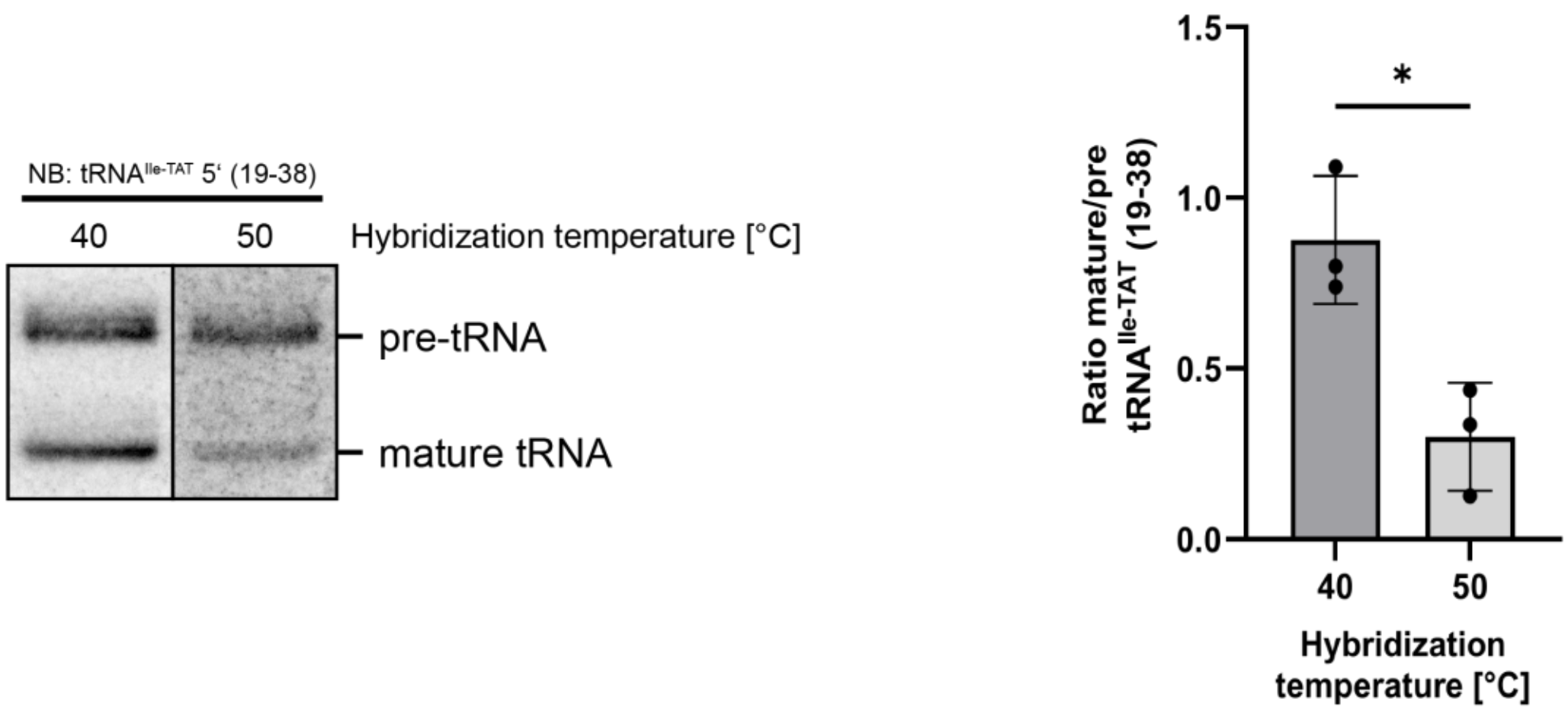

Lowering the hybridization temperature of the original probe for $tRNA^{Ile\text{-}TAT}$ by 10 °C increases the signal of the mature tRNA. Comparison of the signal strength from mature $tRNA^{Ile\text{-}TAT}$ bands when performing hybridizations at 40 and 50 °C. 5 µg total RNA from HEK FITR WT cells was used per lane. The signal from the mature tRNA was normalized to the signal of the pre-tRNA. The experiment was performed in triplicates. Representative blots are displayed.

In essence, our major problem for confidently quantifying mature tRNA levels of tRNA Ile-TAT and Tyr-GTA was the very low signal to noise ratio. We have now solved that problem with the new probes and optimizing hybridization conditions. We therefore repeated the northern blots for the FAM98 and ASW KOs with the new conditions/probes: Tyr-GTA 5-25 probe for 5' Tyr-GTA tRNA detection at 45 °C, an additional probe for detecting 3' Tyr-GTA tRNA at 45 °C, and the originally used Ile-TAT probe for detecting 5' Ile-TAT tRNA at 40 °C. With these new conditions and probes, we managed to detect defects in the levels of mature tRNAs Tyr-GTA and Ile-TAT in FAM98B KO cells (see **Fig. 2B and Supplementary Fig. 2D**), matching what has been reported recently for FAM98B KO cells (*Yang et al. 2025, Science*). However, ASW KO cells did not show defects in the levels of mature tRNAs (see **Fig. 3B and Supplementary Fig. 3A**), indicating that the defect is not as severe as in FAM98B KO cells. We speculate that the reduction of other tRNA-LC subunits in FAM98B KO cells, on top of the nuclear exclusion, might underpin the severity of the pre-tRNA splicing phenotype in FAM98B KO cells. In terms of technology and protocols, we have learnt that both sequences and temperatures could be adjusted to monitor different steps during the biogenesis of tRNAs, depending on the focus of the experiment. We therefore thank the Reviewer for pushing us in this direction.

5) The authors did not detect an impact on XBP1 mRNA splicing in the various FAM98 KO cell lines. It would be interesting to see if a combination of FAM98A KO with RTCB-NLS or FAM98B overexpression would show any reduction in XBP1s.

We thank the reviewer for suggesting this experiment. In our hands, *XBP1*-mRNA splicing seems to be extremely resilient against manipulating the composition and localization of the tRNA-LC. We now tried to disrupt *XBP1*-mRNA splicing by overexpressing ASW in FAM98A KO cells, as we believe that transport into the nucleus via ASW is the major route for the nuclear import of the tRNA-LC. However, additional overexpression of ASW in FAM98A KO cells did not impair *XBP1*-mRNA splicing (see **Supplementary Fig. 6**), suggesting that cytoplasmic tRNA-LC is not sufficiently depleted to cause defects in *XBP1*-mRNA splicing. From our experimental data, the tRNA-LC appears to be a very efficient enzyme and thus, even minimal levels might be sufficient to sustain *XBP1*-mRNA splicing in the cytoplasm. To further deplete cytoplasmic tRNA-LC, and following the suggestion of the Reviewer, we generated a HEK FITR cell line in which we added the c-MYC-NLS onto the N-terminus of endogenous RTCB (henceforth NLS-RTCB; the same sequence as the expression construct in **Fig. 5C** but without the FLAG-HA-tag) and, on top of that, we knocked out FAM98A. With this approach we were finally able to see a defect in *XBP1*-mRNA splicing (see **Fig. 5F, G**). Interestingly, NLS-RTCB cells still containing FAM98A showed no defects in *XBP1*-mRNA splicing (see **Fig. 5G**) indicating that FAM98A might counteract nuclear import of the complex.

6) Similarly, the levels of mature tRNA are not reduced in any of the KO cell lines, despite fragment accumulation in some cases. While the authors fully acknowledge and address these aspects in the discussion, the inclusion of orthogonal sequencing approaches would strengthen the findings. As mentioned above, the low levels of mature tRNA in the Northern blots complicate the interpretation further.

We thank the reviewer for this suggestion. As commented above, optimizing northern blots in terms of probes and conditions allowed us to also quantify mature tRNA levels for intron-containing tRNAs Tyr-GTA and Ile-TAT very effectively. As a result of such optimization, we were able to detect a significant reduction of mature, spliced tRNAs in FAM98B KO cells (see above, as well as **Fig. 2B** and **Supplementary Fig. 2D**). We fully acknowledge that tRNA sequencing would be informative, but we believe that the optimized northern blots – revealing identity and quantity – together with our reported sequencing of similar 5' tRNA fragments (*Hanada and Weitzer et al. 2013, Nature*) should address the concern of the reviewer. While doing the revisions, we prioritized the experiments that in our modest opinion were the most critical, including some that were not mandatory but we still believed were important. Those experiments are detailed in this response letter and the new version of the manuscript. Once again, we apologize and hope that the optimization of the northern blots plus our previous paper suffices to satisfy the reviewer's concern on the identity, concentration and biogenesis of the tRNA species under investigation.

Minor points:

7) The authors should consider changing "destabilized" to something more neutral when describing the results in Fig. 2A (page 5, line 126). Primarily, they observed reduced levels. Whether the protein levels are reduced by increased degradation/destabilization, by some feedback mechanisms leading to reduced expression, or a combination of multiple effects, is difficult to discern. Does overexpression of the A or C paralogs return the levels of all subunits except for ASW back to normal?

We thank the reviewer for this suggestion and fully agree that the term "destabilized" is not appropriate. We now performed the requested rescue experiments, overexpressing either FAM98A (full-length or truncated), FAM98B (full-length or truncated) or FAM98C in FAM98B KO cells (see **Supplementary Fig. 2C**). Levels of DDX1, RTCB and CGI-99 were increased to different extents when expressing FAM98C, FAM98B (both constructs) and truncated FAM98A. Interestingly, expressing the full-length FAM98A construct did not visibly increase the levels of DDX1, RTCB and CGI-99, even though this construct was expressed at the same level as the truncated FAM98A construct. We hypothesize that also endogenous FAM98A would not contribute significantly to the levels of FAM98A-containing tRNA-LC. This would also explain why a KO of FAM98A does not show a reduction in the levels of other core tRNA-LC subunits. Ultimately, and as expected, ASW levels only increased when overexpressing either of the FAM98B constructs.

Reviewer #2 (Remarks to the Author):

This study demonstrates a role for the ASW subunit of the vertebrate tRNA ligase complex (tRNA-LC) in localizing the ligase to the nucleus where tRNA splicing occurs and shows that ASW binding to complex subunit FAM98B (one of three FAM98 paralogs) specifically leads to nuclear localization of FAM98B-containing ligase complexes. By contrast, complexes containing paralog FAM98A reside in the cytoplasm where they perform noncanonical mRNA splicing in the unfolded protein response. In a clever maneuver, the authors show that appending an NLS to RtcB restores nuclear localization of tRNA-LC, and reverses the accumulation of 5' tRNA fragments, in cells deleted for ASW. These are important findings.

We thank the reviewer for the summary and acknowledgement of our findings.

Major points:

8) CRISPR-knockouts of the FAM98 paralog genes and ASW in 293 cells reveal that deletion of FAM98B or ASW specifically leads to accumulation of tRNA 5' exon fragments. It is most noteworthy that these KO cells do not have lower steady-state levels of the mature spliced forms of these same tRNAs. This would seem to call into question the assertion (line 123) that "FAM98B and ASW are ESSENTIAL for effective pre-tRNA splicing". Given that tRNA splicing is essential per se for cell viability, it would behoove the authors to show data regarding the growth properties of the cell lines in which ASW and FAM98B are deleted. Is there a defect in protein synthesis?

We thank the reviewer for the feedback as well as the logic and well supported concerns. Regarding the steady-state levels of mature, spliced tRNAs, we were able to optimize our probing conditions, which enabled confident quantification. These quantifications show a reduction of mature levels of tRNA Tyr-GTA and Ile-TAT in FAM98B KO cells (see **Fig. 2B, E** and Supplementary **Fig. 2D, H**), which now matches what has recently been reported for FAM98B KO cells (*Yang et al. 2025, Science*). For details of these optimizations, we would kindly refer to the detailed response provided to reviewer #1, with some extra experimental evidence.

We further agree with the reviewer that the wording "FAM98B and ASW are essential for effective pre-tRNA splicing" is inappropriate and should have led to a more severe phenotype, especially for ASW KO cells. We thus rephrased it to "FAM98B and ASW are required for efficient pre-tRNA splicing" and performed additional experiments. As requested by the reviewer, we investigated the growth of the different FAM98 KO and ASW KO cell lines using an Incucyte live-cell analysis system (see **Supplementary Figs. 2E, F and 3C, D)**. FAM98C and ASW KO cells had no detectable growth defects. Both FAM98A KO clones showed a longer lag phase, but ultimately achieved growth rates similar to WT cells. Both FAM98B clones showed longer lag phases and one of them displayed a reduced growth rate. However, the other FAM98B KO clone achieved growth rates similar to WT cells. We further investigated global protein synthesis rates in FAM98 KO cells by performing puromycin-incorporation assays (see **Supplementary Fig. 2G, H**). Here, neither KO condition showed a significant reduction in global protein

synthesis. However, this approach might not be sensitive enough to detect protein synthesis defects that may occur specifically for proteins that are rich in codons that require decoding by spliced tRNAs. Last but not least, it is ultimately possible that cell culturing conditions might not fully recapitulate *in vivo* requirements, i.e. the growth of FAM98 and ASW KO cells in a tissue environment.

9) Taken at face value, the results suggest that cells are able to somehow compensate for the loss of ASW and FAM98B and continue to splice intron-containing tRNAs. How might this happen? Does a residual level of nuclear localization of tRNA-LC sustain tRNA splicing in their absence? The authors favor this idea in the Discussion. (Alternatively, can some level of tRNA splicing occur in the cytoplasm when ASW and FAM98B are missing?)

We thank the reviewer for these interesting questions. We hypothesize that a small fraction of nuclear tRNA-LC, the one containing FAM98C, is able to splice enough tRNAs for the cell to divide in the absence of FAM98B or ASW. In addition, and during cell division, the nuclear-cytoplasmic compartmentalization transiently disappears, thus allowing forms of the tRNA-LC without FAM98B/ASW to encounter and ligate tRNA exon halves. Furthermore, after regeneration of the nuclear envelope, it will take time to deprive the nucleus from enzymatically active, non-FAM98C-containing tRNA-LC. As FAM98B KO cells suffer from overall reduced levels of other tRNA-LC subunits, this residual pre-tRNA splicing would be lower than in ASW KO cells, which could explain the reduced steady-state levels of mature, spliced tRNAs. We also believe that the strength of the pre-tRNA splicing defect upon loss of FAM98B or ASW depends on the cellular context. Cells that divide slower or do not divide at all (which is closer to the actual *in vivo* situation for many tissues) should experience a more severe phenotype. This is indeed the case for the loss of FAM98B, where liver and brain are affected to different degrees (*Yang et al. 2025, Science*).

We do not fully exclude the possibility that pre-tRNA splicing could take place in the cytoplasm, but we consider it highly unlikely. Firstly, subunits of the tRNA-LC required for catalysis are present in the cytoplasm but the substrate – tRNA halves generated by the TSEN complex – arise in the nucleus. Secondly, only nuclear exclusion of the tRNA-LC leads to the accumulation of tRNA fragments as a sign of impaired or inefficient ligation (*Asanović et al. 2021, Mol Cell; Hanada and Weitzer et al. 2013, Nature*), while cytoplasmic depletion does not. This further implies that the fragments originate in the nucleus and that nuclear tRNA-LC ligates those to generate an exportable, mature tRNA. That being said, we cannot completely exclude that those tRNA fragments could at some point reach the cytoplasm and be ligated, for instance when fragments accumulate due to defective nuclear pre-tRNA splicing, like from the loss of ASW or FAM98B.

10) The accumulation of 5' tRNA fragments is taken as synonymous with a primary defect in exon ligation. I would suggest strongly that the authors also assay by Northerns the fate of the 3' exons. Also, it would be very valuable to separate nucleus and cytoplasm and determine the compartment in which the 5'-tRNA fragments accumulate.

We thank the reviewer for suggesting these experiments. We now probed total RNA from FAM98 and ASW KO cells for the 3' half of tRNA Tyr-GTA (see **Fig. 2B, 3B and Supplementary Fig. 2D, 3A**). Similar to 5' tRNA fragments, we did observe an accumulation of 3' tRNA fragments in FAM98B and ASW KO cells, thereby validating them being a readout for a defect in tRNA exon ligation.

We further tried different methods of fractionating ASW KO cells into cytoplasm and nucleus, mainly differentiating between detergent-based and purely hypotonic-mechanical fractionation, to investigate the distribution of the different tRNA intermediates within the cell. Detergent-based fractionation (REAP, *Suzuki et al. 2010, BMC Res Notes*) seemed very leaky in our hands, at least for tRNAs, as only minor amounts of mature tRNAs and no pre-tRNA molecules were detected in the nuclear fraction; all other species, including most of the mature tRNAs, were found in the cytoplasm (see figure below, **panel A**).

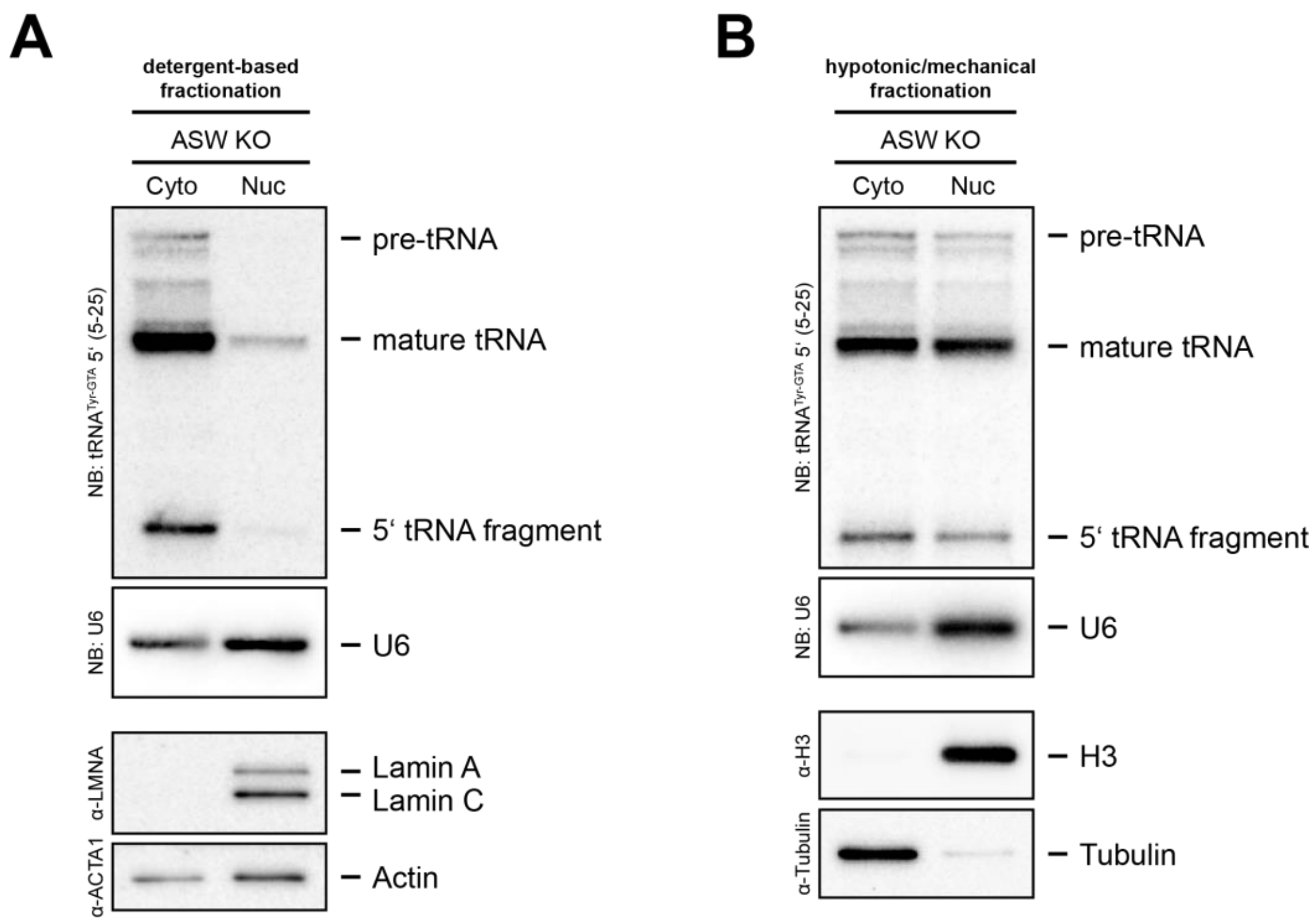

Subcellular distribution of the different intermediates of tRNA Tyr-GTA in ASW KO cells when using different nuclear-cytosolic fractionation methods. HEK FITR ASW KO cells were fractionated into cytosol and nucleus with different fractionation methods (detergent-mediated or hypotonic-mechanical lysis). Equal parts of the fractions were analyzed side-by-side with western and northern blotting to determine the distribution of $tRNA^{Tyr\text{-}GTA}$ as well as markers for fractionation cleanliness (snRNA U6, LMNA, ACTB, H3, Tubulin).

Fractionation based on hypotonic swelling and mechanical lysis (adapted from *Mayeda and Krainer 1999, Methods Mol Biol.*) gave radically different results, at least for tRNAs (see figure above, **panel B**). Here, all tRNA intermediates were found in both fractions and all of them were slightly, but equally, more present in the cytoplasm. These results suggest that pre-tRNAs as well as tRNA fragments would get

exported out of the nucleus and would accumulate more in the cytoplasm. However, as these results are very controversial and we do not have further supporting evidence for them, we are not confident with claiming that this is indeed the case. We also want to highlight that caution must be taken when interpreting fractionation results, as not every marker protein or marker RNA will be a reliable control for the leakiness or cleanliness of the fractionation. snRNA U6 is a prime example for this, as it was enriched in the nuclear fraction of both fractionation approaches, but the tRNA distributions were severely different. In this case, we believe that U6 is less prone to leakiness due to the massive size of the spliceosome.

11) Is it possible that 5' fragments accumulate because of failure to repair tRNA breaks inflicted "off-pathway" from the cleavage of pre-tRNA by TSEN? Precise mapping the 3'-ends of the 5'-fragments (by Seq) would provide valuable information in that regard, i.e., whether the 3' ends correspond to the cleaved exon-intron junction.

We thank the reviewer for this comment and concern. As of now, we cannot fully exclude that the fragments exclusively arising from intron-containing tRNAs (see also below) result from tRNA cleavage not coming from TSEN. However, there are observations that would go against them being the result of a cleavage other than from TSEN:

a) We specifically, and only see fragments of intron-containing tRNAs accumulating when ASW or FAM98B are absent. The only known, stress-induced cleavage event of tRNAs is executed by angiogenin, but angiogenin cleaves at the anticodon loop of mature tRNAs, thereby originating shorter 5’ tRNA fragments that miss the 5’ leader sequence. By targeting mature tRNAs, angiogenin ultimately targets many different tRNAs and is not specific for those that undergo splicing. A major target of angiogenin is tRNA Gly, which is why we chose to blot for this tRNA as control in our northern blots of the different FAM98 KOs. We never detected fragments of tRNA Gly in any of our FAM98 KO northern blots which indicates that angiogenin is not active in those cells and thus cannot be the cause of the detected fragments.
b) The fragments that result from ASW or FAM98B loss have the same size as the ones that result from exposing the cells to oxidative stress (menadione; see figure below). We have previously shown that the fragments that are caused by oxidative inactivation of the tRNA-LC (H_2O_2 or menadione) are the same as the ones that result from depletion of RTCB or PYROXD1 or by inactivating CLP1 (*Asanović et al. 2021, Mol Cell; Hanada and Weitzer et al. 2013, Nature*). Back then, we thoroughly characterized tRNA fragments by sequencing, finding an over-representation of the 5’ fragments corresponding to tRNA Tyr-GTA, and showed that it is composed of a 5’ leader sequence followed by the 5’ exon sequence, exactly ending at the exon-intron boundary, as would be the case for TSEN cleavage.

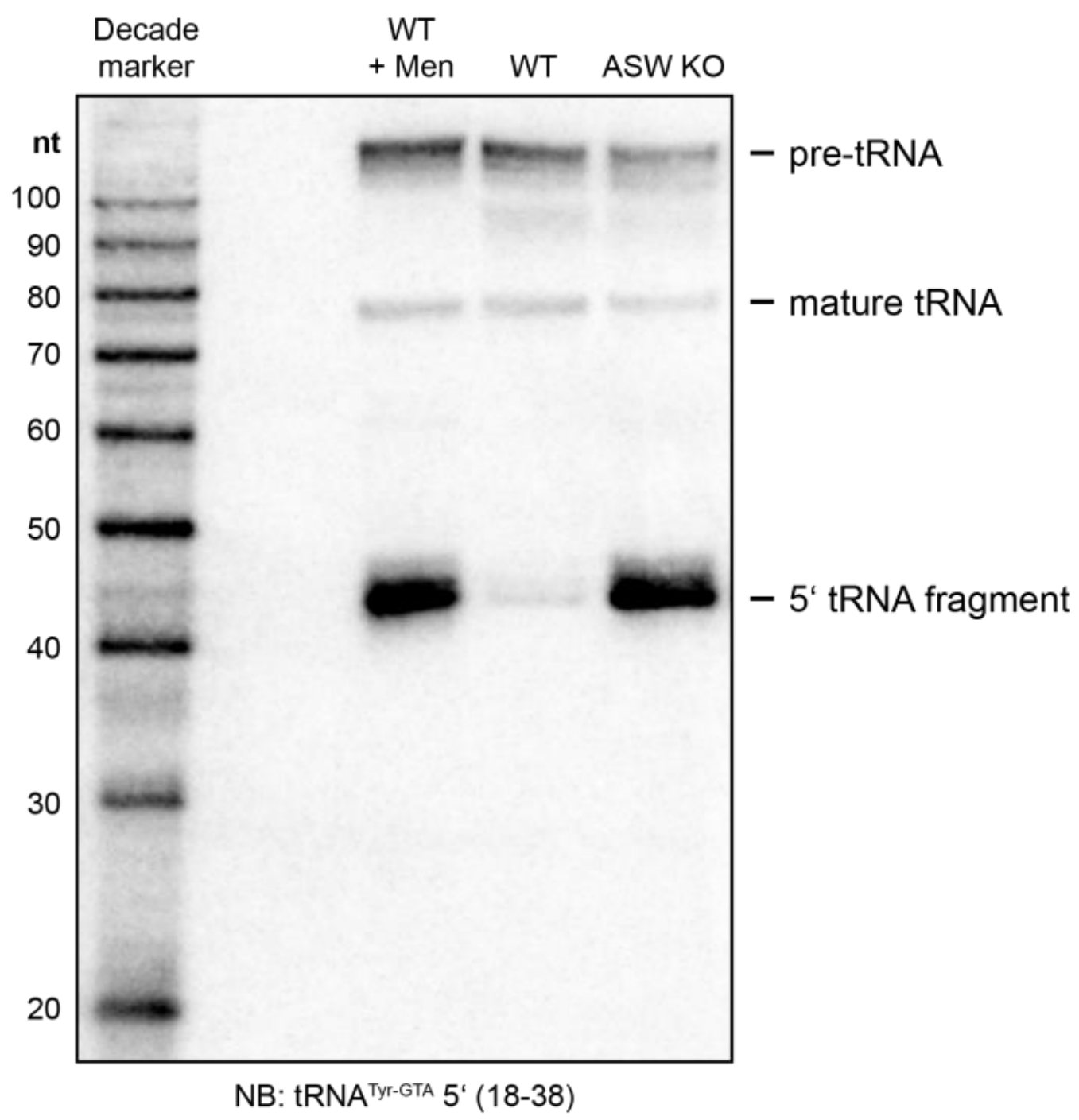

5' tRNA fragments of tRNA Tyr-GTA caused by short-term oxidative stress and loss of ASW have the same size. Steady-state levels of tRNA$^{Tyr\text{-}GTA}$ as well as its precursors in HEK FITR WT and ASW KO cells. As a positive control for inhibition of the tRNA-LC, WT cells were treated with 40 µM menadione (Men) for 1 h prior to RNA isolation. We used the Tyr 18-37 probe to favour detection of the fragments. 5 µg total RNA were loaded per lane.

12) Given that the Northern analysis performed on total RNA, it is surprising that the steady state levels of pre-tRNA consistently exceed those of the mature tRNA. How to explain that seeming anomaly?

We thank the reviewer for bringing up this concern. We believe that the low observed levels of mature tRNAs in our northern blots against intron-containing tRNAs is caused by modifications of the mature tRNAs, close to or within the anticodon, that are not present in the pre-tRNA and 5' tRNA fragments. These would interfere with the binding of our DNA probes and thus decrease affinity and consequently the observed signal. We could indeed show that is the case for tRNA Tyr-GTA, where using probes that bind more towards the 5' end of the tRNA strongly increased the detected levels of the mature form. For more details of these dissections and optimizations, we would kindly refer to a section above regarding the same question from reviewer #1.

Minor points:

13) Lines 117-119: "band" and "bands" are inappropriate slang terms. Change to "polypeptide" and "polypeptides".

We thank the reviewer for this remark. We changed the mentioned passages to the appropriate terms.

Reviewer #1 (Remarks to the Author):

During revision, the authors of Leitner et al. have extensively addressed reviewers' comments and added new experiments, data and analyses. I have re-evaluated the revised version of the manuscript and the detailed point-by-point response provided by the authors. The authors have thoroughly addressed my remarks. I especially appreciate the systematic optimization of the Northern blotting protocols and nuanced discussion of the results.
I support publication of this manuscript in Nature Communications and commend the authors on their work.
Jirka Peschek

We thank the reviewer for the kind acknowledgement of our revision experiments, our improved manuscript and our work.

Reviewer #2 (Remarks to the Author):

The revised paper is improved as the authors have now performed additional experiments to optimize tRNA detection by Northern blotting at reduced annealing temperature (Fig. 2B). This resolved major concerns with the original data that seriously underestimated the level of mature tRNA while showing accumulation of an unligated 5' tRNA half. As requested, they also now document the presence of 3' tRNA half. Requested assays of cell growth and protein synthesis have been added.

We thank the reviewer for acknowledging the improvement of our manuscript due to our revisions.

It is unclear why they did not incorporate the technical improvement to redo the Northerns shown in Fig. 5A, B and E (seemingly unchanged from the original version, where mature tRNA is nearly undetectable). Doing so would be a plus.

While we agree that redoing the northern blots with the optimized conditions for the samples of Figs. 5A, B and E would increase the quality of our data further, the added value would not be as significant as for the northern blots in Figs. 2B and 3B. We optimized the conditions to better detect the mature forms of spliced tRNAs, so that we can draw conclusions on changes of their levels. However, ASW KO cells do not display reduced mature tRNA levels and the experiments in Figs. 5A, B and E are rescue experiments in ASW KO cells. Therefore, in those experiments, our readout for the pre-tRNA splicing defect is the accumulation 5' tRNA-fragments, which are well detected by our initial conditions. The only added benefit would be detecting potential reductions in mature tRNA levels that could happen in the conditions where the pre-tRNA splicing defect in ASW KO cells is worsened, for example when expressing FLAG-HA-RTCB or FLAG-HA-FAM98A. Due to the initial northern conditions being sufficient for our main experimental readout in Figs. 5A, B and E as well as the effort and time it would take to redo all these northern blots, we decided to focus on other experiments that were more urgent and important for the revision of our manuscript.
